# Spinal nociceptive denervation impedes subsequent chronic autonomic remodeling after myocardial infarction in male swine

**DOI:** 10.1038/s41467-026-75759-2

**Published:** 2026-07-22

**Authors:** Valerie Y. H. van Weperen, Jonathan D. Hoang, Neil R. Jani, Shail Avasthi, Christopher A. Chan, Kuan Cao, Zulfiqar A. Lokhandwala, Maryam Emamimeybodi, Karim Atmani, Marmar Vaseghi

**Affiliations:** 1 UCLA Electrophysiology Programs, Division of Cardiology, Department of Medicine, University of California, Los Angeles (UCLA), Los Angeles, CA USA; 2 Molecular, Cellular, and Integrative Physiology Interdepartmental Programs, UCLA, Los Angeles, CA USA

**Keywords:** Ventricular tachycardia, Cardiovascular diseases, Cardiology, Myocardial infarction

## Abstract

After chronic myocardial infarction (MI), pathological autonomic remodeling, including vagal dysfunction and sympathoexcitation, predisposes to ventricular arrhythmias (VT/VF). However, what underlies this functional and structural remodeling remains unknown. We hypothesized that spinal nociceptive afferent signaling initiates and perpetuates these pathological autonomic changes. We employed cervicothoracic epidural resiniferatoxin (RTX) to ablate spinal nociceptive neurons in male pigs before MI, and assessed autonomic and electrophysiological function four-to-six weeks post-infarction. Compared to vehicle-treated infarcted animals, epidural RTX attenuated the loss of vagal tone and baroreflex sensitivity, reduced spinal cord inflammation, glial activation, and circulating stress and inflammatory markers, and stabilized electrophysiological parameters, lowering VT/VF inducibility. In a separate cohort, acute C7–T1 nociceptive afferent ablation after chronic MI acutely restored vagal function and decreased VT/VF inducibility. This study demonstrates that cervicothoracic spinal nociceptive afferents significantly contribute to MI-induced autonomic remodeling and VT/VF, providing novel insight into the mechanisms underlying sympathovagal imbalance after MI.

## Abstract (web summary)

Autonomic remodeling after myocardial infarction predisposes ventricular arrhythmias. Ablating spinal cervicothoracic nociceptive afferents prior to infarction prevents this pathological neural remodeling and reduces arrhythmia susceptibility.

## Introduction

Sympathoexcitation and vagal dysfunction occur after chronic myocardial infarction (MI), predispose to ventricular arrhythmias (VT/VF) and result in progression of heart failure^1,2^. Heart failure and MI have been reported to be associated with significant structural and functional pathological autonomic remodeling, including glial activation and inflammation in the peripheral autonomic ganglia^3,4^, which are thought to play a role in the sympathovagal imbalance post-MI and contribute to electrophysiological instability and VT/VF^5–8^. In addition, increased cervicothoracic spinal nociceptive afferent neurotransmission post-MI has been reported to reflexively amplify sympathetic efferent outflow^9–13^. Therefore, targeting these spinal afferent fibers/neurons, for instance, with resiniferatoxin (RTX), an ultrapotent and specific agonist of the transient receptor potential vanilloid 1 receptor (TRPV1)^14–21^, could represent an important therapeutic target. RTX ablates TRPV1-expressing nociceptive neurons via rapid and prolonged influx of calcium ions, leading to neuronal cytotoxicity and cell death^22–28^. Consistent with this, a prior study showed that the ablation of cardiac TRPV1 neurons via direct application of RTX on thoracic dorsal root ganglia (DRG) reduced acute ischemia driven ventricular arrhythmias in a healthy porcine model^29^. Yet the effect of spinal afferent interruption with RTX on ventricular arrhythmias has not been evaluated beyond the acute MI window, even though ventricular arrhythmias due to chronic autonomic and myocardial remodeling remain an important cause of sudden cardiac death (SCD) long after the acute event^1,30,31^. Hence, in this study, we first sought to assess if *chronic* spinal nociceptive ablation is an important contributor to the ensuing autonomic remodeling that leads to electrophysiological heterogeneity and ventricular arrhythmias with chronic MI. To accomplish this, we administered epidural RTX prior to MI at the C7-T1 levels to target cervicothoracic spinal TRPV1 nociceptive afferent fibers and neurons. Four-to-six weeks after chronic MI, we evaluated (i) tissue structural and neurochemical changes, (ii) functional autonomic responses, and (iii) electrophysiological stability in chronically infarcted pigs which received RTX *versus* vehicle. We next sought to evaluate the potential therapeutic value of spinal nociceptive ablation for patients with refractory VT/VF after the acute phase of MI, when chronic pathological autonomic remodeling has already developed. To that end, we tested whether *acute* C7-T1 epidural RTX ablation of spinal nociceptive afferents in the established post-MI setting (4–6 weeks post-MI) would be sufficient to improve autonomic balance and reduce VT/VF burden.

## Results

**Fig. 1:**
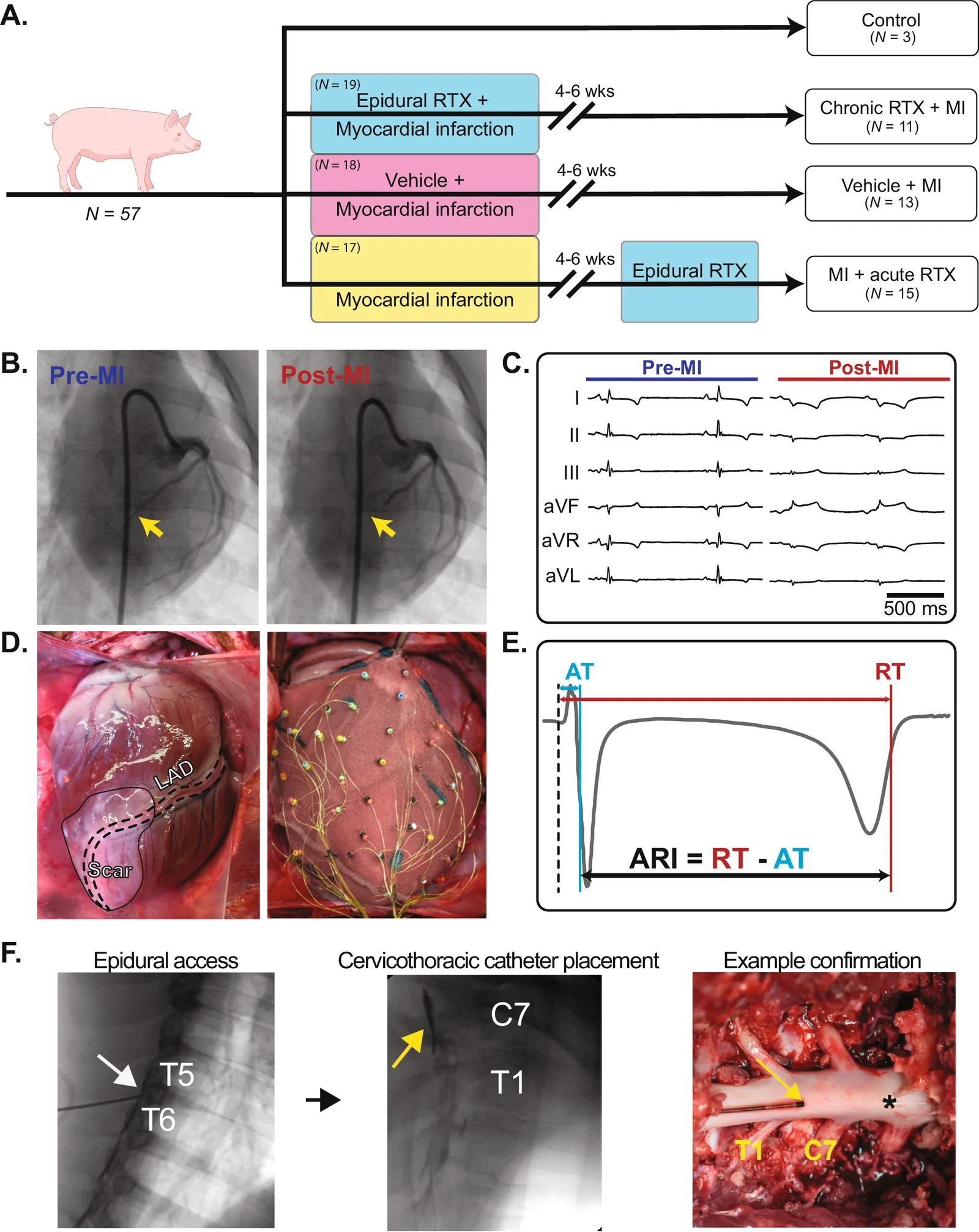
Experimental protocol. **A** Schematic representation of the experimental groups and their respective interventions. **B** Percutaneous creation of myocardial infarction (MI) via injection of microspheres in the distal left anterior descending (LAD) coronary artery after percutaneous angioplasty balloon inflation. Following injection, flow is significantly decreased in the mid and distal LAD (red arrows) and **C** ST-segment elevation is observed on the surface ECG. **D** At the time of terminal studies, a 56-electrode sock was placed around the ventricles to record local unipolar electrograms, from which **E** repolarization time (RT) and activation time (AT) were measured and activation recovery interval (ARI) calculated in all infarcted animals. **F** For epidural injections, an epidural catheter (white arrow) was advanced to the C7-T1 dorsal epidural space and position confirmed by contrast injection under fluoroscopic guidance. In terminal procedures involving the group of animals that underwent acute epidural RTX injections after chronic MI, catheter position was secondarily confirmed at the end of experiments by cut down to the epidural space and removal of epidural fat. Asterisk placed at cranial aspect of the spinal cord.

To determine if spinal nociceptive (TRPV1) afferent signaling is an underlying contributor to pathological structural and functional autonomic remodeling after MI, these neurons and their fibers  were ablated by cervicothoracic (C7-T1) epidural administration of RTX (Fig. 1). RTX was administered at the C7-T1 epidural level, as the majority of *cardiac* sensory innervation has been shown to pass through the C7 and upper thoracic DRG and spinal horns^32^. To characterize the safety, efficacy, and pharmacodynamic profile of RTX, we first administered epidural RTX to three healthy control animals. In these experiments, we primarily focused on changes in heart rate (HR) and left ventricular systolic pressure (LVSP)—parameters (or their surrogates) that can be readily assessed intra-operatively in humans without the need for intraventricular catheters. After RTX administration, an initial sympathoexcitatory response was observed in these animals, characterized by increased HR and LVSP, followed by stabilization of both hemodynamic parameters over the next 30–60 min (Supplementary Fig. 1). Accordingly, for the subsequent chronic studies, myocardial infarction was induced 2–3 h after C7-T1 epidural RTX (cRTX; *n* = 11) or epidural saline (vehicle; *n* = 13) administration.

Hemodynamic profiles of animals treated with epidural RTX prior to MI (cRTX) were comparable to those of the three pilot, healthy control animals (Supplementary Fig. 2), and showed that animals reached a steady hemodynamic state approximately 1 h after RTX administration. Comparison of MI-related mortality in cRTX versus vehicle-treated animals showed similar survival rates between these groups (Supplementary Fig. 3). However, VT/VF incidence in the acute-MI phase was higher in cRTX animals compared to vehicle-treated animals (Supplementary Fig. 3), possibly due to residual sympathoexcitatory effects associated with cervicothoracic epidural RTX administration.

Finally, we sought to evaluate the pathophysiological role of nociceptive afferent signaling on subsequent functional chronic autonomic remodeling and VT. To this end, the efficacy of epidural RTX for ablating nociceptive afferents and the associated hemodynamic and autonomic responses, neural activity, and electrophysiological parameters were then assessed in both cRTX and vehicle-treated infarcted animals 4–6 weeks after MI. We also examined the effect of RTX-mediated nociceptive neuron ablation on (i) plasma proteomic profiles and inflammatory markers, (ii) markers of glial activation, and (iii) neuroinflammation in the spinal cord at both the C7-T1 as well as the lower thoracolumbar regions (T12-L1, distal anatomical control).

### Selectivity and potency of RTX for cervicothoracic spinal afferent nociceptive neurons

**Fig. 2:**
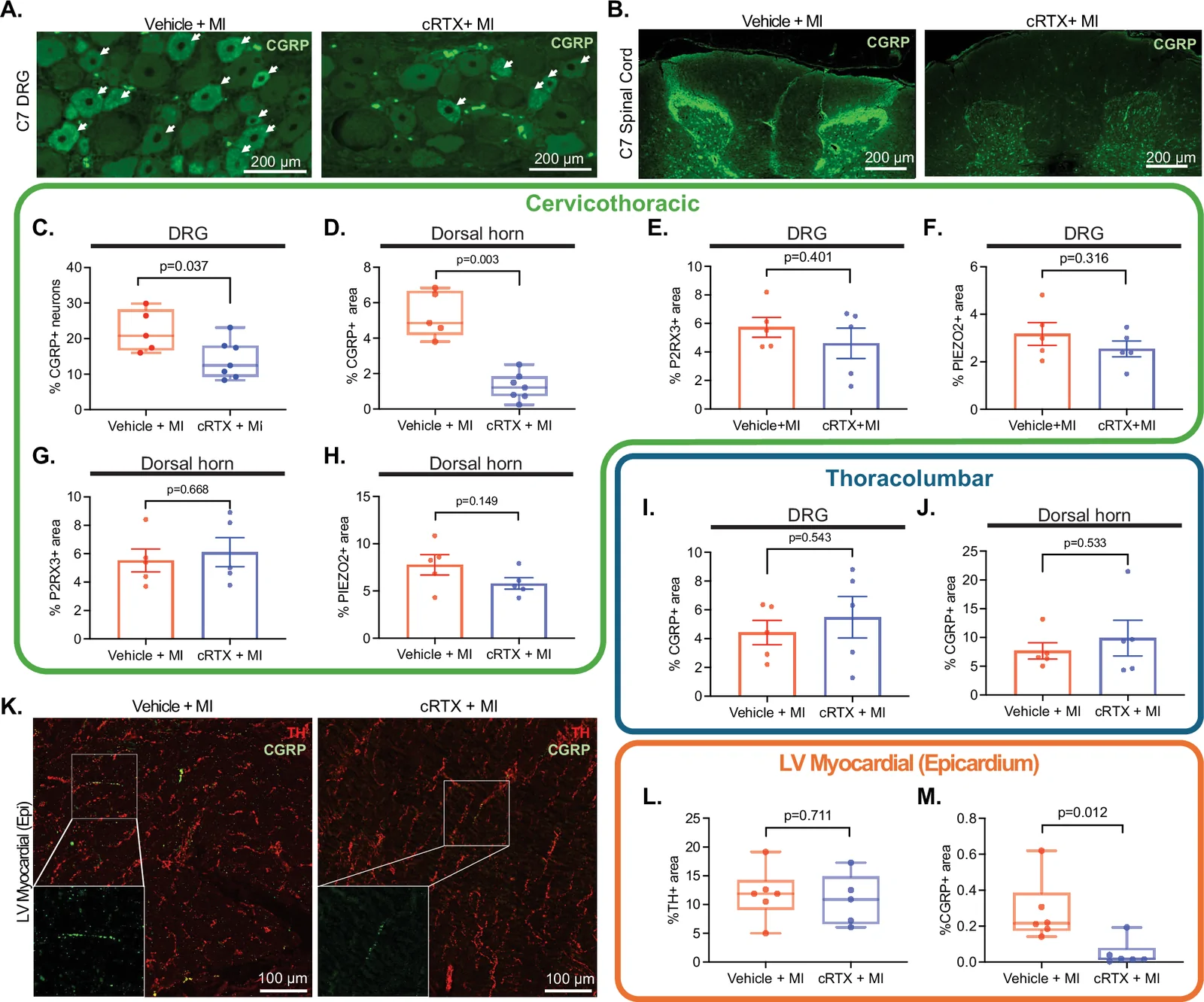
Immunohistochemical confirmation of successful ablation of cervicothoracic spinal nociceptive neurons and fibers. **A** Representative images of calcitonin gene-related peptide (CGRP) immunostaining of the dorsal root ganglia, and **B** spinal cord dorsal horn from a chronic vehicle and an epidural cRTX (chronic resiniferatoxin-treated) animal. **C** A significant decrease in CGRP expressing neurons in the dorsal root ganglia (DRG) and **D** dorsal horn of epidural cRTX (*n* = 7) *vs* vehicle animals (*n* = 5) is observed, confirming successful chemoablation of spinal nociceptive cardiac afferents in animals that received RTX. **E** Expression of purinergic receptor P2X (P2RX3) and **F** Piezo-type mechanosensitive ion channel component 2 (PIEZO2) in the cervicothoracic DRG was unchanged between cRTX (*n* = 5) *vs* vehicle treated, infarcted animals (*n* = 5). Similarly, expression levels of **G** P2RX3 and **H** PIEZO2 in the cervicothoracic dorsal horn were not different between vehicle-treated (*n* = 5) versus cRTX animals (*n* = 5). **I** Comparison of CGRP expressing neurons in the thoracolumbar (T12-L1) DRGs, or **J** dorsal horn of vehicle-treated (*n* = 5) versus cRTX animals (*n* = 5), demonstrated no difference, suggesting that these lower spinal levels were unaffected by the local (C7-T1) administration of RTX. **K** Representative images of tyrosine hydroxylase (TH) and CGRP immunostaining at the level of the heart. **L** Expression of TH (by dopaminergic non-TRPV1 expressing neurons, and sympathetic efferents) is not different in the left ventricle (LV) of RTX (*n* = 5) *vs* vehicle-treated infarcted animals (*n* = 5), whereas **M** the immunoreactivity of CGRP is significantly lower in the RTX (*n* = 5) *vs* vehicle animals (*n* = 5). Epicardial CGRP expression was compared using the two-sided Mann–Whitney test; all other differences between vehicle and cRTX animals were assessed using the two-sided, unpaired Student’s *t*-test. Epidural vehicle-treated animals that subsequently were infarcted are denoted as Vehicle+MI and epidural RTX-treated animals that were subsequently infarcted are denoted as cRTX+MI. For all bar graphs, data are presented as mean values and error bars represent SEM. For each boxplot, the center line indicates the median, the upper and lower bounds represent the first and third quartiles, and the whiskers represent the minimum and maximum values observed. Source data are provided with this paper.

As most TRPV1-expressing nociceptive neurons co-express and release calcitonin gene-related peptide (CGRP)^33,34^, we used CGRP immunoreactivity (IR) as a marker for these nociceptive afferents. Spinal nociceptive signaling is mediated by cell bodies in the DRG, which synapse on second-order neurons in the upper lamina of the dorsal horn^35^. In this study,  we first evaluated whether administration of RTX prior to MI had resulted in chronic depletion of nociceptive fibers/neurons at the DRG and spinal hors in cRTX (*n* = 7) versus vehicle-treated (*n* = 5) animals (Fig. 2A–D). In cRTX animals, the percentage of CGRP-IR neurons in the C7-T1 DRG was significantly decreased (14 ± 2% *vs* 22 ± 3%, respectively, *p* < 0.05; Fig. 2C), and CGRP-IR in the dorsal horn was also significantly reduced compared to vehicle animals (1.2 ± 0.3% *vs* 5.3 ± 0.6%, respectively, *p* < 0.05; Fig. 2D), confirming successful chemoablation of nociceptive afferent fibers and neurons in cRTX animals.

Although prior literature has shown selectivity of RTX for TRPV1-expressing chemosensitive neurons^19–21^, we further confirmed the selectivity of RTX in our studies by evaluating two other major afferent populations: the chemosensitive purinergic P2RX3-expressing neurons and mechanosensitive PIEZO2-expressing neurons. Evaluation of the C7-T1 spinal cord DRG and dorsal horns showed that the percentage of PIEZO2- and P2RX3-immunoreactive afferents was similar between cRTX and vehicle-treated animals (Fig. 2E–H).

It has been shown that given the fatty nature of the epidural space, injection of anesthetics and compounds at various spinal levels, including RTX, rarely diffuses beyond 1–2 spinal levels^36–38^. However, to further confirm the anatomical and spatial selectivity of C7-T1 epidural RTX in the chronically infarcted animals, CGRP expression in the spinal cord dorsal horn and DRG at the cervicothoracic level (C7-T1) was compared to the thoracolumbar level (T12-L1). In accordance with other reports, we observed that CGRP expression was unchanged in the T12-L1 dorsal horn and DRG of vehicle-treated versus cRTX animals (Fig. 2I, J).

Finally, as spinal C7-T1 afferents chiefly innervate the heart^39^, we evaluated whether ablation of nociceptive neurons at this level with RTX would manifest as a loss of CGRP-expressing fibers at the level of the ventricular myocardium. Evaluation of LV myocardium showed that the density of CGRP-expressing ventricular fibers was reduced in epidural cRTX versus vehicle-treated animals (Fig. 2K–M). By contrast, the abundance of tyrosine hydroxylase (TH), a marker often used for evaluation of sympathetic *efferent* fibers, was unchanged, further underscoring the chemical selectivity of RTX for TRPV1-nociceptive neurons.

### Lack of detectable off-target effects with local epidural RTX

In line with the histological data indicating segmental selectivity in the effects of RTX, local epidural administration of RTX did not appear to cause any significant off-target effects, as no behavioral differences were noted between cRTX and vehicle-treated animals in the 4–6 weeks following MI. Furthermore, plasma levels of hepatic enzymes aspartate transaminase (AST) and alanine aminotransferase (ALT), which are clinically used to assess liver function, were similar between RTX- and vehicle-treated animals (Supplementary Fig. 4). Finally, weight gain in the 4-to-6-week period following vehicle or RTX treatment was similar between these groups (Supplementary Fig. 5).

### Longitudinal effects of RTX prior to MI on cardiac and autonomic remodeling

**Fig. 3:**
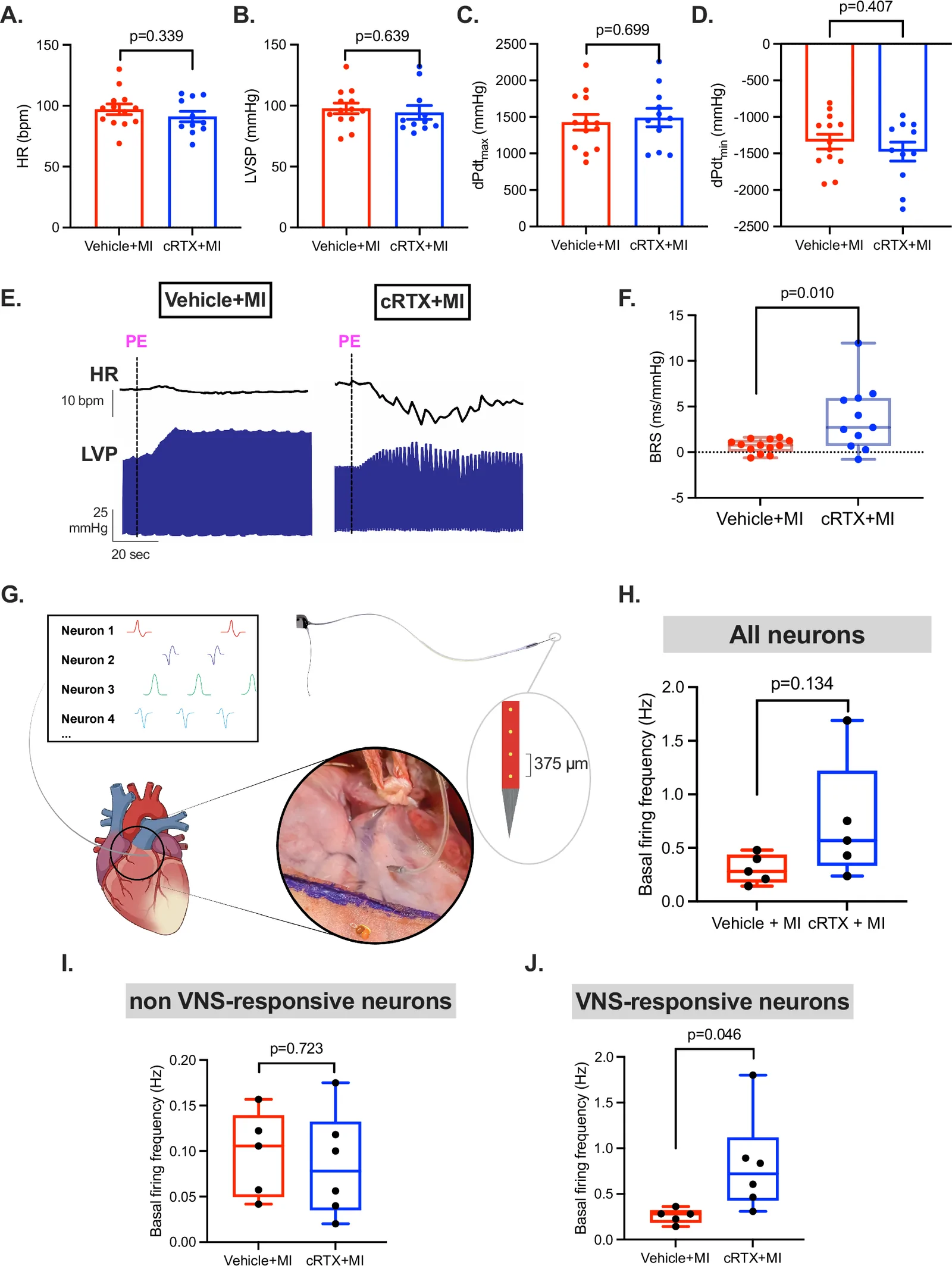
Effects of epidural RTX prior to MI on cardiac hemodynamics and vagal tone four to six weeks after MI. **A**–**D** Baseline hemodynamic parameters were not different between vehicle (*n* = 13) and epidural cRTX (*n* = 11) animals. **E** Example of phenylephrine-evoked baroreflex responses in a vehicle and epidural cRTX (resiniferatoxin-treated, infarcted) animal. **F** Baroreflex sensitivity (BRS) was significantly improved in epidural cRTX (*n* = 11) compared to vehicle animals (*n* = 13). **G** Schematic image of ventral interventricular ganglionated plexus (VIVGP) neural recordings and identification of VIVGP neurons. A magnified image of the neural recording electrode in a VIVGP is shown. The left atrial appendage is being retracted to fully expose the VIVGP. A picture of the neural probe and a schematic of the tip with the most distal 4 recording electrodes are shown. There was no difference in basal firing frequency of all (**H**) or non-VNS-responsive (**I**) neurons. **J** However, the basal firing frequency of VNS-responsive neurons (*e.g*., parasympathetic postganglionic neurons) was significantly higher in epidural cRTX (*n* = 6) compared to vehicle animals (*n* = 5). Differences in heart rate (HR), left ventricular systolic pressure (LVSP), inotropy (dP/dt_max_), lusitropy (dP/dt_min_), and neuronal firing between vehicle and epidural cRTX animals were assessed using the two-sided unpaired Student’s *t*-test, and BRS was compared by the two-sided Mann–Whitney test. For all bar graphs, data are presented as mean values and error bars represent SEM. For each boxplot, the center line indicates the median, the upper and lower bounds represent the first and third quartiles, and the whiskers represent the minimum and maximum values observed. PE: Phenylephrine. Epidural vehicle-treated animals that subsequently were infarcted are denoted as Vehicle+MI and epidural RTX-treated animals that were subsequently infarcted are denoted as cRTX+MI. Source data are provided with this paper.

Four-to-six weeks after MI, hemodynamic parameters including HR, LVSP, inotropy (dP/dt_max_), and lusitropy (dP/dt_min_) were assessed in cRTX (*n* = 11) and vehicle-treated (*n* = 13) animals. Baseline hemodynamic parameters were similar between these two groups of animals (Fig. 3A–D), suggesting no differences in resting hemodynamic function.

We then assessed vagal baroreflex sensitivity (BRS), a marker of parasympathetic function^40,41^, by evaluating responses to infusion of phenylephrine (an alpha-1 adrenergic agonist). BRS was significantly increased in cRTX (*n* = 11) compared to vehicle animals (*n* = 13; 3.7 ± 1.1 *vs* 0.7 ± 0.2 ms/mmHg, respectively, *p* < 0.05; Fig. 3E, F). These findings support the key contribution of spinal nociceptive afferents to post-MI vagal baroreflex impairment, which has been linked to increased SCD risk^40,42,43^.

To further confirm the impact of sympathetic spinal nociceptive afferent signaling on *efferent* vagal dysfunction post-MI, activity of intra-cardiac, postganglionic parasympathetic neurons in the ventral interventricular ganglionated plexus (VIVGP), a major source of ventricular parasympathetic innervation^44–46^, was measured in cRTX (*n* = 6) versus vehicle (*n* = 5; Fig. 3G) animals. We defined post-ganglionic parasympathetic neurons as neurons exhibiting statistically significant alterations in their vagal activity in response to low-level cervical vagus nerve stimulation (VNS)^47,48^. Low-level cervical VNS permits the assessment of neuronal activity without confounding hemodynamic changes^47–49^, enabling the classification and evaluation of neurons based on whether or not they receive vagal input. Spinal nociceptive afferent blockade prior to MI (cRTX animals) neither affected overall bulk firing of VIVGP neurons (Fig. 3H) nor the firing of VNS non-responsive neurons (Fig. 3I). By contrast, the baseline firing of VNS-responsive neurons (i.e., post-ganglionic cardiac parasympathetic neurons) was significantly higher in cRTX versus vehicle animals (Fig. 3J). Together with the BRS findings, these data suggest that interruption of cervicothoracic spinal afferents prior to MI preserved vagal function after chronic MI, implicating nociceptive afferent neurons as contributors to the reduction in vagal tone observed after MI.

**Fig. 4:**
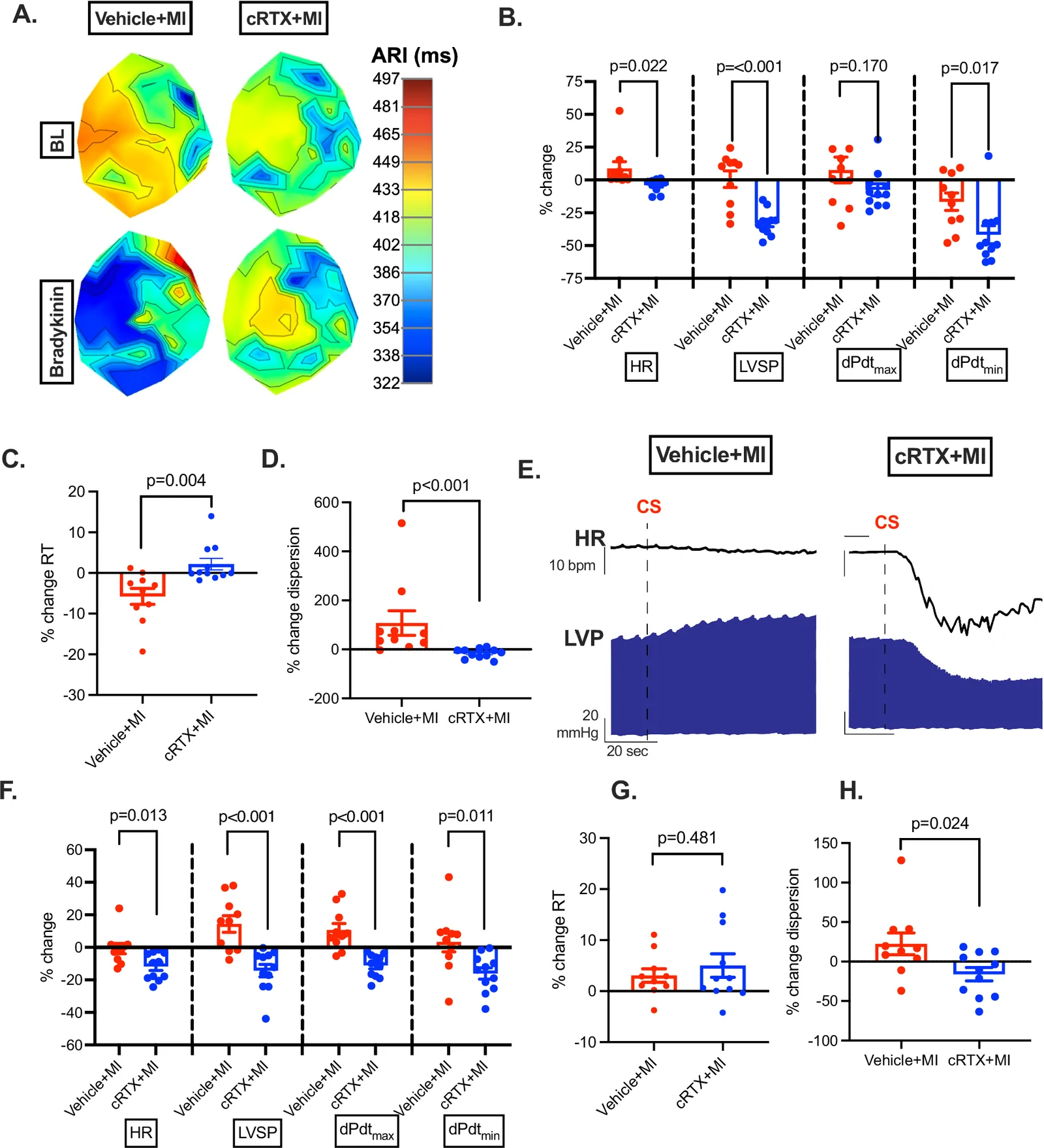
Effects of spinal afferent ablation with epidural RTX prior to MI on reflex-evoked cardiac function four-to-six weeks after MI. **A** Representative 2D-map of electrophysiological changes evoked by epicardial application of bradykinin in a vehicle (left) and an epidural cRTX (RTX-treated, infarcted) animal (right). **B**–**D** Quantified hemodynamic and electrophysiological responses to epicardial application of bradykinin in vehicle-treated (*n* = 10) and cRTX (*n* = 11) infarcted animals are shown. **E** Representation of hemodynamic changes evoked by epicardial application of capsaicin. **F**–**H** Quantified hemodynamic and electrophysiological changes in repolarization time (RT) and dispersion of RT upon epicardial application of capsaicin in vehicle (*n* = 10) and cRTX (*n* = 11) animals. Differences in heart rate (HR), left ventricular systolic pressure (LVSP), dP/dt_max_, dP/dt_min_, RT and dispersion responses upon bradykinin and capsaicin administration between vehicle and epidural cRTX animals were assessed using the two-sided unpaired Student’s *t*-test. CS: Capsaicin. Epidural vehicle-treated animals that subsequently were infarcted are denoted as Vehicle+MI and epidural RTX-treated animals that were subsequently infarcted are denoted as cRTX+MI. For all bar graphs, data are presented as mean values and error bars represent SEM. MI = myocardial infarction.

Lastly, we tested reflex-driven autonomic responses by the application of bradykinin and capsaicin to the ventricular epicardium to activate cardiac nociceptive afferent nerve endings. Epicardial bradykinin elicited a sympathoexcitatory response in vehicle-treated animals (*n* = 10), whereas cRTX animals (*n* = 11) exhibited a predominantly vagal response (decreased HR and LVSP; *p* < 0.05 for all; Fig. 4A, B). Because sympathetic stimulation can increase dispersion of repolarization (DoR)^50^, we further assessed ventricular electrophysiology and found that, in cRTX animals, bradykinin application prolonged repolarization time and reduced DoR (*p* < 0.05; Fig. 4C, D), consistent with improved electrophysiological stability.

Like bradykinin, capsaicin increased LVSP, LV inotropy, and DoR in vehicle-treated animals, consistent with a predominantly sympathetic and pro-arrhythmic response. However, in animals that had received epidural RTX prior to MI (cRTX animals), these effects were reversed at 4–6 weeks post-MI, with capsaicin evoking a vagal-predominant response with reduced observed DoR, suggestive of reduced arrhythmia susceptibility (Fig. 4E–H).

### RTX therapy prior to MI impedes post-MI oxidative stress, inflammation, and glial activation

**Fig. 5:**
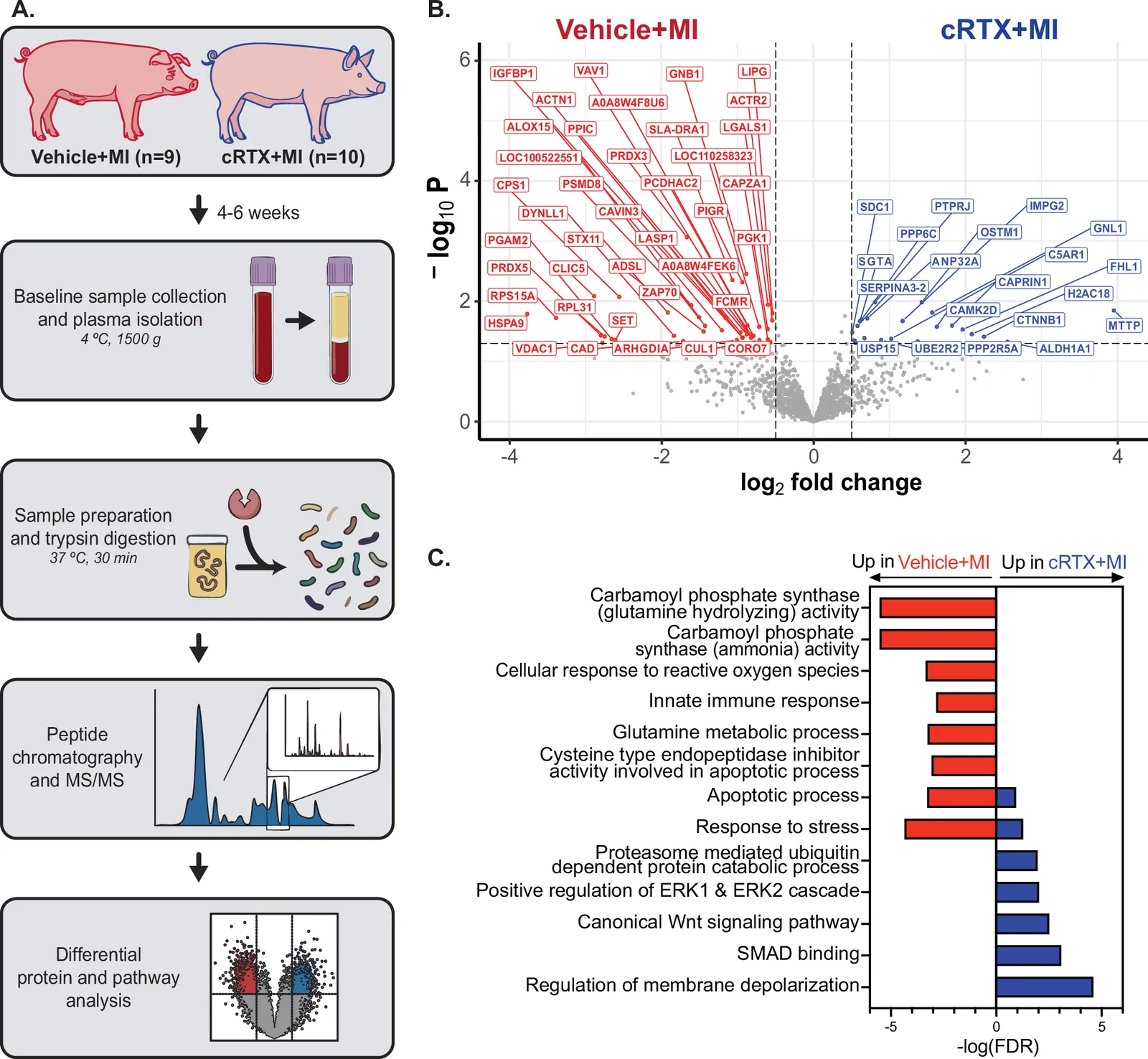
Proteomic analyses demonstrate a decrease in inflammatory and stress response pathways in infarcted animals with epidural RTX treatment prior to MI compared to vehicle-treated, infarcted animals four-to-six weeks post-infarction. **A** Schematic representation of plasma protein isolation to proteomic data analyses. **B** Volcano plot showing the differentially expressed proteins in vehicle and cRTX (resiniferatoxin-treated, infarcted) animals. **C** Proteomic pathway analyses conducted on expression data against the Gene Ontology database show a significant decrease in oxidative stress and inflammatory pathways in cRTX infarcted animals compared to vehicle-treated, infarcted animals. Pathways associated with extracellular signal-regulated kinase 1 (ERK1) and extracellular signal-regulated kinase 2 (ERK2) cascades were upregulated in cRTX animals, which have been described to be fundamental for cardiomyocyte homeostasis and stress responses. Differentially expressed proteins were identified using a two-sided Student’s *t*-test. Pathway analysis was performed against the Gene Ontology database using Rapid Integration of Term Annotation and Network. Animals that received vehicle (epidural saline) prior to MI are denoted as Vehicle+MI and animals that received epidural RTX prior to MI are denoted as cRTX+MI; *n* = 9 for Vehicle+MI, *n* = 10 for cRTX+MI. MS = mass spectrometry. Source data are provided with this paper.

Cardiovascular disease is characterized by systemic inflammatory responses and oxidative stress^51–53^. After MI, heightened sympathetic outflow amplifies inflammatory tone by noradrenergic signaling to hematopoietic niches, mobilizing myeloid cells into the circulation^54^. Thus, to assess if spinal nociceptive afferent signaling may also contribute to this post-MI inflammatory phenotype, we assayed differences in the plasma proteome by ELISA (Supplementary Fig. 6) and mass spectrometry of cRTX (*n* = 10) and vehicle-treated (*n* = 9) animals (Fig. 5A). Differentially expressed proteins in epidural cRTX versus vehicle animals mapped chiefly to immune response, oxidative stress, and cell cycle pathways (Fig. 5B, and Supplementary data 1). Notably, PSMD8, ZAP70 and VAV1, key immune regulators^55–57^, were downregulated 4-to-6 weeks after MI in cRTX animals. Moreover, pathways associated with immune activation and oxidative stress were also diminished in cRTX animals (Fig. 5C, and Supplementary data 2). Conversely, the ERK1/2 cascade, which has been reported to be fundamental for cardiomyocyte homeostasis^58^, was significantly increased in cRTX animals. Mean plasma levels of TNF-α at 4–6 weeks post-MI were significantly reduced in cRTX animals, while mean IL-12 plasma levels, although different, did not reach statistical significance (*p* = 0.052, Supplementary Fig. 6). Levels of IL-6 and IL-10 were below detection for both groups.

**Fig. 6:**
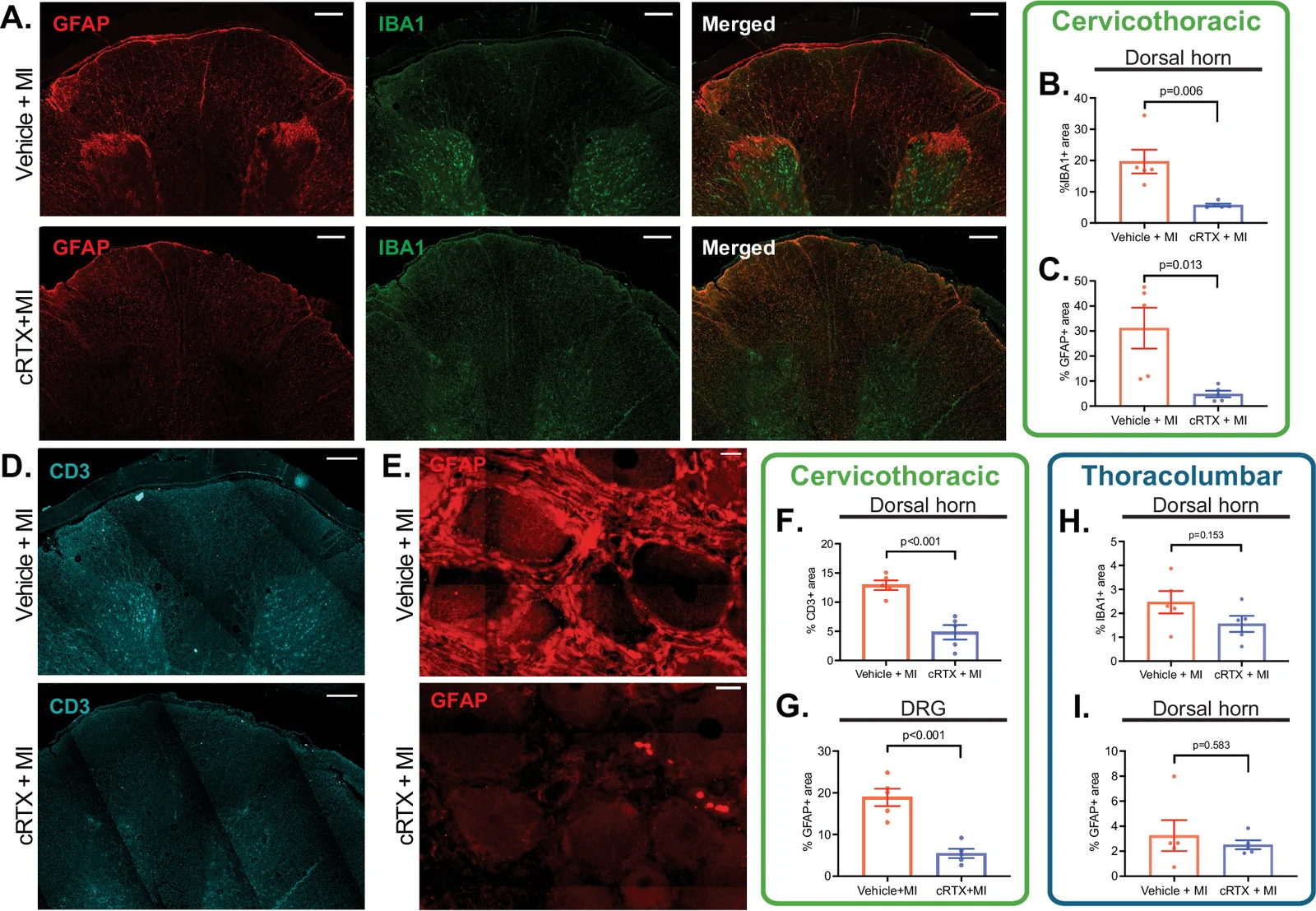
Immunohistochemical changes associated with ablation of cervicothoracic spinal nociceptive nerves with epidural RTX. **A** Representative images of immunostaining of spinal horns in cRTX and vehicle animals for glial fibrillary acidic protein (GFAP) and ionized calcium-binding adaptor molecule 1 (IBA1; markers of glial activation and microglia, respectively (scale bars represent 500 µm). Quantified data show decreased IBA1 (**B**) and GFAP (**C**) immunoreactivity in RTX-treated (cRTX; *n* = 5) *vs* vehicle-treated (*n* = 5) infarcted animals. **D** Representative images of immunostaining of the spinal horn for CD3 (marker of T lymphocytes). **E** Representative image of immunohistochemical staining of GFAP in T1 (thoraic level 1) dorsal root ganglia (scale bars represent 20 µm). **F** Quantified data show a significant reduction in the amount of CD3 positive T lymphocytes in cRTX (*n* = 5) *vs* vehicle-treated (*n* = 5) infarcted animals, suggesting reduced inflammation. **G** Quantified data showed a significant decrease in glial activation in the high thoracic dorsal root ganglia of cRTX (*n* = 5) versus vehicle (*n* = 5) animals. These effects of epidural RTX administration on reducing inflammation and glial activation were localized to the high thoracic region, as spinal cord dorsal horn expression of **H** IBA1, and **I** GFAP in the thoracolumbar region were similar between the vehicle-treated (*n* = 5) versus cRTX (*n* = 5). Differences between vehicle- and cRTX animals were assessed using the two-sided unpaired *t*-test. Animals that received vehicle (epidural saline) prior to MI are denoted as Vehicle+MI, and animals that received epidural RTX prior to MI are denoted as cRTX+MI. For all bar graphs, data are presented as mean values and error bars represent SEM. Source data are provided with this paper.

Given these effects on the systemic inflammatory response, we next asked whether spinal afferent signaling contributes to local spinal neuroinflammation and glial activation in the chronic post-MI period. To this end, we also quantified immunoreactivity for activated satellite glial cells (GFAP), microglia (IBA1), and T-cells (CD3) in the C7 and T1 dorsal horns of cRTX (*n* = 5) versus vehicle (*n* = 5). cRTX, infarcted animals showed reductions in GFAP, IBA1, and CD3 versus vehicle-treated controls (Fig. 6A–G).

Finally, we evaluated whether GFAP and IBA1 immunoreactivity were different at the level of the T12-L1 spinal horns. There was no difference in GFAP-IR or IBA1-IR in cRTX (*n* = 5) versus vehicle-treated (*n* = 5) infarcted animals in the thoracolumbar dorsal horns (Fig. 6H, I). Notably, vehicle-treated animals showed decreased GFAP and IBA1 in the thoracolumbar region as compared to their cervicothoracic region, suggesting that increased neuroinflammation in the cervicothoracic region is driven by the visceral organ affected (i.e., the heart), consistent with prior literature showing increased neuronal cFOS in the spinal cord limited to upper thoracic regions after MI^59^.

### Effects of RTX prior to MI on scar size, ventricular electrophysiology and arrhythmias

Scar size and detailed electrophysiological measurements were evaluated in RTX (*n* = 11) versus vehicle treated infarcted animals (*n* = 13) to determine if epidural RTX prior to MI had altered these after chronic MI. Four-to-six weeks after MI, the size of ventricular scars were not different between those that had received RTX versus vehicle prior to MI (Supplementary Fig. 7), as assessed by epicardial LV and RV voltage mapping and quantification of low voltage areas, defined as border zone and scar regions (percent low voltage area: 23.7 ± 2.3% in cRTX (*n* = 11) *vs* 22.3 ± 1.9% in vehicle (*n* = 13) animals, *p* = 0.634) and gross pathological examination and image quantification of dense scar regions of explanted hearts (scar size: 10.0 ± 1.3 % *vs* 10.7 ± 1.3% of anterior ventricular surface in cRTX (*n* = 11) *vs* vehicle (*n* = 13) animals, respectively, *p* = 0.690). In addition, analysis of echocardiographic data showed that LV end-systolic and end-diastolic areas were not significantly different between the two groups, with a similar change in these dimensions (quantified as LV ejection fraction or % change in systole) of 40.0 ± 2.8% *vs* 36.0 ± 3.8% in cRTX (*n* = 5) *vs* vehicle-treated (*n* = 5) animals, respectively (*p* = 0.540).

**Fig. 7:**
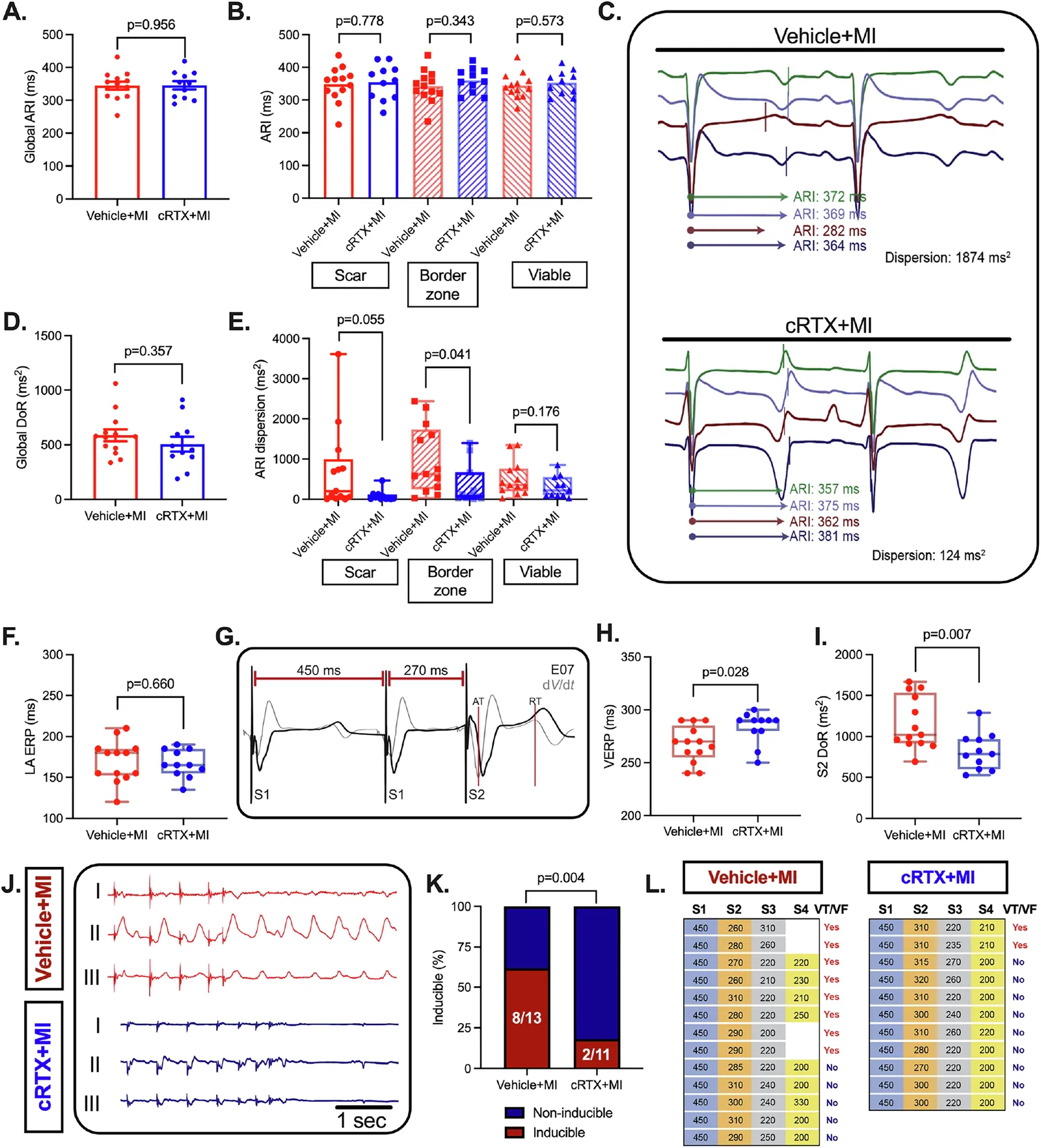
Effects of spinal nociceptive afferent ablation prior to MI on ventricular APD, DoR, refractoriness, and ventricular arrhythmia inducibility post-infarction. **A** Administration of cervicothoracic epidural RTX prior to MI did not alter global baseline activation recovery intervals (ARI), or **B** regional ARIs four-to-six weeks post-MI. **C** Examples of border zone electrograms from a vehicle (top) and RTX treated (cRTX+MI, bottom) animal showing ARIs from these regions. **D** In cRTX animals, global dispersion of repolarization (DoR) was not significantly different from vehicle-treated infarcted animals, however, **E** regional analysies demonstrated that ARI dispersion was greatest in border zone regions in both vehicle and RTX treated animals, but that this border zone dispersion was significantly less in infarcted animals treated with RTX. **F** Left atrial ERP (LA ERP) was not significantly different between vehicle-treated and cRTX animals. **G** Example of ventricular ERP (VERP) measurements showing the last-captured extra-stimulus (S2) at interval of 270 ms. **H** Ventricular ERP (VERP) was significantly prolonged in RTX treated animals, and **I** RT-dispersion of the S2 paced beat was significantly decreased in cRTX animals. **J** Example of VT/VF induction using programmed stimulation. The epidural vehicle-treated MI animal (upper panel) was inducible with 1 extra-stimulus, but the cRTX animal (lower panel) was not inducible. **K** VT/VF inducibility was reduced from 62% in vehicle-treated infarcted animals to 18% in cRTX animals with up to four extra-stimuli. **L** Breakdown of stimulation parameters used for induction of VT/VF. All electrophysiological parameters were compared between vehicle and cRTX animals using the two-sided unpaired Student’s *t*-test, VT inducibility was compared by the two-sided exact binomial test. *n* = 13 for vehicle and *n* = 11 for epidural cRTX for all reported electrophysiological parameters. Animals that received vehicle (epidural saline) prior to MI are denoted as Vehicle+MI, and animals that received epidural RTX prior to MI are denoted as cRTX+MI. For all bar graphs, data are presented as mean values and error bars represent SEM. For each boxplot, the center line indicates the median, the upper and lower bounds represent the first and third quartiles, and the whiskers represent the minimum and maximum values observed. Source data are provided with this paper.

For regional electrophysiological analysis, ARIs were compared between scar, border zone, and viable regions, with electrodes selected from each area based on epicardial voltage mapping^49,60^. Baseline (resting) global ventricular ARIs and regional ARIs were not significantly different between infarcted epidural cRTX-versus vehicle-treated animals (Fig. 7A–C). Moreover, basal ventricular global DoR was not significantly different between these groups (Fig. 7D). However, granular regional analyses demonstrated that the border zone dispersion was significantly greater in vehicle vs. cRTX animals (984 ± 234 *vs* 351 ± 154 ms^2^, respectively, *p* < 0.05; Fig. 7E). While atrial effective refractory period (ERP) was not significantly different (Fig. 7F), ventricular endocardial ERP (measured at the right ventricular apex) was significantly longer in cRTX versus vehicle animals (283 ± 5 *vs* 267 ± 5  ms, respectively, *p* < 0.05, Fig. 7G, H), suggesting increased ventricular refractoriness due to ablation of spinal nociceptive neurons/fibers. Finally, DoR of the prematurely paced beat (S2), which is known to activate spinal receptors and reflexively increase sympathetic outflow resulting in ventricular arrhythmias^61,62^, was significantly less in epidural cRTX (cRTX: 817 ± 68 ms^2^) *vs* vehicle animals (1163 ± 90 ms^2^, *p* < 0.05, Fig. 7I). Importantly, cRTX animals were less inducible for VT/VF with extra-stimulus pacing (18% in cRTX *vs* 62% in vehicle, *p* < 0.05; Fig. 7J–L), while there was no difference in pacing thresholds to capture the ventricular myocardium (Supplementary Fig. 8).

### RTX administration after established infarction acutely rescues arrhythmogenic phenotype

Having shown that administration of epidural RTX and ablation of cervicothoracic nociceptive neurons prior to MI (cRTX) impeded autonomic/electrophysiological remodeling and reduced VT/VF inducibility at 4–6 weeks post-MI, we next asked whether RTX could still be therapeutic benefit after pathological remodeling has been established. To test this hypothesis, a separate group of pigs underwent myocardial infarcts, and 4–6 weeks later, received cervicothoracic epidural RTX (aRTX, *n* = 15); hemodynamic and autonomic responses were assessed acutely every hour up to 4 h after RTX administration.

**Fig. 8:**
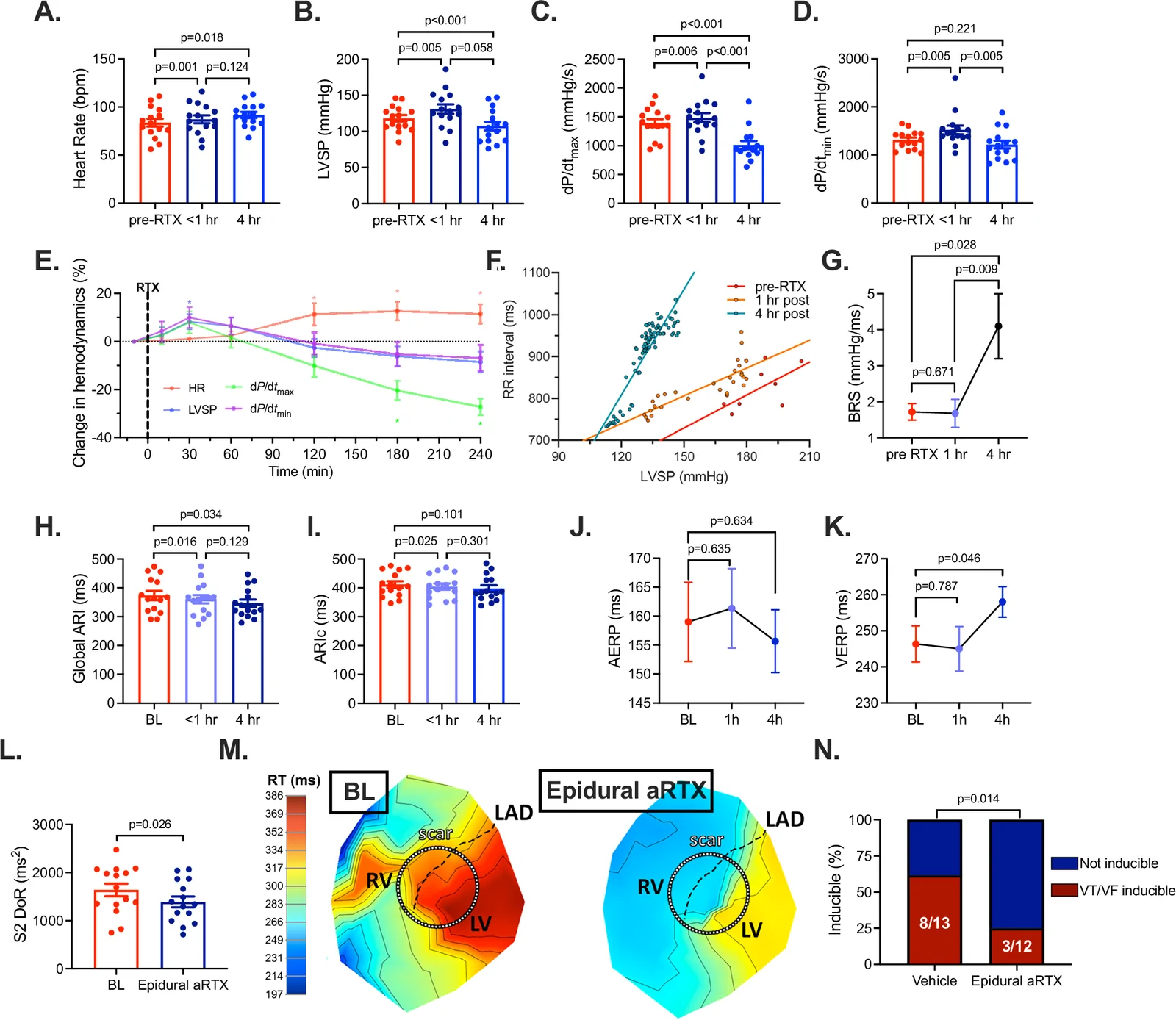
Temporal hemodynamic responses and electrophysiological effects of acute cervicothoracic spinal nociceptive afferent ablation with epidural RTX in the setting of chronic MI. The acute effects of epidural RTX (aRTX) were assessed to determine the kinetics and tolerability of RTX in infarcted animals (*n* = 15). Within 1-h of administration, RTX increased **A** heart rate (HR), **B** left ventricular systolic pressure (LVSP), **C** left ventricular inotropy (dP/dt_max_), and **D** left ventricular lusitropy (dP/dt_min_). Left ventricular (LV) lusitropy remained significantly elevated, whereas LV inotropy and LVSP were decreased 4 h after epidural RTX administration. **E** Percentage change in HR, LVSP, LV inotropy and lusitropy over time are shown. ^*^p < 0.05 vs baseline. **F** Beat-to-beat changes in RR interval vs LVSP were plotted to assess baroreflex sensitivity (BRS) before, 1-h, and 4-h post-RTX injection. **G** BRS was significantly improved by 4-h post-RTX (*n* = 10). **H** Acute epidural RTX shortened activation recovery intervals (ARI) at 1- and 4-h after administration. **I** When corrected for heart rate (ARIc), this effect was no longer significant. **J** Epidural RTX did not alter atrial ERP (AERP) in chronic MI animals. **K** Epidural RTX prolonged VERP 4-h after administration. **L**, **M** This was accompanied by a reduction in the S2 dispersion of repolarization time (DoR). **N** While 8/13 vehicle animals were inducible for sustained VT/VF, only 3/12 epidural aRTX animals were inducible at 4 h post-RTX administration. Serial hemodynamic and electrophysiological parameters were compared by repeated-measures ANOVA. Pre-RTX vs. <1 h or 4 h and 1 h vs. 4 h comparisons were performed only if repeated measure ANOVA was statistically significant and compared using two sided paired Student’s *t*-test (uncorrected). Serial BRS was compared using the Friedman test (uncorrected) and RT dispersion was compared using a two-sided paired Student’s *t*-test. Inducibility was compared using the two-sided exact binomial test. BL baseline, RV right ventricle, LV left ventricle, LAD Left anterior descending artery. For all bar graphs, data are presented as mean values and error bars represent SEM.

Consistent with animals receiving RTX prior to MI (cRTX), aRTX induced an initial sympathoexcitatory response with peak increases in HR (84 ± 4 to 87 ± 4 beats/min, *p* = 0.001), LVSP (118 ± 5 to 131 ± 7 mmHg, *p* < 0.01), and inotropy (1393 ± 67 to 1484 ± 79 mmHg/s, *p* < 0.01) within the first hour, Fig. 8. By 4 h after epidural RTX administration, HR had stabilized but remained higher than pre-RTX values, while LV inotropy and LVSP were slightly lower (Fig. 8A–E).

While hemodynamic parameters peaked within the first hour after RTX, no effect on vagal function, as assessed by BRS, was observed at 1 h (from 1.8 ± 0.2 pre-RTX to 1.7 ± 0.4 ms/mmHg 1-h post-RTX, *p* > 0.05; *n* = 10, Fig. 8F, G). By 4  h after aRTX, however, BRS had significantly increased to 3.9 ± 0.7 ms/mmHg (*p* < 0.05), suggestive of augmented vagal function (Fig. 8F, G).

While previous studies have acutely demonstrated the clinical anti-arrhythmic efficacy of thoracic epidural anesthesia using lidocaine or bupivacaine^63,64^, the differential contributions of efferent versus afferent blockade to ventricular arrhythmias remain unclear. Furthermore, whether acute spinal afferent blockade alone could be anti-arrhythmic is unknown. Hence, the effects of spinal sympathetic afferent nociceptive ablation on cardiac electrical stability were evaluated before and 4 h after epidural RTX administration in chronically infarcted animals. After epidural aRTX, global ventricular ARIs significantly shortened (374 ± 16 pre-RTX to 345 ± 14 ms post-RTX; *p* < 0.05, Fig. 8H), whereas heart rate-corrected ARI (ARIc) was unchanged, indicating a HR-dependent effect, Fig. 8I. Atrial ERP remained unchanged after aRTX (*p* > 0.05; Fig. 8J), but right ventricular endocardial ERP significantly increased from 246 ± 5 pre-RTX to 258 ± 4 ms 4 h post-RTX (*p* < 0.05), Fig. 8K. Moreover, while epidural aRTX did not alter DoR during sinus rhythm, it significantly decreased DoR of the ventricular extra-stimulus (S2) paced beat (1639 ± 130 pre-RTX *vs* 1389 ± 112 ms^2^ post-RTX, *p* < 0.05, Fig. 8L, M). No animals developed spontaneous ventricular arrhythmia following administration of epidural RTX, despite the initial sympathoexcitatory response and presence of a chronic infarct. However, at 4 h post-RTX administration, only 3 of 12 infarcted animals were inducible for VT/VF, significantly fewer than the chronic infarcted animals who had only been treated with vehicle (25% *vs* 62%, respectively; *p* < 0.05; Fig. 8N).

## Discussion

**Fig. 9:**
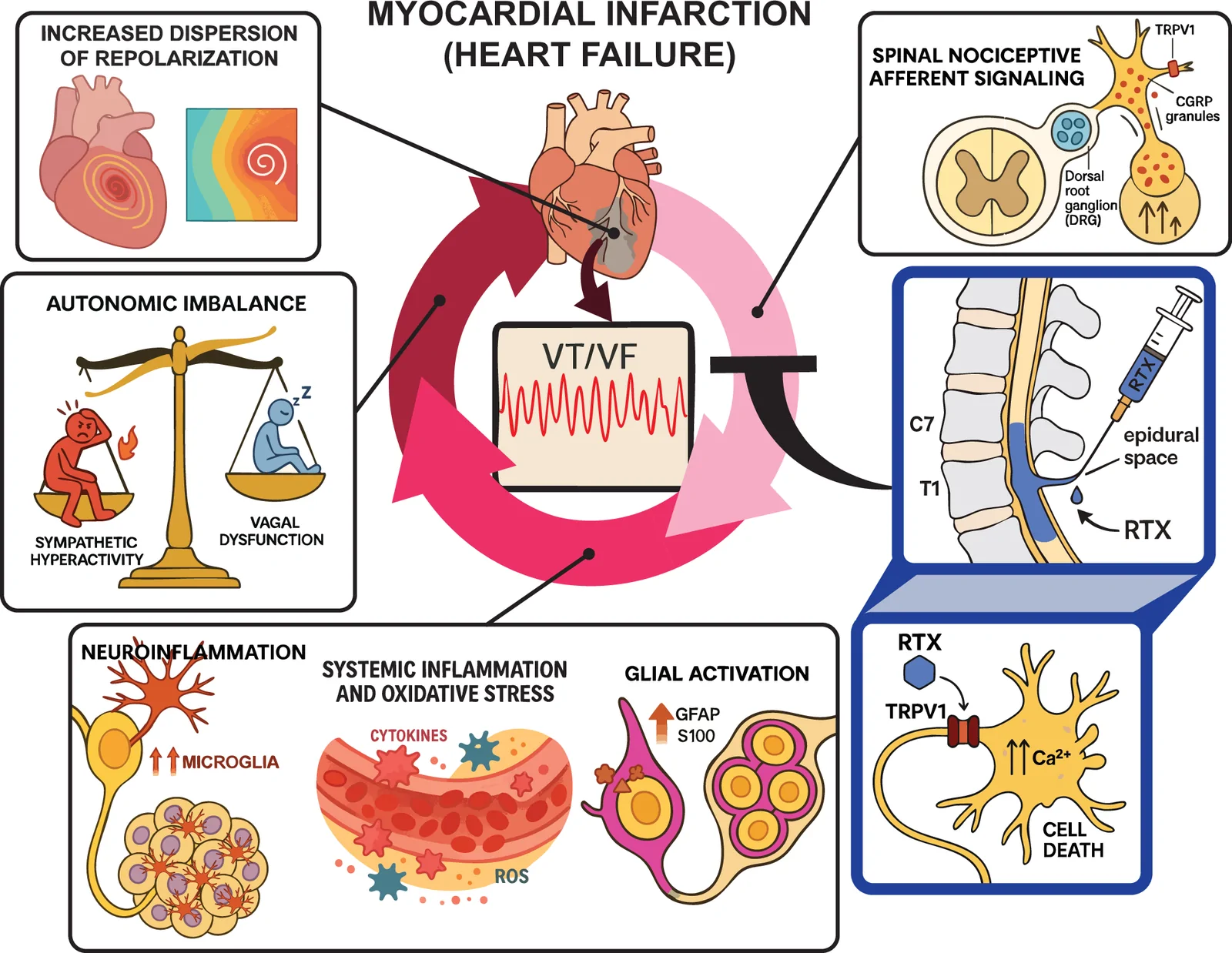
Spinal nociceptive afferent signaling contributes to post-myocardial infarction neuroinflammation, oxidative stress, glial activation, and sympatho-vagal imbalance, resulting in increased dispersion of repolarization and ventricular arrhythmias (VT/VF), a process that is mitigated by interruption of this signaling with epidural resiniferatoxin (RTX). GFAP glial fibrillary acid protein, TRPV1 transient receptor potential vanilloid 1, CGRP calcitonin gene-related peptide.

The present study demonstrates that cervicothoracic spinal nociceptive afferent signaling is a critical factor in initiating and sustaining the chronic pathological autonomic remodeling after MI (Fig. 9). Given the multifactorial etiology of ventricular arrhythmias, we elected to dissect the role of spinal nociceptive afferent signaling on autonomic, inflammatory, and electrophysiological mechanisms underlying the increased risk of ventricular arrhythmias after chronic MI. Local interruption of spinal TRPV1 afferent signaling after MI impeded the development of vagal dysfunction, suppressed neuroinflammation and glial activation, attenuated systemic inflammation and oxidative stress, and stabilized cardiac electrophysiological parameters, reducing VT/VF inducibility. These results highlight the potentially critical role of spinal TRPV1-expressing afferents in the development of the multitude of chronic pathological autonomic remodeling processes that have been described after MI^1–3,52,65–68^ and provide a potential target for ameliorating these effects. Importantly, even when delivered after established MI and pathological remodeling (4–6 weeks post-MI), RTX-mediated ablation was sufficient to enhance vagal tone and mitigate electrophysiological instability, corresponding to fewer inducible ventricular arrhythmias. Collectively, these findings highlight the role of spinal afferents in contributing to the development of autonomic remodeling after MI as well as support their role in sustaining autonomic dysfunction and pro-arrhythmia.

These autonomic changes merit closer consideration. After MI, not only does sympathetic neural remodeling occur, but vagal withdrawal is also observed, as reflected in decreased baroreflex sensitivity, heart rate variability, and altered activity of post-ganglionic parasympathetic neurons^1–3,52,65–68^, ultimately contributing to VT/VF. Reduced vagal function, as assessed by BRS, is an independent predictor of SCD and VT/VF in patients with MI and heart failure^40,42^. Persistent chronic cervicothoracic spinal afferent signaling after MI results in increased efferent sympathetic outflow. However, it has also been reported to contribute to vagal dysfunction, as suggested by experiments where activation of these fibers resulted in reduced BRS and, conversely, improvements in BRS and cardiac post-ganglionic vagal neuronal activity are observed with thoracic epidural anesthesia^10,48,69^.

In the present study, ablation of nociceptive TRPV1-expressing neurons prior to MI (cRTX) preserved vagal function 4-to-6 weeks after MI. Moreover, responses to epicardial application of either the direct TRPV1 agonist capsaicin^70^ or the B2 receptor ligand bradykinin, which sensitizes TRPV1+ neurons at the time of myocardial ischemia^71^, were blunted in RTX-treated MI animals. In fact, while vehicle-treated infarcted animals exhibited an overt sympathoexcitatory response, cRTX animals exhibited a predominantly vagal response, accompanied by reductions in DoR, consistent with greater electrical stability. Following MI, denervation and heterogeneous nerve sprouting at scar/border zone regions is reported^5,68,72,73^, further amplifying electrical heterogeneity during sympathetic activation, increasing DoR and predisposing to VT/VF^74–77^. The decrease in DoR is also consistent with the potentially cardioprotective effects of vagal TRPV1-positive afferents^78,79^ that do not pass through the DRG or the epidural space, and therefore, remain intact.

These functional changes are paralleled by remodeling at the cellular level. MI and heart failure with reduced ejection fraction have been shown to induce satellite glial cell activation in the peripheral autonomic ganglia, spinal cord, and autonomic regions of the brain^3,65,80–85^. Satellite glial cells have been reported to respond to increased neuronal activity but may themselves augment neuronal synaptic transmissions^86^, potentially sustaining increased sympathetic reflexes. Importantly, the interplay of these cells has been shown in the stellate ganglia to establish a feed-forward process which can enhance sympathetic activity^87^. In the present study, 4-to-6 weeks after MI, we observed diminished levels of glial activation in both the spinal cord and DRG of animals treated with RTX prior to MI. However, as satellite glial cells do not express TRPV1, this decrease may be secondary to diminished neuronal afferent activation^88,89^. The concomitant attenuation of afferent signaling and local satellite glial cell activation may cumulatively blunt efferent neuronal activation and reflex activity through the spinal cord^86^, thereby improving sympathovagal balance. Taken together, preservation of parasympathetic tone, coupled with reduced molecular and cellular markers of autonomic remodeling in the peripheral ganglia of RTX-treated animals, supports the hypothesis that spinal nociceptive afferents play a critical role in the pathological autonomic remodeling observed in the setting of chronic MI.

Beyond these autonomic effects, MI is associated with systemic inflammation, oxidative stress^52,53,80,90,91^, and neuroinflammation^92,93^. Elevated inflammatory cytokines^94–99^ and increased oxidative stress^100–104^ are associated with poorer outcomes and an increased risk for VT/VF in patients with MI and heart failure. However, clinical trials of anti-inflammatory therapies (e.g., TNF-α, IL-1β, and IL-6 inhibitors) have yielded mixed results in this patient population^105–107^. In this study, plasma proteomics indicated lower markers of inflammation and stress in cRTX versus vehicle-treated animals. In parallel, we also observed reductions in microglial and T lymphocyte markers in the cervicothoracic dorsal horn of epidural RTX-treated animals, suggesting that RTX treatment attenuated chronic neuroinflammation. It is thus possible that spinal nociceptive afferent signaling may contribute to the heightened neuroinflammatory milieu observed after MI. Furthermore, beyond reducing local neuroinflammation, the improved vagal tone after RTX treatment may also engage the splenic cholinergic anti-inflammatory reflex^108^, where acetylcholine binds to nicotinic receptors on white blood cells, to attenuate systemic cytokine release and systemic inflammation^109–111^.

These inflammatory and autonomic processes contribute to an electrophysiological substrate that is susceptible to arrhythmias. Acute MI causes myocardial injury, axonal denervation, and localized nerve sprouting^67,112^. In the setting of sympathoexcitation, these changes culminate in electrical heterogeneity, especially in border zone regions, serving as the substrate for VT/VF^8,113,114^. In the present study, treatment with cervicothoracic epidural RTX prior to MI (cRTX) led to a reduction in DoR (i.e., electrical heterogeneity) in the border zone myocardium 4-to-6 weeks post-MI. Border zone regions, due to their innervation and myocardial heterogeneity, serve as sites for both the trigger and substrate of VT/VF^115–117^. Interestingly, the reductions in VT/VF-inducibility in RTX-treated animals were comparable to the anti-arrhythmic benefits reported with thoracic epidural anesthesia using bupivacaine^63^ and cardiac sympathetic denervation in animal models post-MI and in patients with refractory VT/VF^118,119^, interventions that simultaneously interrupt all spinal afferent *and* efferent sympathetic signaling. Given that RTX selectively targets nociceptive afferents in particular, the similar magnitude of anti-arrhythmic effects raises the possibility that a substantial portion of thoracic epidural anesthesia’s benefit is derived specifically from the blockade of these fibers.

Having observed  the above effects when spinal nociceptive ablation preceded infarction, we next considered whether spinal afferent signaling also contributes to VT/VF, once chronic scar and autonomic remodeling are already established. A prior study in pigs without a myocardial infarction assessed the effects of T2-T4 DRG RTX application 30 min prior to induction of ischemia on VT/VF burden during the acute ischemia and 1 h reperfusion period^29^. In light of that report and the observed longitudinal effects of RTX prior to MI reported herein, we subsequently aimed to evaluate the effects of C7-T1 afferent ablation acutely 4-to-6 weeks after MI. Acute epidural RTX administration in chronically infarcted pigs was well tolerated, with no observed hemodynamic instability. Within 4 h of administration, we observed improvements in vagal BRS, increased ventricular refractory period, attenuated pacing-induced DoR, and lower VT/VF inducibility. Taken together, these data suggest that, in the setting of chronic MI, cervicothoracic nociceptive spinal afferents, perhaps by increasing efferent outflow, are key contributors to the maintenance of pathological vagal dysfunction and cardiac electrical instability, thereby supporting their role in VT/VF susceptibility.

These findings carry important clinical implications. Given the selectivity of RTX for nociceptive TRPV1-expressing neurons, it is currently under investigation in clinical trials for chronic and refractory pain syndromes (NCT00804154; NCT02522611), highlighting its translational potential. In addition to showing that nociceptive cervicothoracic spinal afferents are critical to the pathogenesis and maintenance of autonomic remodeling in the setting of chronic MI, our data support the clinical potential of acute and chronic, selective, spinal afferent ablation after MI. Importantly, while epidural RTX was well tolerated hemodynamically in both healthy and infarcted animals, initial administration of RTX was associated with a transient sympatho-excitatory phase that peaked within 1 h. In our cohort of animals, where RTX was delivered prior to MI, this was accompanied by increased incidence of ventricular arrhythmias in the early MI convalescent period (~2 h post-RTX).

Overall, the present study supports selective spinal ablation of TRPV1-expressing nociceptive afferents as a strategy to improve sympatho-vagal imbalance and acutely reduce VT/VF inducibility in chronically infarcted hearts. These findings are in line with prior work demonstrating the acute anti-arrhythmic effects of direct RTX application on thoracic DRGs in a porcine model of acute ischemia^29^, and extend these observations by demonstrating the important role of spinal afferents signaling in the subsequent development of post-MI remodeling. Future studies are warranted to further determine the durability of, and extent to which, cervicothoracic epidural RTX late after chronic MI can *reverse* or early after MI, *prevent*, pathological cardiac autonomic remodeling. As mentioned above, the reductions in VT/VF-inducibility in chronic RTX-treated animals were comparable to the anti-arrhythmic benefits reported with thoracic epidural anesthesia using bupivacaine^63^ and cardiac sympathetic denervation in patients with refractory VT/VF and electrical storm^118,119^. Advantages of epidural RTX over these therapies would be its non-surgical approach and the specificity of its target, as it leaves both mechanosensory afferents (in contrast to thoracic epidural anesthesia) as well as cardiomotor efferents intact.

Several limitations should be acknowledged. Although the initial sympathoexcitatory responses RTX and post-mortem histology supported successful local TRPV1 nociceptive afferent ablation, acute confirmation of ablation in vivo was not possible. In addition, given epidural administration at C7-T1 vertebral levels, effects of RTX in this study may represent an underestimate of its potential efficacy, as complete blockade of *all* cervicothoracic spinal afferent nerves was difficult to confirm. However, the blockade herein was sufficient to mitigate VT/VF inducibility and reduce post-MI autonomic remodeling.

While RTX was administered at the C7-T1 levels only, effects on other organ systems cannot be completely ruled out. Nevertheless, as renal^120–122^, splenic^123^, and hepatic^124–126^ spinal afferents originate from low thoracic/lumbar levels, and prior work indicates limited spread of epidurally-administered compounds^36–38^ (including epidural RTX), it is unlikely that these organs were affected significantly by RTX administered at the cervicothoracic level. Consistent with this, plasma aspartate transaminase (AST) and alanine aminotransferase (ALT), clinical indices of liver health, were similar between cRTX and vehicle-treated animals. Although pulmonary spinal afferents run through similar thoracic levels as cardiac spinal afferents, pulmonary innervation by spinal TRPV1-expressing nociceptive neurons is sparse^127^, and largely confined to large airways. Furthermore, activation of these pulmonary afferents has been shown to result in large airway smooth muscle relaxation^128–134^, and therefore, is unlikely to account for the cardiac electrophysiologic and autonomic results observed in this study. More distally, we did not observe effects on the T12-L1 spinal cord/DRG that innervate the kidneys, consistent with renal-independent benefits of RTX. Nevertheless, as assessment of CGRP innervation of pulmonary, splenic, renal or hepatic tissue was not performed in this study, and potential off-target effects or benefit, which may have contributed to the results observed, cannot be completely ruled out. In addition, inflammatory-mediated cardiac remodeling was not directly characterized in this study. As such, the improved autonomic tone and electrical stability observed with RTX treatment may reflect a combination of direct neurally-mediated and indirect anti-inflammatory benefit.

Finally, in this study, CGRP was used as a surrogate for TRPV1-expressing nociceptive neurons/fibers, as direct TRPV1 immunolabeling was unreliable in our hands. However, activation of TRPV1 has been reliably shown to evoke the release of neurotransmitters such as CGRP^135–141^. Therefore, CGRP has been widely used as a marker for TRPV1-expressing afferent neurons.

In conclusion, cervicothoracic spinal/sympathetic nociceptive afferent signaling plays a significant role in the occurrence of the multitude of reported pathological autonomic changes after chronic MI. In chronically infarcted animals, ablation of C7-T1 spinal afferents prior to MI was associated with preserved vagal function, reduced glial activation and inflammatory signaling, and improved electrophysiological stability at 4 to 6 weeks post-MI. These changes coincided with lower DoR in border zone regions, areas known to serve as  both the trigger and the substrate for VT/VF, and ultimately reduced VT/VF inducibility. The anti-arrhythmic benefits observed with epidural RTX in the chronic MI setting may therefore reflect a combination of dampened inflammatory/stress pathways together with preservation of parasympathetic tone and vagal reflexes, limiting electrical heterogeneity.

Additionally, in a cohort of animals where RTX was administered instead 4–6 weeks following myocardial infarction, acute cervicothoracic epidural RTX was associated with improved vagal function and reduced VT/VF inducibility, suggesting potential utility for patients presenting with refractory VT/VF in the setting of a prior MI. Collectively, these findings offer important insights into the mechanisms underlying the chronic pathological autonomic remodeling and susceptibility to ventricular arrhythmias after MI.

## Methods

### Ethical approval

Animal care was performed according to the National Institutes of Health Guide for the Care and Use of Laboratory Animals. All experimental protocols were approved by the UCLA Institutional Animal Care and Use Committee. All animals had an acclimatization period of 7 days at the UCLA  animal facility, prior to undergoing any experiments. The temperature was regulated between 16 and 18 °C. Swine were housed alone or in pairs, and boxes were enriched with chains, balls, and additional playing materials. Pigs were exposed to dark–light cycles of 12 h with artificial lighting between 7 and 19 o’clock. Drinking water was supplied ad libitum, and pigs were fed regular chow (LabDiet Mini-Pig; Item #55894).

In total, 57 male Yorkshire pigs (S&S Farms) were included in the study. Of these, three were healthy animals (Control; *n* = 3) initially used to assess hemodynamic effects of acute epidural RTX. Of the 54 infarcted animals, 37 pigs survived the MI creation and the follow-up 4-to-6-week post-MI period to terminal studies. Nineteen animals were randomized to epidural RTX administration prior to MI (cRTX), of which 11 survived the acute MI and the 4-to-6-week period post-MI, and 18 animals were randomized to vehicle (underwent thoracic epidural catheter placement without RTX administration) prior to MI, of which 13 survived the acute MI and the 4-to-6-week period following MI. A fourth group of 17 animals underwent MI creation, of which 15 survived the acute MI and the 4-to-6-week period post-MI, and were used for evaluation of the effects of acute cervicothoracic epidural RTX in chronically remodeled infarcted animals, Fig. 1A. In cRTX- and vehicle-treated animals, terminal experiments were performed 4 to 6 weeks after epidural RTX injection and MI creation. Vehicle-treated animals had an epidural catheter placed and contrast/saline (vehicle) injected without RTX, 2–3 h prior to induction of MI, with terminal assessments also performed at 4-to-6 weeks post-MI and saline injections (Fig. 1).

On the other hand, in the aRTX cohort, chronic MI was induced, and animals were evaluated 4 to 6 weeks after MI. The epidural catheter was placed and RTX administered with the same approach. Hemodynamic and electrophysiological parameters were acutely assessed before and for the 4 h following administration of epidural RTX.

Not all animals underwent the same interventions at the time of terminal studies due to the prolonged length of experiments and potential for micro- or macro-dislodgment of neural recording electrodes from the VIVGP with cardioversion of VT/VF or rinsing with saline after epicardial bradykinin/capsaicin application. Hence, animal numbers are indicated with each intervention under each section of the results.

Animals were monitored throughout the study by laboratory staff under veterinary oversight. Humane endpoints followed institutional Animal Research Committee policy, consistent with IACUC guidelines, and included chronic weight loss exceeding 20% of baseline body weight, a body condition score below 2, a Grimace scale score above 2, uncontrollable seizures, incoordination or paralysis, respiratory distress, decreased mobility interfering with normal eating or drinking, weak response to external stimuli, or uncontrolled bleeding; no animals met these criteria. Terminal experiments were open-chest procedures, and euthanasia was performed by induction of ventricular fibrillation with low-voltage direct current while animals were maintained in a deep surgical plane of anesthesia under inhaled isoflurane (4–5%), with death confirmed by loss of heartbeat over a minimum of 10 min together with standard clinical criteria. Animals reaching a humane endpoint prior to the terminal study would have been euthanized with intravenous pentobarbital or pentobarbital solution (≥100 mg/kg), with death confirmed by cessation of breathing, loss of pulse, and absence of corneal reflex.

### Creation of myocardial infarcts

MI was created percutaneously under fluoroscopic guidance, as previously described^49^. Briefly, animals (40.4 ± 0.7 kg; 93 ± 13 days of age) were sedated with tiletamine-zolazepam (4-8 mg/kg, intramuscular), intubated, and placed under general anesthesia (isoflurane 1–2%, inhaled). Pre-operative analgesic care consisted of buprenorphine (0.02 mg/kg; intramuscular) and carprofen (4.4 mg/kg; intramuscular). A coronary guide wire was introduced via the femoral artery into the left anterior descending coronary artery (LAD), and a balloon-tipped angioplasty catheter was advanced over an angioplasty wire past the first diagonal branch of the LAD and placed at the second diagonal branch. Next, the balloon was inflated and 3–4 ml polystyrene microspheres (Polybead, 90 μm, Polysciences, Warrington, PA) were injected through the angioplasty balloon lumen into the distal LAD. The balloon was deflated, and MI was confirmed by lack of distal LAD flow on coronary angiography coupled with ST-segment changes (Fig. 1B, C). Post-operatively, animals were continuously monitored until they were well awake and eating, after which they were checked on every 12 h for the first 3 days, and every day after that. Post-operative analgesia was continued for 72 h post-experiment, and consisted of buprenorphine (0.02 mg/kg; intramuscular; twice a day) and carprofen (4.4 mg/kg; intramuscular; once a day). Animals were then survived for 4 to 6 weeks.

### Cervicothoracic (C7-T1) epidural nociceptive denervation with resiniferatoxin

Pigs were placed in the lateral decubitus position. Using a paramedian approach, a 17-gauge Tuohy needle was inserted at the T5-T6 vertebral level, and the epidural space was identified by standard loss-of-resistance under fluoroscopic guidance. Correct placement of the catheter in the epidural space was then confirmed by contrast injection under fluoroscopic guidance. A 19-gauge open-end epidural catheter (Teleflex Inc, Wayne, PA) was then advanced superiorly to the C7-T1 vertebral level (Fig. 1F) and contrast was again injected to confirm position. RTX or saline with contrast was injected into the epidural space. All cRTX animals (those receiving RTX prior to MI and studied 4-to-6 weeks later) received 1.2 µg/kg of RTX in 1 mL. In the chronic infarcted animals that received RTX 4 to 6 weeks after MI acutely, 7/15 received 1.2 µg/kg of RTX while 8/15 a lower dose of 0.6 µg/kg of RTX (also in 1 mL) to evaluate any differences in hemodynamic or electrophysiological responses. As no hemodynamic or electrophysiological differences were observed between the two groups (Supplementary Fig. 9), data were pooled for analyses involving the aRTX animals.

### Animal preparation

For terminal studies, animals (49.8 ± 0.1 kg; 127 ± 10 days of age) were sedated with tiletamine-zolazepam (4–8 mg/kg, intramuscular) and intubated. General anesthesia was induced with isoflurane (1–2%, inhaled) and transitioned to α-chloralose (50 mg/kg initial bolus, then 20–30 mg/kg/h. infusion) prior to autonomic or electrophysiological testing. Sheaths were placed in bilateral femoral veins and arteries for saline infusion, drug administration, pressure monitoring, and introduction of electrophysiological catheters.

### Intracardiac echocardiography

Prior to any intervention, an intracardiac echocardiography (ICE) probe (ViewFlex Xtra ICE Catheter, Abbott, Minneapolis, USA), connected to the ViewMate Multi Ultrasound System (Abbott, Minneapolis, USA), was introduced via the right femoral vein into the right atrium in 10 vehicle- and 10 RTX-treated (cRTX) animals. Upon placement in the right atrium, the probe was slowly retracted and rotated to obtain a view similar to the parasternal short axis view. Recordings of the LV were obtained at the level of the papillary muscles. Images were analyzed offline using Sante DICOM Viewer Lite (Santesoft Ltd, Nicosia, Cyprus) to obtain diastolic and systolic LV area and wall thickening measurements.

### Ventricular hemodynamic measurements

A 5-Fr Millar pressure-conductance catheter was introduced via the femoral artery and placed in the LV for continuous pressure. Raw signals were digitized by a CED Power1401 and analyzed using Spike2 (Cambridge Electronic Design, v10.08). A continuous 12-lead ECG was obtained via a CardioLab Recording System (GE Healthcare).

### Ventricular electrophysiological measurements

All animals underwent median sternotomy to expose the heart. A 56-electrode sock, connected to a GE CardioLab system, was placed over the ventricles for continuous local unipolar epicardial electrogram recordings (band pass filtered 0.05–500 Hz, Fig. 1D). Activation time (AT) and repolarization time (RT) were measured by customized software (iScaldyn; University of Utah) from these unipolar electrograms as the intervals from onset of ventricular activation to the minimal dV/dt of the depolarization wave-front or maximal dV/dt of the repolarization wavefront, respectively (Fig. 1E). Activation recovery interval (ARI), a surrogate for action potential duration^142^, was calculated as the difference between RT and AT, and corrected for differences in HR using the Bazett formula. Global dispersion in RT (DoR) was calculated as the RT variance across all sock electrodes, whereas regional DoR was calculated as the variance across electrodes assigned to regions based on bipolar voltage mapping (below).

Bipolar voltage mapping was performed in epidural cRTX (*n* = 11) and vehicle (*n* = 13) animals using a standard 2-2-2 duodecapolar catheter (Abbott, Minneapolis, USA) to delineate scar, border zone and viable regions. For this purpose, the duodecapolar catheter was advanced between the epicardium and the sock electrode, and the bipolar voltage underneath each respective electrode was measured. Using standard voltage criteria, regions were defined as either scar (0.05 mV <voltage < 0.5 mV), border zone (0.5 mV <voltage < 1.5 mV), or viable (voltage > 1.5 mV) myocardium^60^.

### Evaluation of cardiac autonomic function

#### Baroreflex sensitivity

Vagal baroreflex sensitivity was tested in vehicle-treated animals (epidural with saline/contrast only followed by MI; *n* = 13), cRTX (epidural with RTX followed by MI with evaluation 4-to-6 weeks post-MI, *n* = 11), aRTX (MI followed by epidural RTX 4 to 6 weeks later; *n* = 12) by bolus injection of phenylephrine (3–5 μg/kg, IV) to evoke a 30–40 mmHg increase in systolic pressure. Vagal BRS was measured hourly for 4 h in aRTX animals, while it was measured once in control, vehicle and cRTX animals. The slope of the linear regression describing the beat-to-beat relationship between RR-interval and LV systolic pressure (LVSP) was used to quantify baroreflex sensitivity (BRS)^143^.

#### Cardiac nociceptive stimulation

Epicardial application of bradykinin (1.06 mg/mL) and capsaicin (0.03 mg/mL) in vehicle (*n* = 10) and cRTX (*n* = 11) animals was used to characterize autonomic responses to epicardial stimulation of chemosensitive, nociceptive afferents. Chemicals were individually applied (15 mL over 10 s) and thoroughly washed off with 500 mL of warm saline 1-min after the start of application. A 15–30 min wait period was allowed for hemodynamic parameters to return to baseline prior to subsequent interventions.

#### Extracellular neural recording from the intrinsic cardiac nervous system

Custom-made 16-channel linear microelectrode arrays (MicroProbes for Life Science; 25-µm-diameter platinum/iridium electrodes, 16 electrodes/probe, 375-µm interelectrode spacing) were used for in vivo extracellular neural recordings of the VIVGP (Fig. 4), as previously described^48,49,65^ in 5 vehicle and 6 epidural cRTX animals. In short, the probe was gently advanced into the epicardial fat pad and serially connected to a head-stage preamplifier and a 16-channel preamplifier (Model 3600, A-M Systems, Sequim, WA). All signals were continuously recorded and digitized (Cambridge Electronic Design) at a sampling frequency of 20 kHz, and band-pass filtered (0.3−3 kHz). Offline processing and analysis of neural signals were performed using Spike2 software (Cambridge Electronic Design, v10.08), as previously described^48,49,65^. Artifacts (recognized as simultaneous waveforms on all neural recording channels) were removed, and neuronal spikes were identified using a threshold of 2 times the signal-to-noise ratio. Spike sorting was performed using principal components, cluster on measurements, and K-means clustering analysis to identify unique neuronal waveforms^47,144^. Efferent postganglionic parasympathetic neurons in the VIVGP were identified based on their responses to left- or right-sided VNS. Briefly, bipolar spiral cuff electrodes (LivaNova, PLC) were placed around each cervical vagus, and the VIVGP activation threshold current, defined as the VNS current needed to evoke a 10% decrease in heart rate (20 Hz, 1 ms), was determined. Each cervical vagus was then stimulated separately for 1 min at 1 Hz (1 ms, 1× VIVGP activation threshold current). At least 20 min of recovery time was allowed between the two stimulations. Baseline activity during the 2 min before left or right VNS was compared with the 2 min at the start of stimulation (1 min during VNS and 1 min after) using the Skellam statistical test^145^. Neurons that showed significant changes in firing activity (as compared by the Skellam test) upon left or right VNS were identified as postganglionic parasympathetic neurons, while neurons that did not show significant alterations in their activity were considered to be non-VNS responsive. Basal activity of both VNS-responsive and non-VNS-responsive (1 min) was compared between vehicle (*n* = 5) and epidural cRTX (*n* = 6) animals.

#### Effective refractory period measurements and VT/VF inducibility

Atrial and ventricular effective refractory periods (ERP) were measured by extra-stimulus pacing at a drive cycle length (CL) of 450 ms, with S2 decremented by 5 ms, using a pacing catheter placed on the epicardial left atrial appendage and in the right ventricular (RV) apex from the right femoral vein.

VT inducibility was tested in epidural cRTX (*n* = 11), vehicle (*n* = 13), and epidural aRTX (*n* = 12) animals. Inducibility was assessed by programmed stimulation (as is standard in electrophysiology laboratories for testing of inducibility in patients with heart disease)^146^ by an 8-beat drive train (at CL of 450 ms) followed by an S2 extra-stimulus, which was decremented by 10 ms down to a CL of 200 ms or ERP, whichever occurred first. If no VT/VF was induced, a CL of 20 ms above ERP was selected for the extra-stimulus to ensure ventricular capture and the next extra-stimulus (up to S4) was added. VT/VF inducibility was defined as the occurrence of sustained VT (>30 s) or VF requiring defibrillation. Inducible animals were cardioverted if VT/VF did not terminate after 30 s. VT inducibility was tested from RV endocardium and, if non-inducible, also from the LV anterior epicardial border zone region. For acute RTX animals, the same site that induced VT/VF pre-RTX administration was used post-RTX administration to induce VT. Ventricular pacing thresholds were checked and maintained pre- *vs* post-RTX administration (Supplementary Fig. 8).

### Histopathological assessment

LV tissue, macroscopically and electrically (by bipolar voltage criteria, defined as voltage > 1.5 mV^60^) classified as viable, as well as C7-T1 and T12-L1 spinal cords and DRGs, were collected from cRTX (*n* = 5–7) and vehicle-treated infarcted animals (*n* = 5), fixed in 4% paraformaldehyde, and embedded in paraffin. Tissue was sectioned at 5 µm, deparaffinized, rehydrated, and epitopes unmasked at 90 °C in EDTA buffer (Abcam, ab64216). Slides were blocked and incubated overnight at 4 °C with goat anti-calcitonin gene-related peptide (CGRP; 1:1000; Abcam, ab36001), mouse anti-glial fibrillary protein (GFAP; 1:1000; Invitrogen, ASTRO6), rabbit anti-ionized calcium-binding adaptor molecule 1 (Iba1; 1:1000; Biocare Medical, CP 290 A, B), sheep anti-tyrosine hydroxylase (TH; 1:500; MilliporeSigma, AB1542), rabbit anti-purine P2X3 receptor (1:200; Neuromics, RA10109), rabbit anti-Piezo-type mechanosensitive ion channel component 2 (PIEZO2; 1:400; ThermoFisher, PA5-72975) and/or rat anti-cluster of differentiation 3 (CD3; 1:1000; Abcam, ab11089). CGRP was used as a surrogate for TRPV1 expression as it is released by nociceptive sensory/afferent neurons upon TRPV1 activation^137^. Sections were incubated for 2 h at room temperature with Alexa Fluor 488–donkey anti-goat IgG (1:200; Invitrogen, A-11055), Alexa Fluor 555–donkey anti-mouse IgG (1:400; Invitrogen, A-31570), Alexa Fluor 488–donkey anti-rabbit IgG (1:400; Invitrogen, A21206), Alexa Fluor 647–donkey anti-sheep IgG (1:400; Invitrogen, A-21448), and/or CF405S–donkey anti-rat IgG (1:400; Biotium, 20419), respectively and mounted with Antifade Mounting Medium (Vector Laboratories, H-1000-10). Slides were imaged on a Zeiss LSM880 at 20× and 63× magnifications and processed with Zen 2 software (Zeiss). In the spinal cord, CGRP, GFAP, IBA1, and CD3 immunoreactivity were quantified as the fractional area of the dorsal horn. In the DRG, GFAP, CD3, and IBA1 were quantified as the fractional area, and CGRP was quantified as the percentage of neurons in the section containing the greatest area of neurons, respectively. All image analysis was performed with ImageJ (NIH)

### Measurement of plasma liver enzymes and inflammatory markers

For evaluation of liver function, femoral artery blood was collected at the time of terminal studies (4–6 weeks after vehicle or RTX injection and MI) to measure plasma interleukin-12 (IL-12), tumor necrosis factor α (TNF-α), aspartate aminotransferase (AST) and alanine aminotransferase (ALT) concentrations in animals treated with epidural vehicle or RTX prior to MI. Blood was collected into K_2_ EDTA blood collection tubes (BD Vacutainer), and immediately centrifugated at 1500 × *g* for 15 min. Plasma was separated, snap-frozen in liquid nitrogen and stored at −80 °C until assay. IL-12 (P1240, sensitivity 18.2 pg/mL, Quantikine), TNF-α (PTA00, sensitivity 5 pg/mL, Quantikine), AST (MBS739883, sensitivity 0.1 ng/mL, MyBioSource) and ALT (MBS266004, sensitivity 0.06 ng/mL, MyBioSource) levels were measured by ELISA, according to the manufacturer’s instructions.

### Plasma proteomics

#### Sample preparation

After access to the femoral artery was obtained and prior to performing any autonomic or electrophysiological testing, baseline blood was collected in EDTA blood tubes and immediately placed on ice. Samples were centrifuged and plasma was separated and stored in −80 °C. Plasma samples (*n* = 9 for Vehicle + MI; *n* = 10 for cRTX + MI) were prepared by the University of California, Los Angeles (UCLA) Proteome Research Center, using the Mag-Net^147^. Dried peptides were resuspended in 5% formic acid and separated using a PepSep C18 reverse-phase column (150 mm × 150 µm, 1.7 µm particle size) heated to 59 °C. Fractionation was conducted on a Thermofisher Vanquish Neo UHPLC system using a trap-and-elute workflow and a 15 min gradient of increasing acetonitrile (5% acetonitrile to 80% acetonitrile) delivered at a flow rate of 2.45 µL/min. MS/MS spectra were collected using a data-independent analysis (DIA) acquisition method on a Thermo Fisher Orbitrap Astral mass spectrometer^148,149^. MS/MS spectra were acquired using sequential 4 *m/z* windows tiled over a *m/z* range of 380–980 at 80,000 resolution with a normalized HCD energy of 25%, a normalized AGC target of 500%, and a 7 ms maximum injection time. Data were analyzed using the DIA-NN algorithm to search an in silico spectral library generated from the Sus scrofa UniProt reference proteome (ID: UP000008227). Peptide and protein identifications were filtered using an estimated false discovery rate of less than 1%^150^. Comparison testing between conditions was performed using the Fragpipe-Analyst platform using DIA-NN-generated label-free protein abundances^151^.

#### Data analysis

Data analysis was performed in R. Raw counts were normalized with random-forest normalization using R. Differentially expressed proteins were identified using a two-sided Student’s *t*-test. Proteins with a *p-value* < 0.05 were considered statistically significant, and those with *|log2(fold change)| *> 0.5 were identified as differentially expressed proteins. Pathway analysis was performed against the Gene Ontology (GO) database using Rapid Integration of Term Annotation and Network (RITAN, v3.20)^152^. A two-sided *p*-value < 0.05 was used to determine statistically overrepresented pathways.

### Analysis of plasma inflammatory cytokines

Femoral artery blood was collected at the time of terminal experiment (4–6 weeks after vehicle or RTX injection and MI) to measure plasma tumor necrosis factor-α (TNF-α), interleukin-1β (IL-1β), interleukin-6 (IL-6), interleukin-10 (IL-10), and interleukin-12 (IL-12). Blood was collected into K2 EDTA blood collection tubes (BD Vacutainer) and immediately centrifugated at 1500 × g for 15 min. Plasma was separated, snap-frozen in liquid nitrogen and stored at −80 °C until assay. Circulating TNF-α, IL-1β, IL-6, IL-10, and IL-12 were assessed via Porcine Luminex Discovery Assay (R&D Systems) according to the manufacturer’s instructions.

### Statistical analyses

Data are presented as mean ± SEM. For cRTX versus vehicle comparisons, each data point represents an independent biological replicate (one animal); for acute RTX (aRTX) experiments, parameters were measured repeatedly within the same animal before and after RTX administration, and analyzed using paired/repeated-measures tests. After confirmation of normality, paired two-tailed Student’s *t*-test or Wilcoxon signed rank test was used to compare pre- and post-RTX parameters in epidural aRTX animals, depending on Gaussian distribution. Immunohistochemical data were analyzed using unpaired Student’s *t*-tests or Mann–Whitney U test (depending on Gaussian distribution) with df = *n*_1_ + *n*_2_ − 2 for each comparison (derived from the group sizes stated per panel). Unpaired analysis of variance (ANOVA) was used for intergroup comparisons (vehicle *vs* epidural cRTX *vs* Control) and changes in hemodynamic parameters over time were compared using repeated measures ANOVA. Electrophysiological parameters were compared using unpaired Student’s *t*-tests or Mann–Whitney U test (depending on Gaussian distribution) for epidural cRTX *vs* vehicle animals or repeated measures ANOVA in case of serial comparisons in acute RTX studies. BRS data were only included for analysis if *R*^2^ was greater than 0.8 for the slope of the regression line relating blood pressure to heart rate and compared using the Mann–Whitney U test (for group comparisons) or the Friedman test (for serial comparison in acute RTX experiments). Comparison of VT/VF inducibility was performed using the binomial exact test. *P-value* < 0.05 was considered statistically significant.

### Reporting summary

Further information on research design is available in the Nature Portfolio Reporting Summary linked to this article.

## Supplementary information

Supplementary Information

Description of Additional Supplementary Data Files

Supplementary data 1

Supplementary data 2

Reporting Summary

Transparent Peer Review file

## Source data

Source data

## Data Availability

The mass spectrometry proteomics data have been deposited to the ProteomeXchange Consortium via the PRIDE partner repository under accession code MV000099727 (https://massive.ucsd.edu/ProteoSAFe/dataset.jsp?task=9d63769e90804f8091546cfca56052cb). Source data underlying all figures, including individual data points, are provided with this paper in the Source Data file and Supplementary data 1 and 2. Raw acquisition files (electrophysiological recordings, neural recordings, and confocal microscopy images) are large and are available from the corresponding author on request, owing to file-size and format constraints; their summary measurements are fully reported in the Source Data file. Source data are provided with this paper.

## Code availability

Custom code used to process the extracellular neural recordings and to analyze the plasma proteomic data (differential expression, and pathway enrichment) is available at GitHub (https://github.com/jdthoang/vanWeperenV_2026_EpiduralRTX/) and archived at Zenodo (10.5281/zenodo.20711756). Pathway analysis was performed with RITAN (v3.20) in R (v4.4.2). Customized software, iScaldyn (University of Utah), was used to compute activation-recovery intervals and is freely available upon request from Robert L. Lux (robert.lux@utah.edu). Image quantification was performed in ImageJ (NIH, v1.54). No other custom code was generated in this study.

## References

[1] van Weperen, V. Y. H., Ripplinger, C. M. & Vaseghi, M. Autonomic control of ventricular function in health and disease: current state of the art. *Clin. Auton. Res* **33**, 1–27 (2023). 37166736 10.1007/s10286-023-00948-8 PMC10173946

[2] Vaseghi, M. & Shivkumar, K. The role of the autonomic nervous system in sudden cardiac death. *Prog. Cardiovasc Dis.* **50**, 404–419 (2008). 18474284 10.1016/j.pcad.2008.01.003 PMC2752648

[3] Ajijola, O. A. et al. Inflammation, oxidative stress, and glial cell activation characterize stellate ganglia from humans with electrical storm. *JCI Insight* **2**, e94715 (2017). 28931760 10.1172/jci.insight.94715 PMC5621921

[4] Barrett, M. S. et al. Ischemia-reperfusion myocardial infarction induces remodeling of left cardiac-projecting stellate ganglia neurons. *Am. J. Physiol. Heart Circ. Physiol.* **326**, H166-179 (2024). 37947434 10.1152/ajpheart.00582.2023 PMC11213476

[5] Vaseghi, M., Lux, R. L., Mahajan, A. & Shivkumar, K. Sympathetic stimulation increases dispersion of repolarization in humans with myocardial infarction. *Am. J. Physiol. Heart Circ. Physiol.* **302**, H1838–H1846 (2012). 22345568 10.1152/ajpheart.01106.2011 PMC3362058

[6] Ben-David, J. & Zipes, D. P. Differential response to right and left ansae subclaviae stimulation of early afterdepolarizations and ventricular tachycardia induced by cesium in dogs. *Circulation* **78**, 1241–1250 (1988). 3180380 10.1161/01.cir.78.5.1241

[7] Priori, S. G., Mantica, M. & Schwartz, P. J. Delayed afterdepolarizations elicited in vivo by left stellate ganglion stimulation. *Circulation* **78**, 178–185 (1988). 3383403 10.1161/01.cir.78.1.178

[8] Opthof, T. et al. Dispersion of refractoriness in normal and ischaemic canine ventricle: effects of sympathetic stimulation. *Cardiovasc. Res.* **27**, 1954–1960 (1993). 8287403 10.1093/cvr/27.11.1954

[9] Wang, H. J., Rozanski, G. J. & Zucker, I. H. Cardiac sympathetic afferent reflex control of cardiac function in normal and chronic heart failure states. *J. Physiol.* **595**, 2519–2534 (2017). 28116751 10.1113/JP273764 PMC5390865

[10] Wang, H. J., Wang, W., Cornish, K. G., Rozanski, G. J. & Zucker, I. H. Cardiac sympathetic afferent denervation attenuates cardiac remodeling and improves cardiovascular dysfunction in rats with heart failure. *Hypertension* **64**, 745–755 (2014). 24980663 10.1161/HYPERTENSIONAHA.114.03699 PMC4162756

[11] Chen, W. W. et al. Cardiac sympathetic afferent reflex and its implications for sympathetic activation in chronic heart failure and hypertension. *Acta Physiol.* **213**, 778–794 (2015). 10.1111/apha.12447 25598170

[12] Zhu, G. Q., Zucker, I. H. & Wang, W. Central AT1 receptors are involved in the enhanced cardiac sympathetic afferent reflex in rats with chronic heart failure. *Basic Res. Cardiol.* **97**, 320–326 (2002). 12111042 10.1007/s00395-002-0353-z

[13] Wang, W. Z., Gao, L., Wang, H. J., Zucker, I. H. & Wang, W. Interaction between cardiac sympathetic afferent reflex and chemoreflex is mediated by the NTS AT1 receptors in heart failure. *Am. J. Physiol. Heart Circ. Physiol.* **295**, H1216–H1226 (2008). 18660444 10.1152/ajpheart.00557.2008 PMC2544509

[14] Szallasi, A. & Blumberg, P. M. Resiniferatoxin, a phorbol-related diterpene, acts as an ultrapotent analog of capsaicin, the irritant constituent in red pepper. *Neuroscience* **30**, 515–520 (1989). 2747924 10.1016/0306-4522(89)90269-8

[15] Bates, B. D. et al. Prolonged analgesic response of cornea to topical resiniferatoxin, a potent TRPV1 agonist. *Pain* **149**, 522–528 (2010). 20403666 10.1016/j.pain.2010.03.024 PMC2913152

[16] Brown, D. C., Agnello, K. & Iadarola, M. J. Intrathecal resiniferatoxin in a dog model: efficacy in bone cancer pain. *Pain* **156**, 1018–1024 (2015). 25659068 10.1097/j.pain.0000000000000115 PMC4431903

[17] Brown, D. C. et al. Physiologic and antinociceptive effects of intrathecal resiniferatoxin in a canine bone cancer model. *Anesthesiology* **103**, 1052–1059 (2005). 16249680 10.1097/00000542-200511000-00020

[18] Brown, J. D. et al. CT-guided injection of a TRPV1 agonist around dorsal root ganglia decreases pain transmission in swine. *Sci. Transl. Med.* **7**, 305ra145 (2015). 26378245 10.1126/scitranslmed.aac6589 PMC4854290

[19] Caterina, M. J. et al. The capsaicin receptor: a heat-activated ion channel in the pain pathway. *Nature* **389**, 816–824 (1997). 9349813 10.1038/39807

[20] Bencsik, P. et al. Myocardial ischaemia reperfusion injury and cardioprotection in the presence of sensory neuropathy: therapeutic options. *Br. J. Pharm.* **177**, 5336–5356 (2020). 10.1111/bph.15021 PMC7680004 32059259

[21] Szabados, T. et al. Capsaicin-sensitive sensory nerves and the TRPV1 ion channel in cardiac physiology and pathologies. *Int. J. Mol. Sci.* **21**, 4472 (2020). 32586044 10.3390/ijms21124472 PMC7352834

[22] Stueber, T. et al. Differential cytotoxicity and intracellular calcium-signalling following activation of the calcium-permeable ion channels TRPV1 and TRPA1. *Cell Calcium* **68**, 34–44 (2017). 29129206 10.1016/j.ceca.2017.10.003

[23] Zhai, K., Liskova, A., Kubatka, P. & Busselberg, D. Calcium entry through TRPV1: a potential target for the regulation of proliferation and apoptosis in cancerous and healthy cells. *Int. J. Mol. Sci.* **21**, 10.3390/ijms21114177 (2020). 10.3390/ijms21114177 PMC7312732 32545311

[24] Bautista, D. & Julius, D. Fire in the hole: pore dilation of the capsaicin receptor TRPV1. *Nat. Neurosci.* **11**, 528–529 (2008). 18437189 10.1038/nn0508-528

[25] Karai, L. et al. Deletion of vanilloid receptor 1-expressing primary afferent neurons for pain control. *J. Clin. Invest.* **113**, 1344–1352 (2004). 15124026 10.1172/JCI20449 PMC398431

[26] Karai, L. J., Russell, J. T., Iadarola, M. J. & Olah, Z. Vanilloid receptor 1 regulates multiple calcium compartments and contributes to Ca2+-induced Ca2+ release in sensory neurons. *J. Biol. Chem.* **279**, 16377–16387 (2004). 14963041 10.1074/jbc.M310891200

[27] Olah, Z. et al. Ligand-induced dynamic membrane changes and cell deletion conferred by vanilloid receptor 1. *J. Biol. Chem.* **276**, 11021–11030 (2001). 11124944 10.1074/jbc.M008392200

[28] Sapio, M. R. et al. Pain control through selective chemo-axotomy of centrally projecting TRPV1+ sensory neurons. *J. Clin. Invest.* **128**, 1657–1670 (2018). 29408808 10.1172/JCI94331 PMC5873867

[29] Yamaguchi, T. et al. Thoracic dorsal root ganglion application of resiniferatoxin reduces myocardial ischemia-induced ventricular arrhythmias. *Biomedicines* **11**, 10.3390/biomedicines11102720 (2023). 10.3390/biomedicines11102720 PMC10604235 37893094

[30] Dusi, V., De Ferrari, G. M. & Schwartz, P. J. There are 100 ways by which the sympathetic nervous system can trigger life-threatening arrhythmias. *Eur. Heart J.* **41**, 2180–2182 (2020). 31985796 10.1093/eurheartj/ehz950

[31] Gardner, R. T., Ripplinger, C. M., Myles, R. C. & Habecker, B. A. Molecular mechanisms of sympathetic remodeling and arrhythmias. *Circ. Arrhythm. Electrophysiol.* **9**, e001359 (2016). 26810594 10.1161/CIRCEP.115.001359 PMC4730917

[32] Armour, J. A. & Ardell, J. L. *Basic and Clinical Neurocardiology* (Oxford University Press, 2004).

[33] Maggi, C. A. Tachykinins and calcitonin gene-related peptide (CGRP) as co-transmitters released from peripheral endings of sensory nerves. *Prog. Neurobiol.* **45**, 1–98 (1995). 7716258 10.1016/0301-0082(94)e0017-b

[34] Gibson, S. J. et al. Calcitonin gene-related peptide immunoreactivity in the spinal cord of man and of eight other species. *J. Neurosci.* **4**, 3101–3111 (1984). 6209366 10.1523/JNEUROSCI.04-12-03101.1984 PMC6564846

[35] Schaible, H. G. *Encyclopedia of Pain* (eds Gebhart, G. F. & Schmidt, R. F.) (Springer, 2013).

[36] Mannes, A. J. et al. Treatment of intractable cancer pain with resiniferatoxin - an interim study. *NEJM Evid.* **4**, EVIDoa2400423 (2025). 40423401 10.1056/EVIDoa2400423

[37] Szabo, T., Olah, Z., Iadarola, M. J. & Blumberg, P. M. Epidural resiniferatoxin induced prolonged regional analgesia to pain. *Brain Res.* **840**, 92–98 (1999). 10517956 10.1016/s0006-8993(99)01763-1

[38] Unger, M. D. et al. Unilateral epidural targeting of resiniferatoxin induces bilateral neurolysis of spinal nociceptive afferents. *Pain. Med.* **20**, 897–906 (2019). 30590777 10.1093/pm/pny276

[39] Foreman, R. D. Mechanisms of cardiac pain. *Annu. Rev. Physiol.* **61**, 143–167 (1999). 10099685 10.1146/annurev.physiol.61.1.143

[40] La Rovere, M. T., Bigger, J. T. Jr., Marcus, F. I., Mortara, A. & Schwartz, P. J. Baroreflex sensitivity and heart-rate variability in prediction of total cardiac mortality after myocardial infarction. ATRAMI (Autonomic Tone and Reflexes After Myocardial Infarction) Investigators. *Lancet* **351**, 478–484 (1998). 9482439 10.1016/s0140-6736(97)11144-8

[41] Mortara, A. et al. Arterial baroreflex modulation of heart rate in chronic heart failure: clinical and hemodynamic correlates and prognostic implications. *Circulation* **96**, 3450–3458 (1997). 9396441 10.1161/01.cir.96.10.3450

[42] Schwartz, P. J. et al. Autonomic mechanisms and sudden death. New insights from analysis of baroreceptor reflexes in conscious dogs with and without a myocardial infarction. *Circulation* **78**, 969–979 (1988). 3168199 10.1161/01.cir.78.4.969

[43] De Ferrari, G. M. et al. Baroreflex sensitivity predicts long-term cardiovascular mortality after myocardial infarction even in patients with preserved left ventricular function. *J. Am. Coll. Cardiol.* **50**, 2285–2290 (2007). 18068036 10.1016/j.jacc.2007.08.043

[44] Rajendran, P. S. et al. Myocardial infarction induces structural and functional remodelling of the intrinsic cardiac nervous system. *J. Physiol.* **594**, 321–341 (2016). 26572244 10.1113/JP271165 PMC4713729

[45] Ardell, J. L. & Armour, J. A. Neurocardiology: structure-based function. *Compr. Physiol.* **6**, 1635–1653 (2016). 27783854 10.1002/cphy.c150046

[46] Giannino, G. et al. The intrinsic cardiac nervous system: from pathophysiology to therapeutic implications. *Biology* **13**, 10.3390/biology13020105 (2024). 10.3390/biology13020105 PMC10887082 38392323

[47] Beaumont, E. et al. Network interactions within the canine intrinsic cardiac nervous system: implications for reflex control of regional cardiac function. *J. Physiol.* **591**, 4515–4533 (2013). 23818689 10.1113/jphysiol.2013.259382 PMC3784196

[48] Hoang, J. D. et al. Antiarrhythmic mechanisms of epidural blockade after myocardial infarction. *Circ. Res.* **135**, e57–e75 (2024). 38939925 10.1161/CIRCRESAHA.123.324058 PMC11257785

[49] Vaseghi, M. et al. Parasympathetic dysfunction and antiarrhythmic effect of vagal nerve stimulation following myocardial infarction. *JCI Insight* **2**, e86715 (2017). 28814663 10.1172/jci.insight.86715 PMC5621871

[50] Yagishita, D. et al. Sympathetic nerve stimulation, not circulating norepinephrine, modulates T-peak to T-end interval by increasing global dispersion of repolarization. *Circ. Arrhythm. Electrophysiol.* **8**, 174–185 (2015). 25532528 10.1161/CIRCEP.114.002195 PMC4405126

[51] Prabhu, S. D. & Frangogiannis, N. G. The biological basis for cardiac repair after myocardial infarction: from inflammation to fibrosis. *Circ. Res.* **119**, 91–112 (2016). 27340270 10.1161/CIRCRESAHA.116.303577 PMC4922528

[52] Gao, C. et al. Inflammatory and apoptotic remodeling in autonomic nervous system following myocardial infarction. *PLoS ONE* **12**, e0177750 (2017). 28542617 10.1371/journal.pone.0177750 PMC5436752

[53] Wang, M. et al. Increased inflammation promotes ventricular arrhythmia through aggravating left stellate ganglion remodeling in a canine ischemia model. *Int. J. Cardiol.* **248**, 286–293 (2017). 28826800 10.1016/j.ijcard.2017.08.011

[54] Dutta, P. et al. Myocardial infarction accelerates atherosclerosis. *Nature* **487**, 325–329 (2012). 22763456 10.1038/nature11260 PMC3401326

[55] Fischer, A. et al. ZAP70: a master regulator of adaptive immunity. *Semin. Immunopathol.* **32**, 107–116 (2010). 20135127 10.1007/s00281-010-0196-x

[56] Goodfellow, H. S. et al. The catalytic activity of the kinase ZAP-70 mediates basal signaling and negative feedback of the T cell receptor pathway. *Sci. Signal.* **8**, ra49 (2015). 25990959 10.1126/scisignal.2005596 PMC4445242

[57] Neurath, M. F. & Berg, L. J. VAV1 as a putative therapeutic target in autoimmune and chronic inflammatory diseases. *Trends Immunol.* **45**, 580–596 (2024). 39060140 10.1016/j.it.2024.06.004

[58] Gilbert, C. J., Longenecker, J. Z. & Accornero, F. ERK1/2: an integrator of signals that alters cardiac homeostasis and growth. *Biology* **10,**10.3390/biology10040346 (2021). 10.3390/biology10040346 PMC8072600 33923899

[59] Saddic, L. A. et al. Progression of myocardial ischemia leads to unique changes in immediate-early gene expression in the spinal cord dorsal horn. *Am. J. Physiol. Heart Circ. Physiol.* **315**, H1592–H1601 (2018). 30216122 10.1152/ajpheart.00337.2018 PMC6336975

[60] Marchlinski, F. E., Callans, D. J., Gottlieb, C. D. & Zado, E. Linear ablation lesions for control of unmappable ventricular tachycardia in patients with ischemic and nonischemic cardiomyopathy. *Circulation* **101**, 1288–1296 (2000). 10725289 10.1161/01.cir.101.11.1288

[61] Herre, J. M & Thames, M. D. Responses of sympathetic nerves to programmed ventricular stimulation. *J. Am. Coll. Cardiol.* **9**, 147–153 (1987). 2432106 10.1016/s0735-1097(87)80093-1

[62] Taylor, J. A., Morillo, C. A., Eckberg, D. L. & Ellenbogen, K. A. Higher sympathetic nerve activity during ventricular (VVI) than during dual-chamber (DDD) pacing. *J. Am. Coll. Cardiol.* **28**, 1753–1758 (1996). 8962562 10.1016/s0735-1097(96)00389-0

[63] Do, D. H. et al. Thoracic epidural anesthesia can be effective for the short-term management of ventricular tachycardia storm. *J. Am. Heart Assoc.* **6**, e007080 (2017). 29079570 10.1161/JAHA.117.007080 PMC5721785

[64] Kang, K. W. Successful neural modulation of bedside modified thoracic epidural anesthesia for ventricular tachycardia electrical storm. *Acute Crit. Care* **39**, 643–646 (2022). 35791654 10.4266/acc.2021.01683 PMC11617849

[65] Salavatian, S. et al. Myocardial infarction reduces cardiac nociceptive neurotransmission through the vagal ganglia. *JCI Insight* **7**, 10.1172/jci.insight.155747 (2022). 10.1172/jci.insight.155747 PMC8876456 35015733

[66] van Weperen, V. Y. H. & Vaseghi, M. Cardiac vagal afferent neurotransmission in health and disease: review and knowledge gaps. *Front. Neurosci.* **17**, 1–10 (2023). 10.3389/fnins.2023.1192188 PMC10282187 37351426

[67] Cao, J. M. et al. Nerve sprouting and sudden cardiac death. *Circ. Res.* **86**, 816–821 (2000). 10764417 10.1161/01.res.86.7.816

[68] Olivas, A. et al. Myocardial infarction causes transient cholinergic transdifferentiation of cardiac sympathetic nerves via gp130. *J. Neurosci.* **36**, 479–488 (2016). 26758839 10.1523/JNEUROSCI.3556-15.2016 PMC4710771

[69] Gao, L., Schultz, H. D., Patel, K. P., Zucker, I. H. & Wang, W. Augmented input from cardiac sympathetic afferents inhibits baroreflex in rats with heart failure. *Hypertension* **45**, 1173–1181 (2005). 15897358 10.1161/01.HYP.0000168056.66981.c2

[70] Zahner, M. R., Li, D. P., Chen, S. R. & Pan, H. L. Cardiac vanilloid receptor 1-expressing afferent nerves and their role in the cardiogenic sympathetic reflex in rats. * J. Physiol.* **551**, 515–523 (2003). 12829722 10.1113/jphysiol.2003.048207 PMC2343227

[71] Pan, H. L., Chen, S. R., Scicli, G. M. & Carretero, O. A. Cardiac interstitial bradykinin release during ischemia is enhanced by ischemic preconditioning. *Am. J. Physiol. Heart Circ. Physiol.* **279**, H116–H121 (2000). 10899048 10.1152/ajpheart.2000.279.1.H116

[72] Gardner, R. T. et al. Targeting protein tyrosine phosphatase sigma after myocardial infarction restores cardiac sympathetic innervation and s arrhythmias. *Nat. Commun.* **6**, 6235 (2015). 25639594 10.1038/ncomms7235 PMC4315356

[73] Cao, J. M. et al. Relationship between regional cardiac hyperinnervation and ventricular arrhythmia. *Circulation* **101**, 1960–1969 (2000). 10779463 10.1161/01.cir.101.16.1960

[74] Rubart, M. & Zipes, D. P. Mechanisms of sudden cardiac death. *J. Clin. Invest.* **115**, 2305–2315 (2005). 16138184 10.1172/JCI26381 PMC1193893

[75] Warner, M. R., Wisler, P. L., Hodges, T. D., Watanabe, A. M. & Zipes, D. P. Mechanisms of denervation supersensitivity in regionally denervated canine hearts. *Am. J. Physiol.* **264**, H815–H820 (1993). 8384424 10.1152/ajpheart.1993.264.3.H815

[76] Kammerling, J. J. et al. Denervation supersensitivity of refractoriness in noninfarcted areas apical to transmural myocardial infarction. *Circulation* **76**, 383–393 (1987). 3038369 10.1161/01.cir.76.2.383

[77] Inoue, H. & Zipes, D. P. Time course of denervation of efferent sympathetic and vagal nerves after occlusion of the coronary artery in the canine heart. *Circ. Res.* **62**, 1111–1120 (1988). 3383360 10.1161/01.res.62.6.1111

[78] Ide, R., Saiki, C., Makino, M. & Matsumoto, S. TRPV1 receptor expression in cardiac vagal afferent neurons of infant rats. *Neurosci. Lett.* **507**, 67–71 (2012). 22178141 10.1016/j.neulet.2011.11.055

[79] Mohammed, M., Madden, C. J., Andresen, M. C. & Morrison, S. F. Activation of TRPV1 in nucleus tractus solitarius reduces brown adipose tissue thermogenesis, arterial pressure, and heart rate. *Am. J. Physiol. Regul. Integr. Comp. Physiol.* **315**, R134–R143 (2018). 29590555 10.1152/ajpregu.00049.2018 PMC6087884

[80] Wu, C. et al. Spinal cord astrocytes regulate myocardial ischemia-reperfusion injury. *Basic Res. Cardiol.* **117**, 56 (2022). 36367592 10.1007/s00395-022-00968-x PMC10139732

[81] Ping Dai, R., Ping He, B., Thameem Dheen, S. & Tay, S. S. Acute cardiac injury induces glial cell response and activates extracellular signaling-regulated kinase-1 and −2 in the spinal cord of Wistar rats. *Neurosci. Lett.* **366**, 34–38 (2004). 15265585 10.1016/j.neulet.2004.05.018

[82] Pereyra, K. et al. Chemogenetic inhibition of NTS astrocytes normalizes cardiac autonomic control and ameliorate hypertension during chronic intermittent hypoxia. *Biol. Res.* **56**, 57 (2023). 37932867 10.1186/s40659-023-00463-0 PMC10626729

[83] Marina, N. et al. Purinergic signalling in the rostral ventro-lateral medulla controls sympathetic drive and contributes to the progression of heart failure following myocardial infarction in rats. *Basic Res. Cardiol.* **108**, 317 (2013). 23187902 10.1007/s00395-012-0317-x PMC3540348

[84] Bezzi, P. et al. CXCR4-activated astrocyte glutamate release via TNFalpha: amplification by microglia triggers neurotoxicity. *Nat. Neurosci.* **4**, 702–710 (2001). 11426226 10.1038/89490

[85] Pascual, O., Ben Achour, S., Rostaing, P., Triller, A. & Bessis, A. Microglia activation triggers astrocyte-mediated modulation of excitatory neurotransmission. *Proc. Natl. Acad. Sci. USA* **109**, E197–E205 (2012). 22167804 10.1073/pnas.1111098109 PMC3268269

[86] Enes, J. et al. Satellite glial cells modulate cholinergic transmission between sympathetic neurons. *PLoS ONE* **15**, e0218643 (2020). 32017764 10.1371/journal.pone.0218643 PMC6999876

[87] Zhou, Z. et al. Inhibition of satellite glial cell activation in stellate ganglia prevents ventricular arrhythmogenesis and remodeling after myocardial infarction. *Circ. Arrhythm. Electrophysiol*. e013866 10.1161/CIRCEP.125.013866 (2025). 10.1161/CIRCEP.125.013866 41025235

[88] Zhang, X., Chen, Y., Wang, C. & Huang, L. Y. Neuronal somatic ATP release triggers neuron-satellite glial cell communication in dorsal root ganglia. *Proc. Natl. Acad. Sci. USA* **104**, 9864–9869 (2007). 17525149 10.1073/pnas.0611048104 PMC1887586

[89] Howard-Quijano, K. et al. Spinal cord stimulation reduces ventricular arrhythmias by attenuating reactive gliosis and activation of spinal interneurons. *JACC Clin. Electrophysiol.* **7**, 1211–1225 (2021). 34454884 10.1016/j.jacep.2021.05.016 PMC8542625

[90] Peng, C. et al. Neuroimmune modulation mediated by IL-6: A potential target for the treatment of ischemia-induced ventricular arrhythmias. *Heart Rhythm* **21**, 610–619 (2024). 38160759 10.1016/j.hrthm.2023.12.020

[91] Deng, J. et al. The effects of interleukin 17A on left stellate ganglion remodeling are mediated by neuroimmune communication in normal structural hearts. *Int. J. Cardiol.* **279**, 64–71 (2019). 30642646 10.1016/j.ijcard.2019.01.010

[92] Wang, M. et al. Microglia-mediated neuroinflammation: a potential target for the treatment of cardiovascular diseases. *J. Inflamm. Res.* **15**, 3083–3094 (2022). 35642214 10.2147/JIR.S350109 PMC9148574

[93] Thackeray, J. T. et al. Myocardial inflammation predicts remodeling and neuroinflammation after myocardial infarction. *J. Am. Coll. Cardiol.* **71**, 263–275 (2018). 29348018 10.1016/j.jacc.2017.11.024

[94] Arslan, F., de Kleijn, D. P. & Pasterkamp, G. Innate immune signaling in cardiac ischemia. *Nat. Rev. Cardiol.* **8**, 292–300 (2011). 21448140 10.1038/nrcardio.2011.38

[95] Faxon, D. P. et al. The effect of blockade of the CD11/CD18 integrin receptor on infarct size in patients with acute myocardial infarction treated with direct angioplasty: the results of the HALT-MI study. *J. Am. Coll. Cardiol.* **40**, 1199–1204 (2002). 12383565 10.1016/s0735-1097(02)02136-8

[96] Christia, P. & Frangogiannis, N. G. Targeting inflammatory pathways in myocardial infarction. *Eur. J. Clin. Invest.* **43**, 986–995 (2013). 23772948 10.1111/eci.12118 PMC3745791

[97] Saxena, A., Russo, I. & Frangogiannis, N. G. Inflammation as a therapeutic target in myocardial infarction: learning from past failures to meet future challenges. *Transl. Res.* **167**, 152–166 (2016). 26241027 10.1016/j.trsl.2015.07.002 PMC4684426

[98] Jiang, X. et al. Loss of MD1 exacerbates myocardial ischemia/reperfusion injury and susceptibility to ventricular arrhythmia. *Eur. J. Pharm.* **844**, 79–86 (2019). 10.1016/j.ejphar.2018.11.025 30458167

[99] Fu, H., Shuai, W., Kong, B., Jiang, X. & Huang, H. MD1 depletion predisposes to ventricular arrhythmias in the setting of myocardial infarction. *Heart Lung Circ.* **30**, 869–881 (2021). 33257269 10.1016/j.hlc.2020.09.938

[100] Karahaliou, A. et al. Ventricular arrhythmias and antioxidative medication: experimental study. *Hellenic J. Cardiol.* **49**, 320–328 (2008). 18846922

[101] Li, X. et al. Interplay of pro-inflammatory cytokines, pro-inflammatory microparticles and oxidative stress and recurrent ventricular arrhythmias in elderly patients after coronary stent implantations. *Cytokine* **137**, 155345 (2021). 33137563 10.1016/j.cyto.2020.155345

[102] Szyller, J., Jagielski, D. & Bil-Lula, I. Antioxidants in arrhythmia treatment-still a controversy? A review of selected clinical and laboratory research. *Antioxidants* **11**, 10.3390/antiox11061109 (2022). 10.3390/antiox11061109 PMC9220256 35740006

[103] Murphy, M. P. Antioxidants as therapies: can we improve on nature? *Free Radic. Biol. Med.* **66**, 20–23 (2014). 23603661 10.1016/j.freeradbiomed.2013.04.010

[104] Bubb, K. J., Drummond, G. R. & Figtree, G. A. New opportunities for targeting redox dysregulation in cardiovascular disease. *Cardiovasc. Res.* **116**, 532–544 (2020). 31297507 10.1093/cvr/cvz183

[105] Padfield, G. J. et al. Cardiovascular effects of tumour necrosis factor alpha antagonism in patients with acute myocardial infarction: a first in human study. *Heart* **99**, 1330–1335 (2013). 23574969 10.1136/heartjnl-2013-303648 PMC3756454

[106] Mann, D. L. et al. Targeted anticytokine therapy in patients with chronic heart failure: results of the Randomized Etanercept Worldwide Evaluation (RENEWAL). *Circulation* **109**, 1594–1602 (2004). 15023878 10.1161/01.CIR.0000124490.27666.B2

[107] Chung, E. S. et al. Randomized, double-blind, placebo-controlled, pilot trial of infliximab, a chimeric monoclonal antibody to tumor necrosis factor-alpha, in patients with moderate-to-severe heart failure: results of the anti-TNF Therapy Against Congestive Heart Failure (ATTACH) trial. *Circulation* **107**, 3133–3140 (2003). 12796126 10.1161/01.CIR.0000077913.60364.D2

[108] Perrotta, S. et al. A heart-brain-spleen axis controls cardiac remodeling to hypertensive stress. *Immunity* **58**, 648–665 e647 (2025). 40023160 10.1016/j.immuni.2025.02.013

[109] Huston, J. M. & Tracey, K. J. The pulse of inflammation: heart rate variability, the cholinergic anti-inflammatory pathway and implications for therapy. *J. Intern. Med.* **269**, 45–53 (2011). 21158977 10.1111/j.1365-2796.2010.02321.x PMC4527046

[110] Borovikova, L. V. et al. Vagus nerve stimulation attenuates the systemic inflammatory response to endotoxin. *Nature* **405**, 458–462 (2000). 10839541 10.1038/35013070

[111] Rosas-Ballina, M. et al. Acetylcholine-synthesizing T cells relay neural signals in a vagus nerve circuit. *Science* **334**, 98–101 (2011). 21921156 10.1126/science.1209985 PMC4548937

[112] Fallavollita, J. A. et al. Regional myocardial sympathetic denervation predicts the risk of sudden cardiac arrest in ischemic cardiomyopathy. *J. Am. Coll. Cardiol.* **63**, 141–149 (2014). 24076296 10.1016/j.jacc.2013.07.096 PMC3954563

[113] Taggart, P. et al. Effect of adrenergic stimulation on action potential duration restitution in humans. *Circulation* **107**, 285–289 (2003). 12538429 10.1161/01.cir.0000044941.13346.74

[114] Taggart, P., Sutton, P., Lab, M., Dean, J. & Harrison, F. Interplay between adrenaline and interbeat interval on ventricular repolarisation in intact heart in vivo. *Cardiovasc. Res.* **24**, 884–895 (1990). 2272066 10.1093/cvr/24.11.884

[115] Campos, F. O., Shiferaw, Y., dos Santos, R. W., Plank, G. & Bishop, M. J. Microscopic isthmuses and fibrosis within the border zone of infarcted hearts promote calcium-mediated ectopy and conduction block. *Front. Phys.* **6**, 10.3389/fphy.2018.00057 (2018).

[116] Amoni, M. et al. Discrete sites of frequent premature ventricular complexes cluster within the infarct border zone and coincide with high frequency of delayed afterdepolarizations under adrenergic stimulation. *Heart Rhythm* **18**, 1976–1987 (2021). 34371193 10.1016/j.hrthm.2021.07.067

[117] Marrouche, N. F. et al. Mode of initiation and ablation of ventricular fibrillation storms in patients with ischemic cardiomyopathy. *J. Am. Coll. Cardiol.* **43**, 1715–1720 (2004). 15120835 10.1016/j.jacc.2004.03.004

[118] Vaseghi, M. et al. Cardiac sympathetic denervation in patients with refractory ventricular arrhythmias or electrical storm: intermediate and long-term follow-up. *Heart Rhythm* **11**, 360–366 (2014). 24291775 10.1016/j.hrthm.2013.11.028 PMC4253031

[119] Irie, T. et al. Cardiac sympathetic innervation via middle cervical and stellate ganglia and antiarrhythmic mechanism of bilateral stellectomy. *Am. J. Physiol. Heart Circ. Physiol.* **312**, 392–405 (2017). 10.1152/ajpheart.00644.2016 PMC5402012 28011590

[120] Campese, V. M. & Kogosov, E. Renal afferent denervation prevents hypertension in rats with chronic renal failure. *Hypertension* **25**, 878–882 (1995). 7721447 10.1161/01.hyp.25.4.878

[121] Donovan, M. K., Wyss, J. M. & Winternitz, S. R. Localization of renal sensory neurons using the fluorescent dye technique. *Brain Res.* **259**, 119–122 (1983). 6824924 10.1016/0006-8993(83)91072-7

[122] Weiss, M. L. & Chowdhury, S. I. The renal afferent pathways in the rat: a pseudorabies virus study. *Brain Res.* **812**, 227–241 (1998). 9813344 10.1016/s0006-8993(98)00950-0

[123] Wu, M. et al. Innervation of nociceptor neurons in the spleen promotes germinal center responses and humoral immunity. *Cell* **187**, 2935–2951 e2919 (2024). 38772371 10.1016/j.cell.2024.04.027

[124] Magni, F. & Carobi, C. The afferent and preganglionic parasympathetic innervation of the rat liver, demonstrated by the retrograde transport of horseradish peroxidase. *J. Auton. Nerv. Syst.* **8**, 237–260 (1983). 6668387 10.1016/0165-1838(83)90108-x

[125] Carobi, C. & Magni, F. The afferent innervation of the liver: a horseradish peroxidase study in the rat. *Neurosci. Lett.* **23**, 269–274 (1981). 6167912 10.1016/0304-3940(81)90009-4

[126] Carobi, C. & Magni, F. Capsaicin-sensitive afferent vagal neurons innervating the rat liver. *Neurosci. Lett.* **62**, 261–265 (1985). 4088535 10.1016/0304-3940(85)90365-9

[127] Kim, S. H. et al. Mapping of the sensory innervation of the mouse lung by specific vagal and dorsal root ganglion neuronal subsets. *eNeuro* **9**, 10.1523/ENEURO.0026-22.2022 (2022). 10.1523/ENEURO.0026-22.2022 PMC9015009 35365503

[128] Oh, E. J., Mazzone, S. B., Canning, B. J. & Weinreich, D. Reflex regulation of airway sympathetic nerves in guinea-pigs. *J. Physiol.* **573**, 549–564 (2006). 16581869 10.1113/jphysiol.2005.104661 PMC1779716

[129] Weaver, L. C. Cardiopulmonary sympathetic afferent influences on renal nerve activity. *Am. J. Physiol.* **233**, H592–H599 (1977). 200148 10.1152/ajpheart.1977.233.5.H592

[130] Kostreva, D. R., Hopp, F. A. & Kampine, J. P. Depressor responses to stimulation of sympathetic afferents in monkeys and dogs. *Am. J. Physiol.* **240**, R23–R28 (1981). 7457628 10.1152/ajpregu.1981.240.1.R23

[131] Wang, Y., Soukhova, G., Proctor, M., Walker, J. & Yu, J. Bradykinin causes hypotension by activating pulmonary sympathetic afferents in the rabbit. *J. Appl Physiol.* **95**, 233–240 (2003). 12679362 10.1152/japplphysiol.00584.2002

[132] Soukhova-O’Hare, G. K., Zhang, J. W., Gozal, D. & Yu, J. Bradykinin B2 receptors mediate pulmonary sympathetic afferents induced reflexes in rabbits. *Life Sci.* **78**, 1990–1997 (2006). 16289619 10.1016/j.lfs.2005.08.035

[133] Hooper, J. S. et al. Characterization of cardiovascular reflexes evoked by airway stimulation with allylisothiocyanate, capsaicin, and ATP in Sprague-Dawley rats. *J. Appl. Physiol.* **120**, 580–591 (2016). 26718787 10.1152/japplphysiol.00944.2015 PMC4868373

[134] Shanks, J. et al. Sympatho-excitatory response to pulmonary chemosensitive spinal afferent activation in anesthetized, vagotomized rats. *Physiol. Rep.* **6**, e13742 (2018). 29906340 10.14814/phy2.13742 PMC6003656

[135] Strecker, T., Messlinger, K., Weyand, M. & Reeh, P. W. Role of different proton-sensitive channels in releasing calcitonin gene-related peptide from isolated hearts of mutant mice. *Cardiovasc. Res.* **65**, 405–410 (2005). 15639479 10.1016/j.cardiores.2004.10.013

[136] Meng, J., Wang, J., Lawrence, G. & Dolly, J. O. Synaptobrevin I mediates exocytosis of CGRP from sensory neurons and inhibition by botulinum toxins reflects their anti-nociceptive potential. *J. Cell Sci.* **120**, 2864–2874 (2007). 17666428 10.1242/jcs.012211

[137] Meng, J. et al. Activation of TRPV1 mediates calcitonin gene-related peptide release, which excites trigeminal sensory neurons and is attenuated by a retargeted botulinum toxin with anti-nociceptive potential. *J. Neurosci.* **29**, 4981–4992 (2009). 19369567 10.1523/JNEUROSCI.5490-08.2009 PMC6665337

[138] Kilo, S., Harding-Rose, C., Hargreaves, K. M. & Flores, C. M. Peripheral CGRP release as a marker for neurogenic inflammation: a model system for the study of neuropeptide secretion in rat paw skin. *Pain* **73**, 201–207 (1997). 9415506 10.1016/S0304-3959(97)00108-5

[139] Szallasi, A., Cortright, D. N., Blum, C. A. & Eid, S. R. The vanilloid receptor TRPV1: 10 years from channel cloning to antagonist proof-of-concept. *Nat. Rev. Drug Discov.* **6**, 357–372 (2007). 17464295 10.1038/nrd2280

[140] Szallasi, A. & Blumberg, P. M. Vanilloid (Capsaicin) receptors and mechanisms. *Pharm. Rev.* **51**, 159–212 (1999). 10353985

[141] Kee, Z., Kodji, X. & Brain, S. D. The role of calcitonin gene related peptide (CGRP) in neurogenic vasodilation and its cardioprotective effects. *Front. Physiol.* **9**, 1249 (2018). 30283343 10.3389/fphys.2018.01249 PMC6156372

[142] Haws, C. W. & Lux, R. L. Correlation between in vivo transmembrane action potential durations and activation-recovery intervals from electrograms. Effects of interventions that alter repolarization time. *Circulation* **81**, 281–288 (1990). 2297832 10.1161/01.cir.81.1.281

[143] Smyth, H. S., Sleight, P. & Pickering, G. W. Reflex regulation of arterial pressure during sleep in man. A quantitative method of assessing baroreflex sensitivity. *Circ. Res.* **24**, 109–121 (1969). 4303309 10.1161/01.res.24.1.109

[144] Salavatian, S. et al. Vagal stimulation targets select populations of intrinsic cardiac neurons to control neurally induced atrial fibrillation. *Am. J. Physiol. Heart Circ. Physiol.* **311**, H1311–H1320 (2016). 27591222 10.1152/ajpheart.00443.2016 PMC5130494

[145] Shin, H. C., Aggarwal, V., Acharya, S., Schieber, M. H. & Thakor, N. V. Neural decoding of finger movements using Skellam-based maximum-likelihood decoding. *IEEE Trans. Biomed. Eng.* **57**, 754–760 (2010). 19403361 10.1109/TBME.2009.2020791

[146] Wellens, H. J., Brugada, P. & Stevenson, W. G. Programmed electrical stimulation of the heart in patients with life-threatening ventricular arrhythmias: what is the significance of induced arrhythmias and what is the correct stimulation protocol? *Circulation* **72**, 1–7 (1985). 4006120 10.1161/01.cir.72.1.1

[147] Wu, C. C. et al. Enrichment of extracellular vesicles using Mag-Net for the analysis of the plasma proteome. *Nat. Commun.* **16**, 5447 (2025). 10.1038/s41467-025-60595-7 PMC12219689 40595564

[148] Stewart, H. I. et al. Parallelized acquisition of orbitrap and astral analyzers enables high-throughput quantitative analysis. *Anal. Chem.* **95**, 15656–15664 (2023). 37815927 10.1021/acs.analchem.3c02856 PMC10603608

[149] Guzman, U. H. et al. Ultra-fast label-free quantification and comprehensive proteome coverage with narrow-window data-independent acquisition. *Nat. Biotechnol.* **42**, 1855–1866 (2024). 38302753 10.1038/s41587-023-02099-7 PMC11631760

[150] Demichev, V., Messner, C. B., Vernardis, S. I., Lilley, K. S. & Ralser, M. DIA-NN: neural networks and interference correction enable deep proteome coverage in high throughput. *Nat. Methods* **17**, 41–44 (2020). 31768060 10.1038/s41592-019-0638-x PMC6949130

[151] Hsiao, Y. et al. Analysis and visualization of quantitative proteomics data using FragPipe-analyst. *J. Proteome Res.* **23**, 4303–4315 (2024). 39254081 10.1021/acs.jproteome.4c00294 PMC13142904

[152] Zimmermann, M. T., Kabat, B., Grill, D. E., Kennedy, R. B. & Poland, G. A. RITAN: rapid integration of term annotation and network resources. *PeerJ* **7**, e6994 (2019). 31355053 10.7717/peerj.6994 PMC6644632

