# Supplementary material for: Spinal nociceptive denervation impedes subsequent chronic autonomic remodeling after myocardial infarction in male swine
Source: Nat Commun. 2026 Jul 22;17:8955. doi: 10.1038/s41467-026-75759-2 (PMC13503775; doi:10.1038/s41467-026-75759-2)
Supplement: Supplementary file 2 [file 41467_2026_75759_MOESM2_ESM.pdf]

File name: Supplementary Data 1

Description: Differential proteomic analysis comparing animals that received epidural RTX prior to myocardial infarction (epidural cRTX) with animals that received vehicle prior to myocardial infarction (vehicle).
Protein ID**:** Unique protein identifier for each protein.
Gene Name**:** Gene symbol corresponding to the protein.
epidural cRTX_vs_vehicle_log2 fold change**:** Log2 fold change in protein abundance between epidural cRTX and vehicle groups. Negative values indicate lower abundance in cRTX relative to vehicle; positive values indicate higher abundance.
epidural cRTX_vs_vehicle only_p.val**:** Unadjusted p-value from the statistical test for differential abundance between groups.
epidural cRTX_vs_vehicle_p.adj**:** P-value adjusted for multiple testing using the Benjamini–Hochberg method to control the false discovery rate.
significant: indicator of significance based on the adjusted p-value threshold (FDR < 0.05).
epidural cRTX_vs_vehicle_significant**:** indicator of statistical significance for the epidural cRTX versus vehicle comparison.
imputed: Indicator of whether missing values were imputed during data preprocessing.
num_NAs**:** Number of missing values for each protein

File name: Supplementary Data 2

Description: Gene ontology enrichment analysis comparing animals that received epidural RTX prior to myocardial infarction vs. those that received epidural saline (vehicle) prior to myocardial infarction.
Name: Pathway name according to the gene ontology (GO) database
p: unadjusted p-value from the enrichment analysis
n: number of proteins inputted that were related to the GO pathway
n.set: total number of proteins annotated to the GO pathways in the background reference set
genes: Lisf of proteins that were inputted and were related to the GO pathway
q: FDR-adjusted p-value (Benjamini-Hochberg method)
GeneRatio: Ratio of input proteins mapped to the specific GO pathway relative to the total number of proteins analyzed.
