# Supplementary material for: Spinal nociceptive denervation impedes subsequent chronic autonomic remodeling after myocardial infarction in male swine
Source: Nat Commun. 2026 Jul 22;17:8955. doi: 10.1038/s41467-026-75759-2 (PMC13503775; doi:10.1038/s41467-026-75759-2)
Supplement: Supplementary file 3 [file 41467_2026_75759_MOESM5_ESM.pdf]

nature portfolio

Corresponding author(s): Marmar Vaseghi
Last updated by author(s): 6_17_2026

Reporting Summary

Nature Portfolio wishes to improve the reproducibility of the work that we publish. This form provides structure for consistency and transparency in reporting. For further information on Nature Portfolio policies, see our Editorial Policies and the Editorial Policy Checklist.

Statistics

For all statistical analyses, confirm that the following items are present in the figure legend, table legend, main text, or Methods section.

n/a	Confirmed	
☐	☒	The exact sample size (*n*) for each experimental group/condition, given as a discrete number and unit of measurement
☐	☒	A statement on whether measurements were taken from distinct samples or whether the same sample was measured repeatedly
☐	☒	The statistical test(s) used AND whether they are one- or two-sided<br>*Only common tests should be described solely by name; describe more complex techniques in the Methods section.*
☐	☒	A description of all covariates tested
☐	☒	A description of any assumptions or corrections, such as tests of normality and adjustment for multiple comparisons
☐	☒	A full description of the statistical parameters including central tendency (e.g. means) or other basic estimates (e.g. regression coefficient) AND variation (e.g. standard deviation) or associated estimates of uncertainty (e.g. confidence intervals)
☐	☒	For null hypothesis testing, the test statistic (e.g. F, t, r) with confidence intervals, effect sizes, degrees of freedom and P value noted<br>*Give P values as exact values whenever suitable.*
☒	☐	For Bayesian analysis, information on the choice of priors and Markov chain Monte Carlo settings
☒	☐	For hierarchical and complex designs, identification of the appropriate level for tests and full reporting of outcomes
☒	☐	Estimates of effect sizes (e.g. Cohen's *d*, Pearson's *r*), indicating how they were calculated

Our web collection on statistics for biologists contains articles on many of the points above.

Software and code

Policy information about availability of computer code

Data collection	Hemodynamic and intracardiac neural signals were acquired and digitized using a CED Power1401 interface with Spike2 software (Cambridge Electronic Design; v10.08). Surface 12-lead ECG and electrophysiological recordings were acquired using the GE CardioLab Recording System (GE Healthcare; v6.9.6.R1). Intracardiac echocardiography images were acquired on the ViewMate Multi Ultrasound System (Abbott). Epicardial electrogram activation and repolarization times were measured using iScaldyn (custom software, University of Utah; available from the developer on request). Confocal images were acquired on a Zeiss LSM880 using Zen 2 software (Zeiss; black edition, v3)
Data analysis	Neural spike sorting and signal analysis were performed in Spike2 (Cambridge Electronic Design; v10.08) using principal-component and K-means clustering. Activation-recovery intervals were derived from iScaldyn output (University of Utah). Proteomic raw data were processed with DIA-NN to search an in silico spectral library generated from the Sus scrofa UniProt reference proteome (ID: UP000008227). Downstream statistical analysis and normalization (random-forest normalization, two-sided Student's t-test) were conducted in R (v4.4.2). Pathway over-representation analysis was performed with RITAN (v3.20). Immunofluorescence quantification was performed in ImageJ (NIH; Fiji v1.54). Graphing and statistical testing were performed in GraphPad Prism (v10.6.1).

For manuscripts utilizing custom algorithms or software that are central to the research but not yet described in published literature, software must be made available to editors and reviewers. We strongly encourage code deposition in a community repository (e.g. GitHub). See the Nature Portfolio guidelines for submitting code & software for further information.

Data

Policy information about availability of data

All manuscripts must include a data availability statement. This statement should provide the following information, where applicable:

- Accession codes, unique identifiers, or web links for publicly available datasets
- A description of any restrictions on data availability
- For clinical datasets or third party data, please ensure that the statement adheres to our policy

The mass spectrometry proteomics data have been deposited to the ProteomeXchange Consortium via the PRIDE partner repository under accession code MV000099727 (https://doi.org/doi:10.25345/C54M91Q25). Source data underlying all figures, including individual data points, are provided with this paper in the Source Data file and Supplementary Data 1–2. Raw acquisition files (electrophysiological recordings, neural recordings, and confocal microscopy images) are large and are available from the corresponding author on request, owing to file-size and format constraints; their summary measurements are fully reported in the Source Data file

Research involving human participants, their data, or biological material

Policy information about studies with human participants or human data. See also policy information about sex, gender (identity/presentation), and sexual orientation and race, ethnicity and racism.

Reporting on sex and gender	*Use the terms sex (biological attribute) and gender (shaped by social and cultural circumstances) carefully in order to avoid confusing both terms. Indicate if findings apply to only one sex or gender; describe whether sex and gender were considered in study design; whether sex and/or gender was determined based on self-reporting or assigned and methods used. Provide in the source data disaggregated sex and gender data, where this information has been collected, and if consent has been obtained for sharing of individual-level data; provide overall numbers in this Reporting Summary. Please state if this information has not been collected. Report sex- and gender-based analyses where performed, justify reasons for lack of sex- and gender-based analysis.*
Reporting on race, ethnicity, or other socially relevant groupings	*Please specify the socially constructed or socially relevant categorization variable(s) used in your manuscript and explain why they were used. Please note that such variables should not be used as proxies for other socially constructed/relevant variables (for example, race or ethnicity should not be used as a proxy for socioeconomic status). Provide clear definitions of the relevant terms used, how they were provided (by the participants/respondents, the researchers, or third parties), and the method(s) used to classify people into the different categories (e.g. self-report, census or administrative data, social media data, etc.) Please provide details about how you controlled for confounding variables in your analyses.*
Population characteristics	*Describe the covariate-relevant population characteristics of the human research participants (e.g. age, genotypic information, past and current diagnosis and treatment categories). If you filled out the behavioural & social sciences study design questions and have nothing to add here, write "See above."*
Recruitment	*Describe how participants were recruited. Outline any potential self-selection bias or other biases that may be present and how these are likely to impact results.*
Ethics oversight	*Identify the organization(s) that approved the study protocol.*

Note that full information on the approval of the study protocol must also be provided in the manuscript.

Field-specific reporting

Please select the one below that is the best fit for your research. If you are not sure, read the appropriate sections before making your selection.

☒ Life sciences ☐ Behavioural & social sciences ☐ Ecological, evolutionary & environmental sciences

For a reference copy of the document with all sections, see nature.com/documents/nr-reporting-summary-flat.pdf

Life sciences study design

All studies must disclose on these points even when the disclosure is negative.

Sample size	No statistical method was used to predetermine sample size. Group sizes were based on our prior studies using this porcine chronic-MI model and are consistent with cohort sizes standard for large-animal cardiac electrophysiology and autonomic studies, which reliably detect the effect sizes of interest for the autonomic and electrophysiological endpoints measured here.
Data exclusions	Pre-established exclusion criteria were applied. Baroreflex sensitivity measurements were included only if the linear regression relating left ventricular systolic pressure to RR-interval had $R^2 > 0.8$. Neural recordings lost to micro- or macro-dislodgement of the recording electrode from the ganglionated plexus (during cardioversion of VT/VF or saline rinse after epicardial bradykinin/capsaicin application) were excluded from analysis. Animals that did not survive to the study endpoint were excluded from serial comparison analyses due to incomplete longitudinal data. The differing per-experiment n values reported throughout reflect these pre-specified criteria and the multi-step nature of the terminal experiments.

Replication	Findings reflect independent biological replicates (individual animals), with n stated for each experiment in the corresponding figure legends. Representative immunohistochemical images are representative of all animals analysed in the respective group (n as stated per figure). All replication attempts were successful; no findings failed to reproduce.
Randomization	Animals were randomized to the epidural RTX or vehicle treatment group prior to creation of infarcts using a random number generator in blocks of 4. There was no other formal process of randomization. The acute-RTX (aRTX) cohort underwent MI and was studied 4–6 weeks later without any randomization of animals as this was a single cohort.
Blinding	Blinding was not feasible as the investigators responsible for data acquisition and analysis also performed the epidural injections and prepared the study drugs. Histological and proteomic quantification were performed by analysts blinded to group allocation.

Reporting for specific materials, systems and methods

We require information from authors about some types of materials, experimental systems and methods used in many studies. Here, indicate whether each material, system or method listed is relevant to your study. If you are not sure if a list item applies to your research, read the appropriate section before selecting a response.

Materials & experimental systems

n/a	Involved in the study	
☐	☒	Antibodies
☒	☐	Eukaryotic cell lines
☒	☐	Palaeontology and archaeology
☐	☒	Animals and other organisms
☒	☐	Clinical data
☒	☐	Dual use research of concern
☒	☐	Plants

Methods

n/a	Involved in the study	
☒	☐	ChIP-seq
☒	☐	Flow cytometry
☒	☐	MRI-based neuroimaging

Antibodies

Antibodies used

- Goat anti-calcitonin gene-related peptide (CGRP; Abcam, ab36001)
- Mouse anti-glial fibrillary protein (GFAP; Invitrogen, ASTRO6)
- Rabbit anti-ionized calcium-binding adaptor molecule 1 (Iba1; Biocare Medical, CP 290 A, B)
- Sheep anti-tyrosine hydroxylase (TH; MilliporeSigma, AB1542)
- Rabbit anti-purine P2X3 receptor (Neuromics, RA10109)
- Rabbit anti-Piezo-type mechanosensitive ion channel component 2 (PIEZO2; ThermoFisher, PA5-72975)
- Rat anti-cluster of differentiation 3 (CD3; Abcam, ab11089).

Secondary antibodies:
- Alexa Fluor 488–donkey anti-goat IgG (Invitrogen, A-11055)
- Alexa Fluor 555–donkey anti-mouse IgG (Invitrogen, A-31570)
- Alexa Fluor 488–donkey anti-rabbit IgG (Invitrogen, A21206)
- Alexa Fluor 647–donkey anti-sheep IgG (Invitrogen, A-21448)
- CF405S–donkey anti-rat IgG (Biotium, 20419)

Validation

Where available, manufacturer-validated antibodies were used; where species-specific validation was unavailable, antibodies were selected on the basis of antigen sequence conservation and prior published data or validation of the lab in other experiments, and selectivity ssessed against published expression patterns and no-primary-antibody negative controls.

- Goat anti-CGRP (Abcam, ab36001)
This goat polyclonal antibody is validated by the manufacturer for immunohistochemistry on paraffin-embedded and frozen tissues (https://www.abcam.com/en-us/products/primary-antibodies/cgrp-antibody-ab36001). Porcine reactivity has been previously shown in various papers, including PMID: 35015733 and PMID: 31792232.

- Mouse anti-GFAP (Invitrogen, ASTRO6)
This monoclonal antibody has been validated by the manufacturer for immunohistochemistry (https://www.fishersci.nl/shop/products/gfap-monoclonal-antibody-astro6-invitrogen/11569700) as well as for the pig as one of its target species.

- Rabbit anti-Iba1 (Biocare Medical, CP290A/B)
This antibody has been validated by the manufacturer for immunohistochemistry (chrome-extension://efaidnbmnnnibpcajpcglclefindmkaj/https://biocare.net/wp-content/uploads/290.pdf).
Porcine reactivity is not explicitly documented in the manufacturer information, though prior publications have shown its applicability for porcine tissues (PMID: 28822691; PMID: 30471417)

- Sheep anti-Tyrosine Hydroxylase (MilliporeSigma, AB1542)
This antibody has been validated by the manufacturer for immunohistochemistry (https://www.sigmaaldrich.com/NL/en/product/mm/ab1542?srsltid=AfmBOorql6qkbQKDEMh2IHaFAqF9232hrrzy6yrdpkw41sgIFFzWlJJd). Porcine reactivity has been previously

	shown in multiple papers, including PMID: 41007689 and PMID: 39513933.<br><br>- Rabbit anti-P2X3 (Neuromics, RA10109)<br>The anti-P2X3 receptor antibody is validated for immunohistochemical applications (https://www.neuromics.com/RA10109?srsltid=AfmBOop3a1g8FXhA6alQnq6WxxfW6u4PNXXPUCAuABeW-sF9qCllRTpv).<br>Porcine reactivity is not explicitly documented in the manufacturer information, though a prior publication by our group has shown its applicability for porcine tissues (PMID: 35015733).<br><br>- Rabbit anti-PIEZO2 (Thermo Fisher Scientific, PA5-72975)<br>This antibody has been validated by the manufacturer for immunohistochemistry (https://www.thermofisher.com/antibody/product/PIEZO2-Antibody-Polyclonal/PA5-72975). Moreover, the manufacturer has also validated its applicability for porcine tissues.<br><br>- Rat anti-CD3 (Abcam, ab11089)<br>This antibody has been validated by the manufacturer for immunohistochemistry (https://www.abcam.com/en-us/products/primary-antibodies/cd3-antibody-cd3-12-ab11089). Porcine reactivity is not explicitly documented in the manufacturer information, though a prior publication has validated its applicability for porcine tissues (PMID: 36536461).

Animals and other research organisms

Policy information about studies involving animals; ARRIVE guidelines recommended for reporting animal research, and Sex and Gender in Research

Laboratory animals	Male Yorkshire pigs (Sus scrofa domesticus; S&S Farms) were used. Two age/weight stages were studied: MI creation at 93 ± 13 days (3-4 months) old (40.4 ± 0.7 kg) and terminal studies at 127 ± 10 days old (~4-5 months of age) (49.8 ± 0.1 kg). Healthy control animals (n=3) were used for pilot hemodynamic assessments after epidural RTX injections, these were 4-5 months old and were of 50 ±0.2 kg). Housing: 7-day acclimatisation; 16–18 °C; 12-h light/dark cycle (light 07:00–19:00); housed singly or in pairs with environmental enrichment; ad libitum water; fed LabDiet Mini-Pig chow (#55894).
Wild animals	The study did not involve wild animals.
Reporting on sex	The study used male animals only. Sex was considered in the study design; only male pigs were used to limit hormonal variability, consistent with prior work in this model. Because only one sex was studied, data could not be disaggregated by sex. This single-sex limitation is stated in the title and abstract per SAGER guidelines.
Field-collected samples	The study did not involve samples collected from the field.
Ethics oversight	All procedures were approved by the UCLA Institutional Animal Care and Use Committee (protocol #2012-106) and conformed to the NIH Guide for the Care and Use of Laboratory Animals.

Note that full information on the approval of the study protocol must also be provided in the manuscript.

Plants

Seed stocks	No seed stocks were used in the study
Novel plant genotypes	*Describe the methods by which all novel plant genotypes were produced. This includes those generated by transgenic approaches, gene editing, chemical/radiation-based mutagenesis and hybridization. For transgenic lines, describe the transformation method, the number of independent lines analyzed and the generation upon which experiments were performed. For gene-edited lines, describe the editor used, the endogenous sequence targeted for editing, the targeting guide RNA sequence (if applicable) and how the editor was applied.*
Authentication	*Describe any authentication procedures for each seed stock used or novel genotype generated. Describe any experiments used to assess the effect of a mutation and, where applicable, how potential secondary effects (e.g. second site T-DNA insertions, mosiacism, off-target gene editing) were examined.*
