# Supplementary material for: Spinal nociceptive denervation impedes subsequent chronic autonomic remodeling after myocardial infarction in male swine
Source: Nat Commun. 2026 Jul 22;17:8955. doi: 10.1038/s41467-026-75759-2 (PMC13503775; doi:10.1038/s41467-026-75759-2)
Supplement: Supplementary file 4 [file 41467_2026_75759_MOESM6_ESM.pdf]

nature portfolio

Peer Review File

Spinal nociceptive denervation impedes subsequent chronic autonomic remodeling after myocardial infarction in male swine

Corresponding Author: Dr Marmar Vaseghi

This file contains all reviewer reports in order by version, followed by all author rebuttals in order by version.

Version 0:

Reviewer comments:

Reviewer #1

(Remarks to the Author)
This study by van Weperen and colleagues uses epidural administration (C7-T1) of resiniferatoxin (RTX) in pigs to ablate sensory afferents prior to myocardial infarction, in order to test the role of sensory afferents in driving autonomic and cardiac remodeling after MI. A similar approach was used by another lab (ref 87) to look at a subset of cardiac responses after 1 hour of ischemia and 1-2 hours of reperfusion, but this study goes well beyond the earlier work. A big step toward translational relevance here is the time course, which looks at pigs 4-6 weeks after MI. Another advance here is the array of assessments done that address neural changes and inflammation in addition to a full array of cardiac function and cardiac electrophysiological responses. The authors provide convincing evidence that ablating sensory nerves through epidural RTX infusion prevents or blunts many pathological aspects of autonomic and cardiac remodeling that occur after MI. This is potentially very exciting for identifying new treatments as many disparate aspects of pathology are ameliorated. However, the manuscript is written for a cardiac electrophysiology audience rather than a general audience, and some of the importance will not be readily understood by a non-specialist. Likewise, the use of “sham” as terminology for an MI group that is essentially a vehicle control for the RTX treatment is very confusing and makes it difficult to track what is going on. I had to refer back to Figure 1 multiple times to remind myself that every animal in the study received an MI rather than a sham surgery. Please banish the word sham. Vehicle control would be more suitable and understandable by most readers.

It would be nice to see some sampling in the ventricle for nerves at the end of the mapping study. Is the distribution or neurochemical phenotype of fibers in the heart altered? There are clearly still sensory nerves in the heart, since they respond to capsaicin and bradykinin. Is this a subpopulation compared to non-RTX hearts? Do you assume the sensory fibers still in the heart are projecting through the vagus and that is why they are intact 4-6 weeks after intrathecal RTX? This should at least be discussed.

In discussing Figure 8 the authors state that HR remained elevated but LV parameters returned to near baseline. The data suggest these LV parameters were significantly lower than baseline. Please clarify. Would HR regulation differ from the ventricle because there is limited nerve loss in atria after MI?

Figure 6 needs additional labeling. It is impossible to tell what cell types the bar graphs are quantifying with the current labeling and figure legend. Instead of “number of cells” please put the cell type in the axis. “GFAP+ cells/100…” etc. and connect it to a specific panel A,B etc.

Reviewer #2

(Remarks to the Author)
In this short paper, the authors apply resiniferatoxin by epridural injection to pigs (to ablate TRV1-expressing neurons), and then conclude that the effects on myocardial infarction (MI) are the result of cardiac spinal nociceptive afferents directly contributing to MI-induced autonomic remodeling (plus an entire range of physiological alterations).

I am afraid I find the claim of a direct link between the ablation of TRV1-expressing neurons and its impact on CI tenuous at

best. In fact, I am surprised the authors would argue for a causal link given the gross manipulation (resiniferatoxin injection), without any controls for selectivity of the effects, and much less causality. For example, have they looked at the effects of the same resiniferatoxin injection on spleen function? Kidney? Liver? What about Lungs? What about changes in circulating metabolites? animal behavior? What is the evidence of a direct link between the manipulation and their observed effects in heart?

Reviewer #3

(Remarks to the Author)

A previous study (reference 87, Yamaguchi et al.) tested RTX's antiarrhythmic effects in a Yorkshire pig model. Similar to this study, those authors injected RTX into the T2-T4 spinal dorsal root, showing it mitigated ischemia-reperfusion-induced ARI shortening and dispersion of repolarization (DOR) augmentation. They showed that RTX reduced the arrhythmia score during ischemia-reperfusion and reduced CGRP expression.
In the study, epidural RTX vs. saline was administered in pigs before or after MI. The authors assessed the autonomic and electrophysiological effects. They concluded that cardiac spinal nociceptive afferents directly contribute to VT/VF susceptibility and MI-induced autonomic remodeling, including oxidative stress, inflammation, glial activation, and reduced vagal function. These studies extended the previous observation to document the antiarrhythmic effects of RTX injection in a chronic MI model. The authors provided extensive documentation to confirm that RTX injection is antiarrhythmic in the chronic MI model, and the antiarrhythmic effects were not limited to acute ischemia-reperfusion.
Comments:
1, Since Yamaguchi et al used similar experimental methods to demonstrate the antiarrhythmic effects of RTX injection, the authors should mention their findings in the Introduction. The hypothesis should be revised to say that the present study focuses on extending the previous observation to the chronic MI model.
2, Three animals were used as healthy controls to determine the safety and efficacy of RTX. The authors stated in lines 112-115 that These experiments demonstrated an initial sympathoexcitatory response after thoracic epidural injection of RTX, with increases in heart rate (HR) and left ventricular systolic pressure (LVSP), which stabilized at approximately 2 hours (Supplemental Figure 1). However, it is not what Supplemental Figure 1 showed. At the 120 min point, LVSP is still about 10% higher, and dP/dtmax is still about 20% lower than the baseline. HR returns to baseline at about 75 min, and dP/dtmin returns at about 90 min. Can the author further explain how they determine the stabilization of the hemodynamic parameters?
3, Line 116, "subsequent animals" should be defined. Are they normal animals or infarcted animals? It is a long and complicated sentence. Perhaps the authors can break it down and specify the procedures/groups of the n=11 and n=13. The words highlighted by parentheses can be a separate sentence.
4, The authors used "sympathetic nociceptive afferent signaling" in the title, but the text does not explicitly define that term. Are you talking about nerves that are both sympathetic and nociceptive? Nociceptive afferents are sensory neurons. Their cell bodies are located in the DRG and express cGRP. Sympathetic neurons are efferent neurons. They can interact, but they are separate systems. We must be cautious about using cGRP as a biomarker of sympathetic nociceptive neuronal ablation. There is also no direct evidence of sympathetic remodeling in either group in this model. Finally, does RTX kill sympathetic nerves?
5, in Ref #87, which performed a very similar study, also gave RTX to pigs' thoracic dorsal root ganglion. All animals had a dosage of 0.5 ml (100 µg/mL). In this study, the authors used 0.6-1.2 µg/kg. Given the BW of those pigs the author gave (40.4±0.7 kg), the RTX dosage varied between 24 µg and 48 µg in different animals. Could the author explain which animals received 24 and which received 48, and if the animals receiving the double dose experienced more antiarrhythmic effects?
6, In lines 510-511, MI was confirmed by lack of distal LAD flow on coronary angiography coupled with ST-segment changes. How did the authors determine the extent of myocardial infarction? Did the authors define the cardiac enzyme levels? Had the infarction area been measured? Would it be possible for different infarction sizes to influence the autonomic remodeling and the ?
7, Supplemental Figure 3: In the upper panel, it would be better to label 5/18 and 8/19 in the red boxes than in the blue boxes.
8, Discussion. The discussion is very long. It could be more focused on the mechanisms. The authors have performed a comprehensive study and documented multiple physiological, chemical, and histological changes associated with the RTX ablation. However, these observations do not necessarily indicate that they are responsible for the antiarrhythmic effects. Reference 87 showed that the same intervention is antiarrhythmic in ischemia/reperfusion-induced arrhythmia. While RTX induces changes in glial activation, inflammation, vagal dysfunction etc, are those effects needed to achieve arrhythmia control? I think the readers may appreciate some discussions on the causal relationships related to those observations.

Version 1:

Reviewer comments:

Reviewer #3

(Remarks to the Author)
no more comments.

Reviewer #4

(Remarks to the Author)
The authors have addressed the reviewers' concerns. However, as the reviewers pointed out, it shoud be empahsized that the findings are similar to Yamaguchi et al, albeit on different timescales.

Reviewer #5

(Remarks to the Author)
The revised manuscript is improved and addresses many of the prior concerns. The added histology, infarct-size/remodeling controls, and the acute post-MI RTX experiments strengthen the study and increase its potential translational relevance. Overall, the revised data support a role for cervicothoracic spinal nociceptive afferent signaling in post-MI autonomic/electrophysiological remodeling and arrhythmia susceptibility in this porcine model, although several interpretive and methodological issues remain.
a) The direct link between cardiac spinal nociceptive afferents and post-MI cardiac performance is strengthened, and the specificity of RTX manipulation is supported by the additional controls. But the evidence remains incomplete without a direct comparison of $CGRP^{+}$ fibers between RTX and vehicle groups in the kidney, spleen, and lung.
b) There are still instances of overstatement throughout the manuscript. The authors use the term “prevent” in multiple places, including the title (“Cardiac spinal 1 nociceptive afferent denervation with epidural resiniferatoxin prevents chronic structural and functional autonomic remodeling after myocardial infarction”) and a section heading (“RTX therapy prevents post-MI oxidative stress, inflammation, and glial activation”). As comparisons in the study are limited to vehicle-treated and RTX-treated MI groups, without inclusion of non-MI controls, it is difficult to assess the extent of improvement with RTX or whether the rescue is complete. The use of “prevent” is therefore potentially misleading, as it implies complete protection or recovery.
c) The authors did not characterize changes in the local immune milieu or myocardial inflammatory remodeling in the heart, which could contribute to the improved cardiac function in the RTX-treated group. This limitation should be explicitly acknowledged.
d) What is the expected unit for BRS? line 204 uses mmHg/ms, while Figure 3F states ms/mmHg.
e) In Figure 1A, the pink cohort is labeled simply as “Vehicle,” although this group are actually animals that received vehicle prior to MI. Please relabel this group as “Vehicle + MI”).

Reviewer #6

(Remarks to the Author)
I co-reviewed this manuscript with one of the reviewers who provided the listed reports. This is part of the Nature Communications initiative to facilitate training in peer review and to provide appropriate recognition for Early Career Researchers who co-review manuscripts.

Version 2:

Reviewer comments:

Reviewer #5

(Remarks to the Author)
The authors have addressed all of my concerns. I support publication of the manuscript.

Open Access This Peer Review File is licensed under a Creative Commons Attribution 4.0 International License, which permits use, sharing, adaptation, distribution and reproduction in any medium or format, as long as you give appropriate credit to the original author(s) and the source, provide a link to the Creative Commons license, and indicate if changes were made.
In cases where reviewers are anonymous, credit should be given to 'Anonymous Referee' and the source.
The images or other third party material in this Peer Review File are included in the article's Creative Commons license, unless indicated otherwise in a credit line to the material. If material is not included in the article's Creative Commons license and your intended use is not permitted by statutory regulation or exceeds the permitted use, you will need to obtain permission directly from the copyright holder.
To view a copy of this license, visit https://creativecommons.org/licenses/by/4.0/

RESPONSE TO THE REVIEWERS

Response to Reviewer 1:

Reviewer #1
This study by van Weperen and colleagues uses epidural administration (C7-T1) of resiniferatoxin (RTX) in pigs to ablate sensory afferents prior to myocardial infarction, in order to test the role of sensory afferents in driving autonomic and cardiac remodeling after MI. A similar approach was used by another lab (ref 87) to look at a subset of cardiac responses after 1 hour of ischemia and 1-2 hours of reperfusion, but this study goes well beyond the earlier work. A big step toward translational relevance here is the time course, which looks at pigs 4-6 weeks after MI. Another advance here is the array of assessments done that address neural changes and inflammation in addition to a full array of cardiac function and cardiac electrophysiological responses. The authors provide convincing evidence that ablating sensory nerves through epidural RTX infusion prevents or blunts many pathological aspects of autonomic and cardiac remodeling that occur after MI. This is potentially very exciting for identifying new treatments as many disparate aspects of pathology are ameliorated.

We are very grateful for the reviewer's thorough assessment of our manuscript and positive comments regarding the chronic effects of RTX and its translational advances.

However, the manuscript is written for a cardiac electrophysiology audience rather than a general audience, and some of the importance will not be readily understood by a non-specialist. Likewise, the use of "sham" as terminology for an MI group that is essentially a vehicle control for the RTX treatment is very confusing and makes it difficult to track what is going on. I had to refer back to Figure 1 multiple times to remind myself that every animal in the study received an MI rather than a sham surgery. Please banish the word sham. Vehicle control would be more suitable and understandable by most readers.

We thank the reviewer for this very helpful suggestion. We agree with the reviewer that the group of animals that received saline injections (instead of RTX) prior to myocardial infarction should be referred to as 'vehicle'. Therefore, we have revised the manuscript and now refer to this group of animals as 'vehicle'.

It would be nice to see some sampling in the ventricle for nerves at the end of the mapping study. Is the distribution or neurochemical phenotype of fibers in the heart altered? There are clearly still sensory nerves in the heart, since they respond to capsaicin and bradykinin. Is this a subpopulation compared to non-RTX hearts? Do you assume the sensory fibers still in the heart are projecting through the vagus and that is why they are intact 4-6 weeks after intrathecal RTX? This should at least be discussed.

We thank the reviewer for this insightful and important comment.

As correctly stated by the reviewer, the heart is indeed also innervated by mechano- and/or chemosensitive sensory nerves that project through the vagus nerve, whose cell bodies reside in the nodose or jugular vagal ganglia[1, 2]. Cardiac chemosensitive vagal afferents constitute a heterogeneous group of neurons, which can be activated through a variety of chemicals, including bradykinin, prostaglandins, ATP, and reactive oxygen species[3-5], though a significant portion of chemosensitive cardiac vagal afferents are C-type nociceptive fibers and express the transient receptor potential vanilloid-1 (TRPV1) receptor[6-8]. In the present study we administered resiniferatoxin (RTX), an ultrapotent analog of the TRPV1 receptor [9-13], in the epidural space. RTX has been previously shown to initially activate (by opening calcium channels) and then ablate (via cytotoxicity) specifically TRPV1 positive nociceptive, chemosensitive neurons through a rapid and prolonged influx of calcium ions[14-17]. Hence, cervicothoracic (C7-T1) epidural administration of this drug, which targets the spinal TRPV1 nociceptive afferent fibers, will not have affected cardiac vagal TRPV1-positive afferents, as they don't pass through the dorsal root ganglia or the epidural space. Moreover, as RTX is specific for TRPV1 expressing neurons, both vagal and spinal TRPV1 negative chemosensory and mechanosensitive afferents would not be affected.

To further support the above statements and in light of the reviewer's insightful comment, we have now performed additional experiments to assess the phenotype of fibers in the ventricular epicardial

myocardium, specifically focusing on tyrosine hydroxylase (TH) expressing fibers (labeling dopaminergic afferents and sympathetic efferents), and calcitonin gene-related peptide (CGRP) expressing afferents (which would target the TRPV1 expressing fibers), as proposed by the reviewer. As can be appreciated from Figure 2, cervicothoracic epidural RTX did not alter the ventricular myocardial expression of TH-expressing fibers but did significantly decrease the number of CGRP-expressing fibers. Hence, we show that cervicothoracic epidural RTX, by ablating spinal CGRP+ afferents fibers, reduces the expression of these fibers in the myocardium, while the expression of sympathetic efferent and/or dopaminergic fibers is unchanged. Notably, there was still some residual presence of CGRP-expressing fibers in the ventricular myocardium of RTX treated animals, consistent with the fact that vagal TRPV1-expressing afferents are not affected by the epidural administration of RTX.

As the reviewer correctly notes, these vagal sensory nerves, that were not affected by epidural RTX administration, are likely responsible for the hemodynamic and electrophysiological responses that were observed in response to epicardial administration of capsaicin and bradykinin. As capsaicin is an agonist of the TRPV1 receptor as well, the vagal response that is observed upon its epicardial administration correlates well with the activation of vagal TRPV1 positive chemosensory afferents, whereas the spinal-sympathetic reflex upon activation of spinal TRPV1 positive afferents is significantly reduced by their ablation.

Bradykinin on the other hand, predominantly signals through the B2 receptor[18, 19], which has been shown to, at least in part, signal through activation of TRPV1 positive neurons [18, 20-22]. Hence, also in response to bradykinin, a predominant vagal response is observed in RTX treated vs. vehicle animals, as the spinal reflexes are engaged to much lesser extent due to the ablation of spinal TRPV1 chemosensitive afferents. As vagal chemosensitive afferents, both TRPV1 positive and negative ones, were unaffected, vagal response, as demonstrated by bradycardia and decrease in blood pressure, dominated the hemodynamic and electrophysiological response to epicardial bradykinin application in RTX treated animals.

In comparison, in a prior study where epicardial administration of RTX was utilized, which would ablate both spinal and vagal TRPV1 positive afferents, no hemodynamic changes in response to epicardial capsaicin or bradykinin were observed[23].

Moreover, we have now stained for and analyzed the immunoreactivity of two other primary groups of afferent neurons: P2RX3- and PIEZO2-expressing neurons (nociceptive purinergic and mechanosensitive afferents that do not significantly express TRPV1) in cervicothoracic spinal cords and DRG of vehicle-treated animals *versus* cRTX animals, to further validate the specificity of RTX (Figure 2). We now show new data that there is no difference in P2RX3 or PIEZO2 expressing neurons in the spinal cord or DRGS between chronically infarcted, vehicle-treated versus cRTX animals.
We have since added the following sentences to the discussion to better explain these findings.

Page 7, Lines 160-182

"Although prior literature has shown selectivity of RTX for TRPV1-expressing chemosensitive neurons[9-13], we further confirmed the selectivity of RTX in our studies by evaluating two other major afferent populations: the chemosensitive purinergic P2RX3-expressing neurons and mechanosensitive PIEZO2-expressing neurons. Consistent with prior literature, evaluation of the C7-T1 spinal cord DRG and dorsal horns showed that the percentage of PIEZO2- and P2RX3-immunoreactive afferents was similar between cRTX and vehicle-treated animals (Figure 2E-H).

It has been shown that given the fatty nature of the epidural space, injection of anesthetics and chemical at various spinal levels, including RTX, rarely diffuse beyond 1-2 spinal levels[24-26]. However, to further confirm the anatomical and spatial selectivity of C7-T1 epidural RTX in our chronically infarcted animals, CGRP expression in the spinal cord dorsal horn and DRG at the cervicothoracic level (C7-T1) were compared to the thoracolumbar level (T12-L1). In accordance with other reports, we observed that CGRP expression was unchanged in the T12-L1 dorsal horn and DRG of vehicle-treated versus cRTX animals (Figure 2I-J).

Finally, as cardiac spinal C7-T1 afferents chiefly innervate the heart[27], we evaluated whether ablation of nociceptive neurons at this level with RTX would manifest as a loss of CGRP-expressing fibers at the level of the ventricular myocardium. Evaluation of LV myocardium showed that the

density CGRP-expressing ventricular fibers was reduced in epidural cRTX versus vehicle treated animals (Figure 2K-M). By contrast, the abundance of tyrosine hydroxylase (TH), a marker of sympathetic efferent fibers, was unchanged, further underscoring the chemical selectivity of RTX for TRPV1-nociceptive neurons."

<u>Page 17, Lines 403-405</u>

"The decrease in DoR is also consistent with the potentially cardioprotective effects of vagal TRPV1-positive afferents[28, 29] that do not pass through the dorsal root ganglia or the epidural space, and therefore, remained intact."

Figure 2

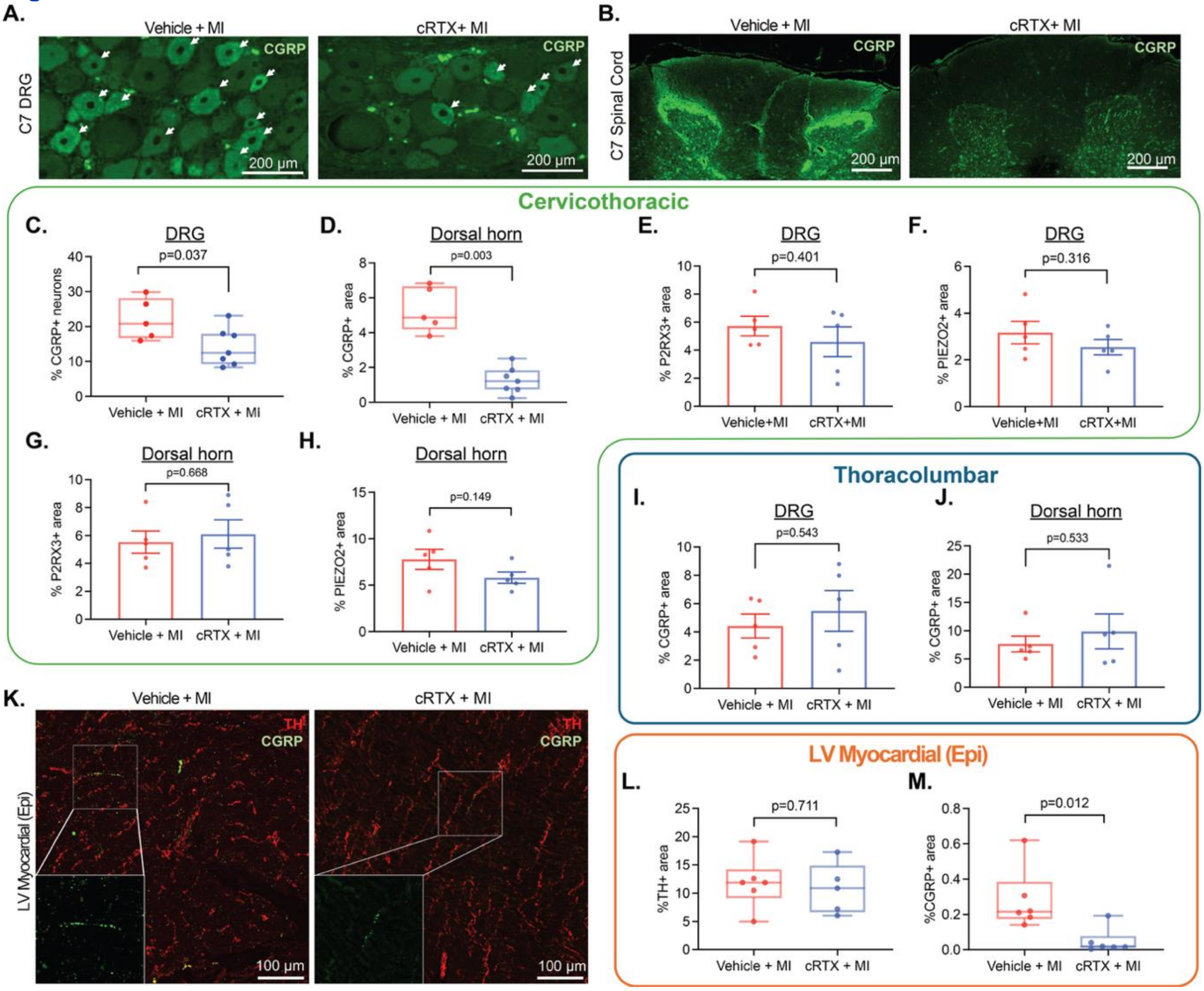

In discussing Figure 8 the authors state that HR remained elevated, but LV parameters returned to near baseline. The data suggest these LV parameters were significantly lower than baseline. Please clarify. Would HR regulation differ from the ventricle because there is limited nerve loss in atria after MI?

We appreciate this comment from the reviewer with regard to the hemodynamic parameters. We have now clarified the wording in the manuscript to state that LV parameters stabilized but remained lower than baseline values. The reviewer also raises several interesting points regarding the mechanism behind these findings. While it's possible that the results are due, in part, to limited nerve loss in atria following MI, it's also possible that ablation of spinal, chemosensitive TRPV1 positive afferents in animals with a chronic myocardial infarction interrupted the cycle of sympathetic efferent activation that is perpetuated by the continuous activation these afferents, resulting in a lower blood pressure and inotropy. In addition, as epidural RTX only ablates chemosensitive TRPV1 positive afferents, it's possible that as spinal mechanosensitive afferents remain unaffected, that these mechanosensitive

afferents, in response to the sensed decreases in blood pressure and inotropy, can initiated a reflex to increase heart rate. Without ablating these mechanosensitive afferents, it would be difficult to know what caused the persistence of the mild increase in heart rate.

We have since rewritten these sentences to better reflect the hemodynamic observations:

> <u>Page 14, Lines 328-333:</u>
> *"Consistent with animals receiving RTX prior to MI (cRTX), aRTX induced an initial sympathoexcitatory response with peak increases in HR (84±4 to 87±4 beats/min, p=0.001), LVSP (118±5 to 131±7 mmHg, p<0.01), and inotropy (1393±67 to 1484±79 mmHg/s, p<0.01) within the first hour, Figure 8. By 4 hours after epidural RTX administration, HR had stabilized but remained higher than pre-RTX values, while LV inotropy and LVSP were slightly lower (Figure 8A-E)."*

Figure 6 needs additional labeling. It is impossible to tell what cell types the bar graphs are quantifying with the current labeling and figure legend. Instead of "number of cells" please put the cell type in the axis. "GFAP+ cells/100…" etc. and connect it to a specific panel A,B etc.

Thank you for this very helpful comment. We have now revised the figure panels and labels in Figure 6 to better display the findings.

Figure 6

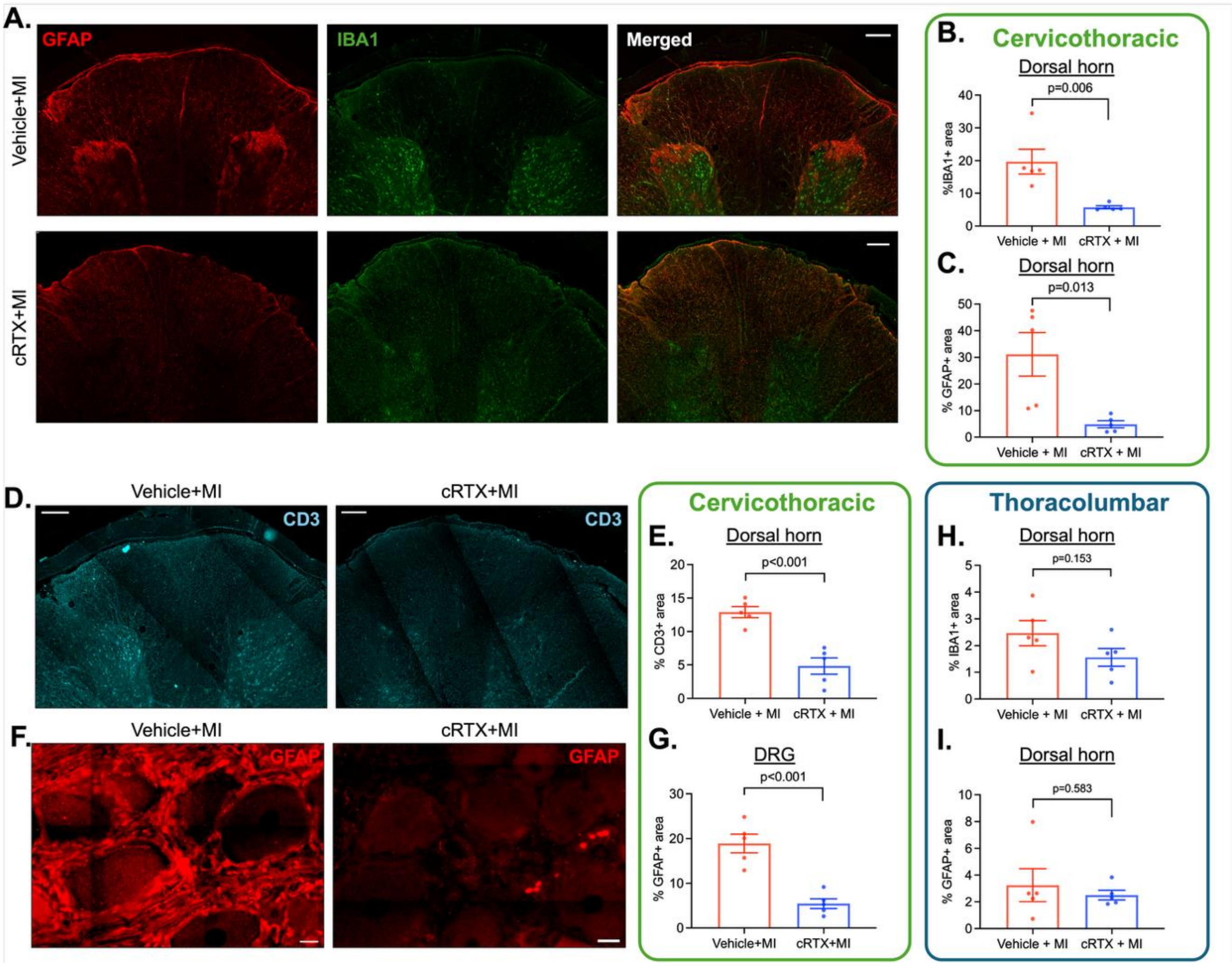

Response to Reviewer 2:

Reviewer #2 (Remarks to the Author):
In this short paper, the authors apply resiniferatoxin by epidural injection to pigs (to ablate TRV1-expressing neurons), and then conclude that the effects on myocardial infarction (MI) are the result of cardiac spinal nociceptive afferents directly contributing to MI-induced autonomic remodeling (plus an entire range of physiological alterations).

I am afraid I find the claim of a direct link between the ablation of TRV1-expressing neurons and its impact on CI tenuous at best. In fact, I am surprised the authors would argue for a causal link given the gross manipulation (resiniferatoxin injection), without any controls for selectivity of the effects, and much less causality. For example, have they looked at the effects of the same resiniferatoxin injection on spleen function? Kidney? Liver? What about Lungs? What about changes in circulating metabolites? animal behavior? What is the evidence of a direct link between the manipulation and their observed effects in heart?

We thank the reviewer for his/her evaluation of our manuscript. We acknowledge that additional data and references would have strengthened the mechanistic basis of the study, suggesting the role of cardiac spinal TRPV1+ afferents in contributing to autonomic remodeling and ventricular arrhythmias. We have now performed additional analyses and also provided a more detailed description of the effects of RTX to further support the conclusions and the reviewer's concerns.

In our study, resiniferatoxin (RTX) or vehicle (saline) was administered epidurally, at the C7-T1 cervicothoracic level in animals undergoing a myocardial infarction (MI), and effects evaluated 4-6 weeks after MI. In addition, in a separate group of animals, we evaluated the acute effects of C7-T1 epidural RTX (aRTX) in animals with established MI (4-6 weeks after MI). RTX has been extensively evaluated in prior studies and has been shown to be a selective ultrapotent agonist of the transient receptor potential vanilloid-1 (TRPV1) positive C-type nociceptors[9-13]. TRPV1 functions as a ligand-gated cation channel with enables the influx of calcium and sodium ions[30], with a selectivity for Ca^{2+} over Na^{+}[31]. Through the activation of these TRPV1 nociceptive afferents, a variety of noxious stimuli, including temperature/heat, changes in pH and exposure to toxins can be transmitted[32-37]. Hence, outside of the cardiac field, RTX and more specifically, localized epidural RTX have been extensively studied and used for nociceptive blockade of TRPV1 afferents for chronic pain syndromes and in pain associated with cancer, including in clinical, placebo-controlled, clinical trials[24, 38-41].

Compared with other TRPV1 agonists, such as capsaicin, which induce a rapid, burst-like influx of calcium[42], neuronal application of RTX has been demonstrated to cause a slower but sustained increase in intracellular calcium influx, resulting in calcium toxicity and death of neurons which express TRPV1[9, 14, 16, 17, 43, 44]. Of note, in TRPV1 expressing neurons, activation of this receptor releases CGRP, and hence CGRP has been used a marker for the presence of TRPV1 neurons, especially in larger animals[45, 46].

Hence, in the present study, the epidural administration of RTX will have only ablated TRPV1 expressing nociceptive spinal neurons as RTX is a specific agonist for this receptor. The epidural administration of this drug will not have affected cardiac vagal TRPV1-positive afferents, as they do not pass through the dorsal root ganglia or the epidural space. Moreover, due to its high specificity for TRPV1 neurons, vagal and spinal TRPV1 negative chemosensory and mechanosensitive afferents (those that do not express TRPV1[47, 48]) would not be affected.

Nevertheless, to further demonstrate the specificity of RTX in our study, we have now evaluated differences in two other major populations of neurons/fibers that express little to no TRPV1 in RTX-treated vehicle- treated infarcted animals (chemosensitive purinergic P2RX3 expressing neurons and mechanosensitive PIEZO2 expressing neurons). These data show that in the cervicothoracic (C7-T1) spinal cord and DRGs, the presence of PIEZO2 and P2RX3 neurons/fibers is unchanged in cRTX vs. vehicle treated animals, 4-6 weeks post-MI. Based on this data, the following has been added to the Results section and this new data shown in figure 2:

<u>Page 7, Lines 160-166:</u>

"Although prior literature has shown selectivity of RTX for TRPV1-expressing chemosensitive neurons[9-13], we further confirmed the selectivity of RTX in our studies by evaluating two other major afferent populations: chemosensitive purinergic P2RX3-expressing neurons and mechanosensitive PIEZO2-expressing neurons. Consistent with prior literature, evaluation of the C7-T1 spinal cord DRG and dorsal horns showed that the percentage of PIEZO2- and P2RX3-immunoreactive afferents was similar between cRTX and vehicle-treated animals (Figure 2E-H)."

<u>Page 7, Lines 167-174</u>

"It has been shown that given the fatty nature of the epidural space, injection of anesthetics and chemical at various spinal levels, including RTX, rarely diffuse beyond 1-2 spinal levels[24-26]. However, to further confirm the anatomical and spatial selectivity of C7-T1 epidural RTX in our chronically infarcted animals, CGRP expression in the spinal cord dorsal horn and DRG at the cervicothoracic level (C7-T1) were compared to the thoracolumbar level (T12-L1). In accordance with other reports, we observed that CGRP expression was unchanged in the T12-L1 dorsal horn and DRG of vehicle-treated versus cRTX animals (Figure 2I-J)."

Figure 2

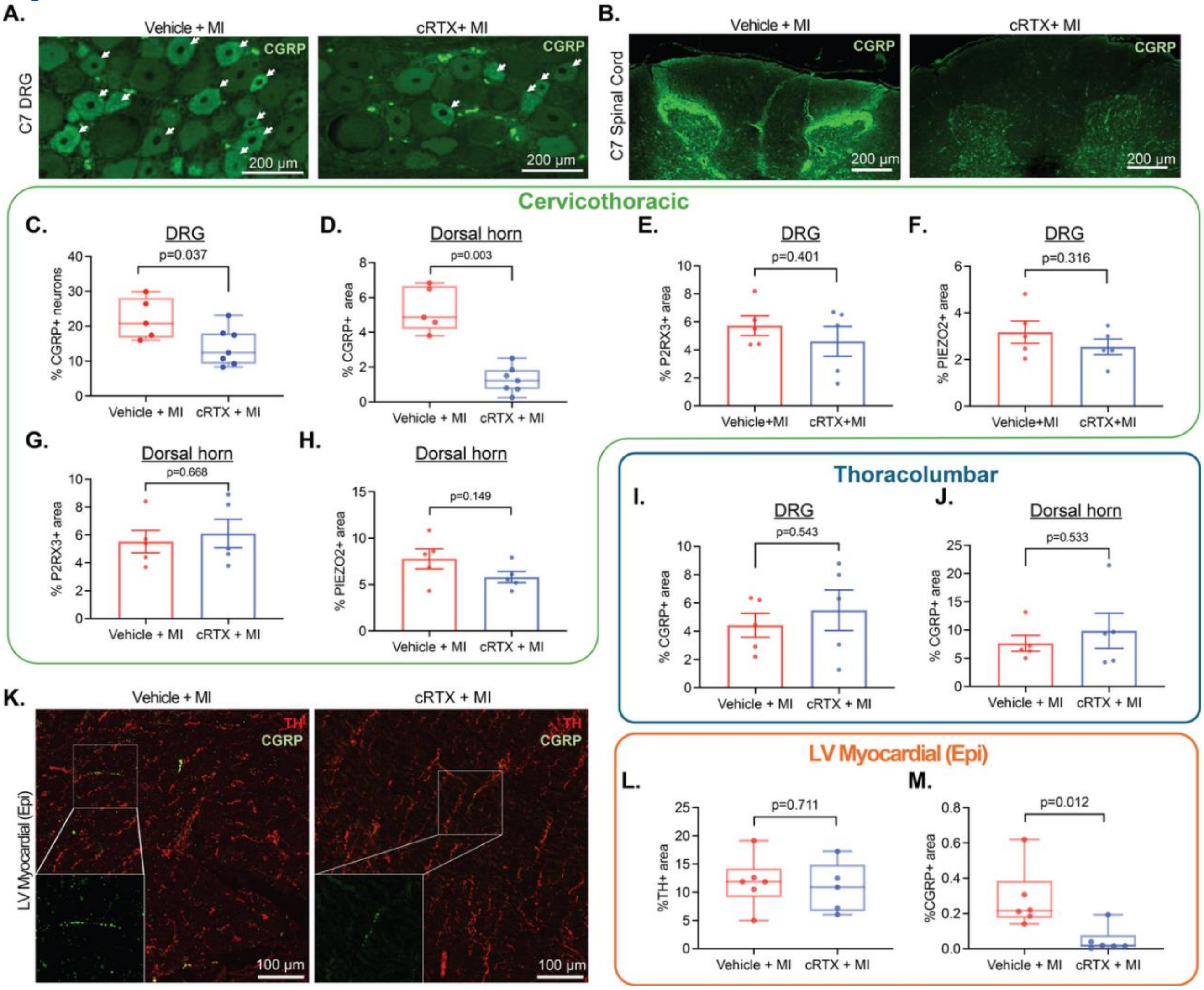

With regards to the other potential organs that can be affected by epidural RTX, we discuss each organ below.

<u>Lungs</u>

We do agree with the reviewer that it's possible that RTX may have affected the lungs. We did not specifically check the lung tissue for TRPV1 afferent denervation, because of prior literature had demonstrated that the lungs are not significantly innervated by *spinal* TRPV1 afferents. While the lungs

are densely innervated by sensory TRPV1 afferent nerves, the vast majority of these nerves run through the vagal trunk [49-52], and are important for the control of respiration rate, airway defense, and cough[53-57]. There is very limited spinal TRPV1 afferent innervation of the lungs originating in the C7-T4 spinal cord/dorsal root ganglia (DRG)[49, 58, 59]. The limited presence of spinal TRPV1 sensory afferents in the lung has been confirmed by Kim et al. (2022)[60], who quantified the innervation of the mouse lung by vagal and spinal sensory subsets defined by the expression of Pirt (all afferents), 5HT3 (vagal nodose afferents), Tac1 (tachykinergic afferents), and transient receptor potential vanilloid 1 channel (TRPV1; nociceptive afferents) using Cre-mediated reporter expression. In their study, they showed that whereas vagal afferent fibers innervate almost all conducting airways and project into the alveolar regions, DRG (spinal) afferents only innervate large airways. Moreover, of these DRG/spinal afferents, only 2% express TRPV1[60].

Very little is known about the physiological role of these TRPV1 spinal afferents in the lung, which represent a very small percentage of all TRPV1 afferent innervation of the lungs, and studies exploring the physiological effects of their activation have demonstrated opposing effects on cardiovascular function[59, 61-65]. With regards to pulmonary function, activation of these spinal TRPV1 afferents appears to have limited physiological consequences, as it induces relaxation of large airway smooth muscle[59, 61-66]. Therefore, we believe that the ablation of these spinal, TRPV1-expressing pulmonary afferents in our study would have had very limited effects on our outcome parameters. In addition, in our three times a day of monitoring of animals in the first 3 days post-op and daily monitoring of animals for the remaining 4-6 weeks post-op, no differences in respiratory rate or dyspnea were noted in the cRTX vs. vehicle-treated chronic MI animals.

Liver

The liver is innervated by both vagal and spinal afferent neurons[67-69]; these spinal afferents originate from the bilateral T7-T11 DRG[70-72]. Though no formal studies have looked at spinal TRPV1 innervation of the liver, several studies have evaluated CGRP expressing nociceptive innervation of the liver[68, 69]. In these rat studies, CGRP expressing fibers were restricted to the portal tracts[68, 69]. Similarly, in human studies, no CGRP expressing fibers were found innervating the hepatic lobules[73]. Human spinal afferent innervation in general, as assessed through labeling of substance P, is limited to the portal space[74]. Therefore, there is little to no evidence that spinal nociceptive TRPV1 expressing neurons innervate the hepatic lobules and hepatocytes to alter metabolism or liver function, in order to account for the results of the current study. In addition, as demonstrated by Figure 2 panel I-J, and in line with a prior study by Shanks et al. (2020)[75] epidural administration of RTX in rats, due to the fatty nature of the epidural space, only allows for minimal diffusion of the drug, and therefore a C7-T1 administration would have been unlikely to diffuse to T7 to T11 (levels which innervate the liver), especially since pigs are not upright animals. Lastly, we have now performed additional analyses and obtained new data comparing plasma levels of aspartate aminotransferase (AST) and aminotransferase (ALT), which are known to be released by the liver in case of hepatic dysfunction (Supplemental figure 4). Both AST and ALT levels were comparable between epidural cRTX and vehicle-treated animals, further supporting that cervicothoracic epidural RTX was unlikely to affect hepatic function and the outcomes of our present study.

Page 8, Line 188-193

Furthermore, plasma levels of hepatic enzymes aspartate transaminase (AST) and alanine (ALT), which are clinically used to assess liver function, were similar between RTX-and vehicle-treated animals (Supplementary Figure 5)."

Supplemental Figure 4

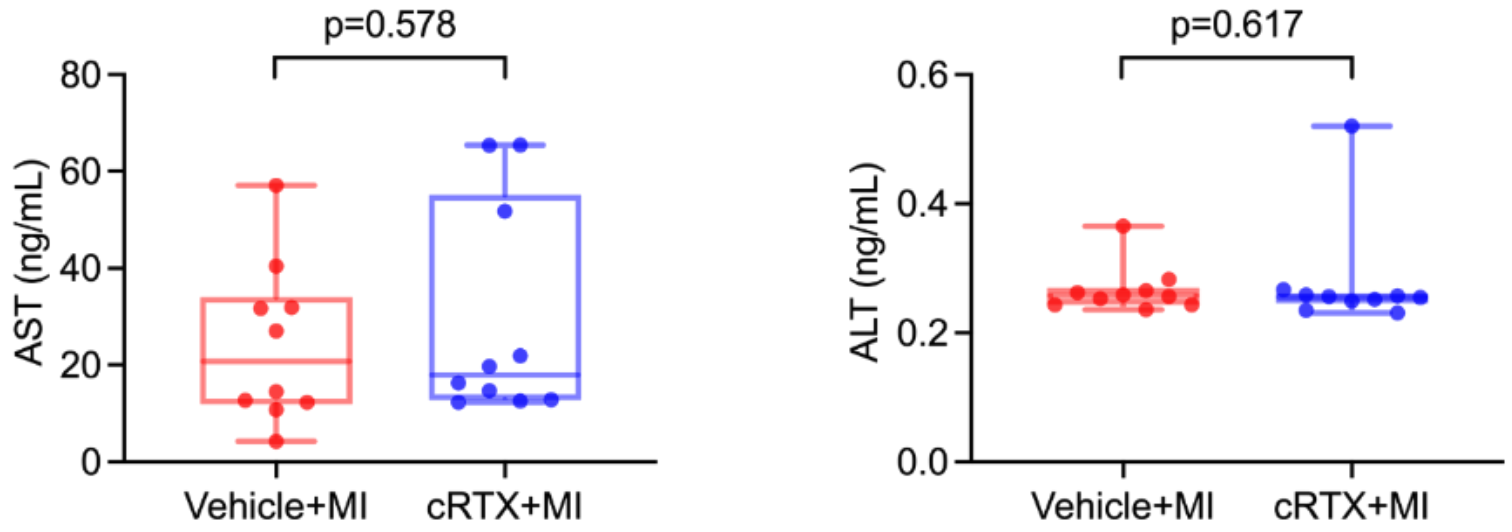

Spleen
A few studies have evaluated the sensory innervation of the spleen, and more specifically, splenic innervation by spinal TRPV1 sensory afferents. A recent study by Wu et al. (2024)[76], using whole-tissue clearing, demonstrated that spinal innervation of the spleen originates from left-sided T8–T13 DRGs in mice. Next, this study ablated spinal TRPV1 sensory afferents by intraganglionic dorsal root ganglia RTX injections (left T8-T13 DRGs), which led to decreased humoral immune response after antigen immunization (though splenic weight was not affected). Hence, spinal TRPV1+ nociceptive neurons in left T8–T12 DRGs appear to play a role in immune-response in mice. Given prior data showing limited diffusion of RTX, it is unlikely that RTX affected these lower thoracic spinal levels and DRG in our study. In our study, we observed both in our newly performed plasma studies evaluating cytokines that are known to be elevated after myocardial infarction (supplementary figure 6) as well as in our proteomics analyses, that inflammation and inflammatory pathways were reduced by administration of C7-T1 epidural RTX. However, it's possible that these immunomodulatory changes may actually be driven by the increased vagal tone that resulted from C7-T1 administration of RTX and was noted in our study. Vagal tone significantly modulates inflammation, and vagal activation has been shown to reduce cytokines and immune markers of inflammation[77, 78]. Vagal nerves stimulation is being considered as a therapy for chronic inflammatory conditions, including inflammatory bowel disease and rheumatoid arthritis[79-82]. Hence, it's possible that cRTX at C7-T1, by removing the spinal/sympathetic inhibition of central vagal drive, improves vagal function to the spleen and reduces inflammation via the vagal anti-inflammatory reflex[83]. Elevated vagal tone, by releasing acetylcholine that binds to nicotinic receptors on white blood cells in the spleen, reduces the release of cytokines and inflammation[84-86]. The plasma data has now been added to the manuscript and the possible immunomodulatory effects further discussed in the Discussion.

Page 10-11, Lines 247-249:
"Thus, to assess if spinal nociceptive afferent signaling may also contribute to this post-MI inflammatory phenotype, we assayed differences in the plasma proteome by ELISA (Supplemental Figure 6)…"

Supplemental Figure 6

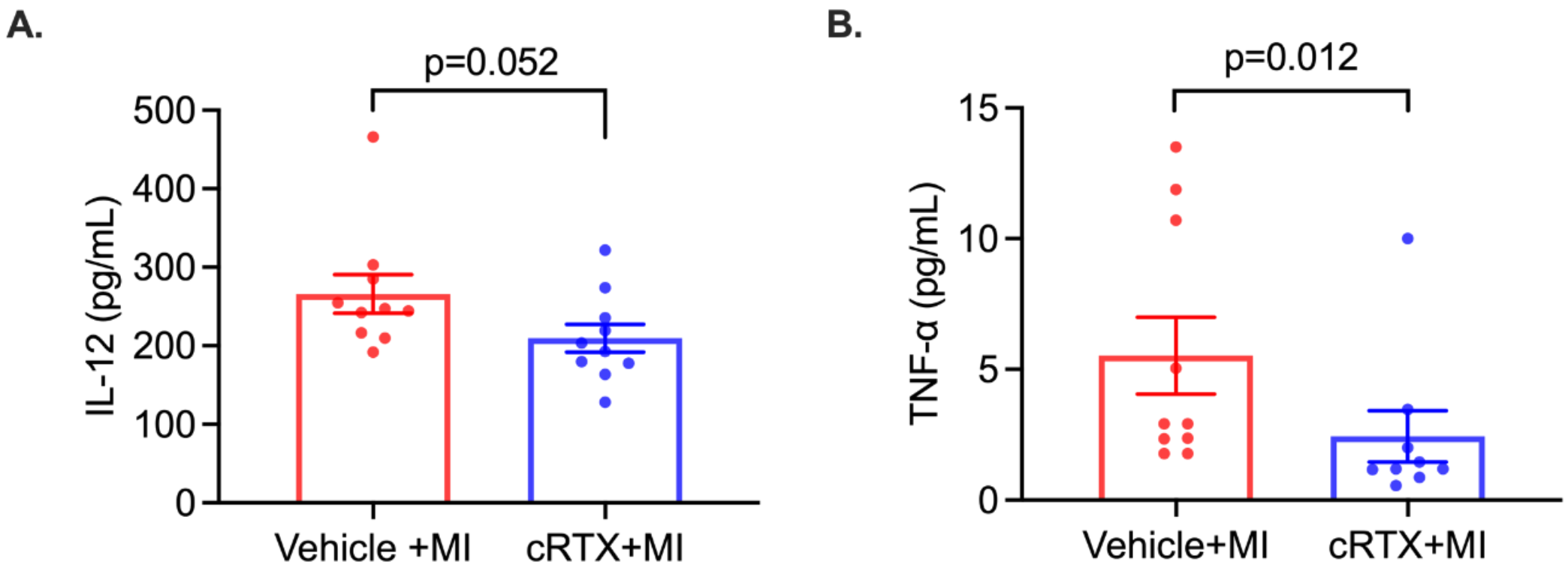

And the following has been added to the Discussion:

Page 18, Lines 436-441
"It is thus possible that spinal nociceptive afferent signaling may contribute to the heightened neuroinflammatory milieu observed after MI. Furthermore, beyond reducing local neuroinflammation, the improved vagal tone after RTX treatment may also engage the splenic cholinergic anti-inflammatory reflex110, where acetylcholine binds to nicotinic receptors on white blood cells, to attenuate systemic cytokine release and systemic inflammation[84-86]."

Kidneys
Spinal afferent innervation of the kidneys originates in the T10-L3 bilateral DRGs[87-89]. As neurohormonal activation initiated by the kidneys and renal afferents can play an important role in post-MI remodeling, we performed additional immunohistochemical studies to evaluate whether RTX had caused denervation of CGRP fibers in the thoracolumbar spinal cord and DRGs. These studies showed

that lower thoracic and higher lumbar DRGs (T12-L1) and T12-L1 spinal cord were not affected by the C7-T1 epidural RTX injection in our study, with no changes in CGRP immunoreactivity between vehicle and RTX-treated animals observed. This data has been added to figure 2 (panel I-J, please also see above). Therefore, it is less likely that the administration of cervicothoracic epidural RTX affected renal spinal afferent signaling. As can be appreciated from these results (Figure 2), RTX successfully ablated CGRP in the C7-T1 region, but not in the lower thoracic/lumbar regions, further confirming the spatial specificity of RTX.

Similarly, in the study by Shanks et al. (2020)[75] epidural administration of RTX, due to the fatty nature of the epidural space, resulted in minimal diffusion of the drug to other spinal levels. In their rat model, RTX at most, spread two segments cranial or caudal. This study also showed that thoracic ablation of TRPV1 neurons affected blood pressure in spontaneous hypertensive rats, but that ablation of lumbar (renal) TRPV1 nociceptive neurons did not affect blood pressure. Given our new analyses and prior studies, it is less likely that cervicothoracic epidural RTX could directly affect renal function by affecting the DRGs/spinal levels innervating the kidneys.

<u>Behavioral changes and weight gain</u>
We appreciate the thoughtful suggestion by the reviewer that epidural administration of RTX might have behavioral effects. In response to the reviewer's comments, we have reevaluated our detailed animals' files and logs as well as those of veterinary staff, which note the activity, food and water intake, respiration rate and behavioral changes daily during the 4 to 6 weeks between the survival (epidural RTX or vehicle injection and MI creation) and follow up terminal studies. In these reports, no behavioral changes/differences were noted in RTX- or vehicle- treated infarcted animals for the 4 to 6 weeks following RTX injections and MI induction. Notably, weight gain was similar in both groups. Moreover, prior studies[15] that administered RTX intrathecally, a technique that is more likely to cause a wider spread of the drug to intracerebral regions and/or affect the CNS more liberally, also noted no behavioral effects of RTX. Only at the very high doses (3.6 μg/kg) was one animal noted to show potential signs of decreased movement (*vs.* 1.2 μg/kg given in the epidural space in our study).

Thanks for the reviewer's important comment, the following has been added to the manuscript:

<u>Page 8, Lines 185-193:</u>
"In concordance with the histological data indicating segmental selectivity in the effects of RTX, local epidural administration of RTX did not appear to cause any significant off-target effects, as no behavioral differences were noted between cRTX and vehicle-treated animals in the 4-6 weeks following MI. Furthermore, plasma levels of hepatic enzymes aspartate transaminase (AST) and alanine (ALT), which are clinically used to assess liver function, were similar between RTX-and vehicle-treated animals (Supplementary Figure 4). Finally, the degree of weight gain in the four-to-six-week period following vehicle or RTX treatment was similar between these groups (Supplementary Figure 5)."

Supplemental Figure 5

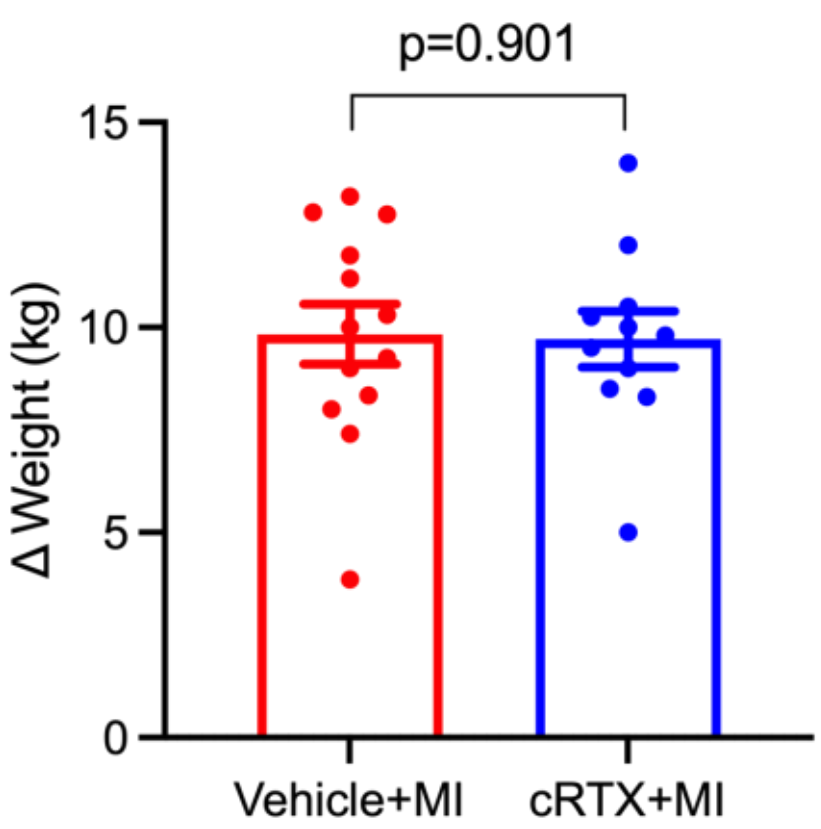

Despite our new experiments and analyses and review of prior literature reporting the selectivity and limited diffusion of epidural RTX and the very limited TRPV1 innervation of the lungs by spinal afferents, we agree that it would be impossible to completely rule out residual effects on other visceral organs,

especially those innervated by the cervicothoracic DRGs (C7-T3). Therefore, we have also added the following to the limitation section of the manuscript.

Page 21-22, Lines 513-527

"While RTX was administered at the C7-T1 levels only, effects on other organ systems cannot be completely ruled out. Nevertheless, as renal[87-89], splenic[76], and hepatic[70-72] spinal afferents originate from low thoracic/lumbar levels, and prior work indicates limited epidural spread of chemicals/molecules[24-26] (including epidural RTX), it is unlikely that these organs were affected significantly by the RTX administered at the cervicothoracic level. Consistent with this, plasma aspartate transaminase (AST) and alanine (ALT), clinically indices of liver health, were similar between cRTX and vehicle-treated animals. Although pulmonary spinal afferents run through similar thoracic levels as cardiac spinal afferents, pulmonary innervation by spinal TRPV1-expressing nociceptive neurons is sparse[60], and largely confined to large airways. Furthermore, activation of these pulmonary afferents has been shown to result in large airway smooth muscles relaxation,[59, 61-66] and therefore, unlikely to account for the cardiac electrophysiologic and autonomic results observed in this study. Finally, we did not observe effects on the T12-L1 spinal cord/DRG that innervate the kidneys to suggest that the observed results are mediated by effects on renal afferent innervation."

In addition, the discussion has been largely rewritten to better highlight the link between cardiac spinal afferent signaling and MI-induced autonomic and electrophysiological remodeling.

Response to Reviewer 3:

Reviewer #3 (Remarks to the Author):

A previous study (reference 87, Yamaguchi et al.) tested RTX's antiarrhythmic effects in a Yorkshire pig model. Similar to this study, those authors injected RTX into the T2-T4 spinal dorsal root, showing it mitigated ischemia-reperfusion-induced ARI shortening and dispersion of repolarization (DOR) augmentation. They showed that RTX reduced the arrhythmia score during ischemia-reperfusion and reduced CGRP expression.
In the study, epidural RTX vs. saline was administered in pigs before or after MI. The authors assessed the autonomic and electrophysiological effects. They concluded that cardiac spinal nociceptive afferents directly contribute to VT/VF susceptibility and MI-induced autonomic remodeling, including oxidative stress, inflammation, glial activation, and reduced vagal function. These studies extended the previous observation to document the antiarrhythmic effects of RTX injection in a chronic MI model. The authors provided extensive documentation to confirm that RTX injection is antiarrhythmic in the chronic MI model, and the antiarrhythmic effects were not limited to acute ischemia-reperfusion.

We appreciate the detailed assessment of our manuscript by reviewer 3 and are grateful for the constructive comments that have helped improve the manuscript.

Comments:
1, Since Yamaguchi et al used similar experimental methods to demonstrate the antiarrhythmic effects of RTX injection, the authors should mention their findings in the Introduction. The hypothesis should be revised to say that the present study focuses on extending the previous observation to the chronic MI model.

We are grateful for the suggestion to better highlight the differences between our study and the previous study by Yamaguchi et al. As such, the following sentences have been added to the introduction and the discussion:

Page 4, Lines 90-109

"Consistent with this, a prior study showed that the ablation of cardiac TRPV1 neurons via direct application of RTX on thoracic dorsal root ganglia (DRG) reduced acute ischemia driven ventricular arrhythmias in a healthy porcine model[90]. Yet the effect of spinal afferent interruptions with RTX on ventricular arrhythmias has not been evaluated beyond the acute MI window, even

though ventricular tachyarrhythmias due to chronic autonomic and myocardial remodeling remain an important cause of SCD long after the acute event[91-93]. Hence, in this study, we first sought to assess if chronic spinal nociceptive ablation is an important contributor to the ensuing autonomic remodeling that leads to electrophysiological heterogeneity and ventricular arrhythmias with MI. To accomplish this, we administered epidural RTX prior to MI at the C7-T1 levels to target cardiac spinal TRPV1 nociceptive afferent fibers and neurons. Four-to-six weeks after chronic MI, we evaluated (i) tissue structural and neurochemical changes, (ii) functional autonomic responses, and (iii) electrophysiological stability in chronically infarcted pigs which received RTX versus vehicle. We next sought to evaluate the potential therapeutic value of spinal nociceptive ablation for patients with refractory VT/VF after the acute phase of MI, when chronic pathological autonomic remodeling has already developed. To that end, we tested whether acute C7-T1 epidural RTX ablation of spinal nociceptive afferents in the established post-MI setting (4-6 weeks post-MI) would be sufficient to improve autonomic balance and reduce VT/VF burden."

<u>Page 19-20, Lines 464-477:</u>

"A prior study in pigs without a myocardial infarction assessed the effects of T2-T4 DRG RTX application 30 minutes prior to induction of ischemia on VT/VF burden during the acute ischemia and one hour reperfusion period[90]. In light of that report and the observed, longitudinal effects of RTX prior to MI reported herein, we subsequently aimed to evaluate the effects C7-T1 afferent ablation acutely four-to-six weeks after MI. Acute epidural RTX administration in chronically infarcted pigs was well tolerated, with no observed hemodynamic instability. Within 4 hours of administration, we observed improvements in vagal BRS, increased ventricular refractory period, attenuated pacing-induced dispersion of repolarization, and lower VT/VF inducibility. Taken together, these data suggest that, in the setting of chronic MI, cardiac nociceptive spinal afferents, perhaps by increasing efferent outflow, are key contributors to the maintenance of pathological vagal dysfunction and cardiac electrical instability, thereby supporting their role in VT/VF susceptibility."

2, Three animals were used as healthy controls to determine the safety and efficacy of RTX. The authors stated in lines 112-115 that These experiments demonstrated an initial sympathoexcitatory response after thoracic epidural injection of RTX, with increases in heart rate (HR) and left ventricular systolic pressure (LVSP), which stabilized at approximately 2 hours (Supplemental Figure 1). However, it is not what Supplemental Figure 1 showed. At the 120 min point, LVSP is still about 10% higher, and dP/dtmax is still about 20% lower than the baseline. HR returns to baseline at about 75 min, and dP/dtmin returns at about 90 min. Can the author further explain how they determine the stabilization of the hemodynamic parameters?

Thank you for raising this point. We agree that the use of the word "stabilization" is unclear. As heart rate and systolic pressure are two parameters that were readily in the clinical setting (in contrast to inotropy and lusitropy, which require a left ventricular catheter), we decided to primarily determine 'hemodynamic stability' through these two parameters.

We agree that the term 'stabilization' was unclear. To clarify, we have revised these sentences to state that no further changes in heart rate or blood pressure were observed after RTX injection after one hour. The text has been clarified the text and additional data in the supplementary figures provided as below.

<u>Page 5, Lines 117-125:</u>

"To characterize the safety, efficacy and pharmacodynamic profile of RTX, we first administered epidural RTX to 3 healthy control animals. In these experiments we primarily focused on changes in heart rate (HR) and left ventricular systolic pressure (LVSP), as these parameters (or their surrogates) can be readily assessed intra-operatively in humans, without the need for intraventricular catheters. After RTX administration, we observed an initial sympathoexcitatory response characterized by increased HR and LVSP, followed by stabilization of both hemodynamic parameters over the next 30-60 minutes (Supplemental Figure 1)."

Supplemental Figure 1

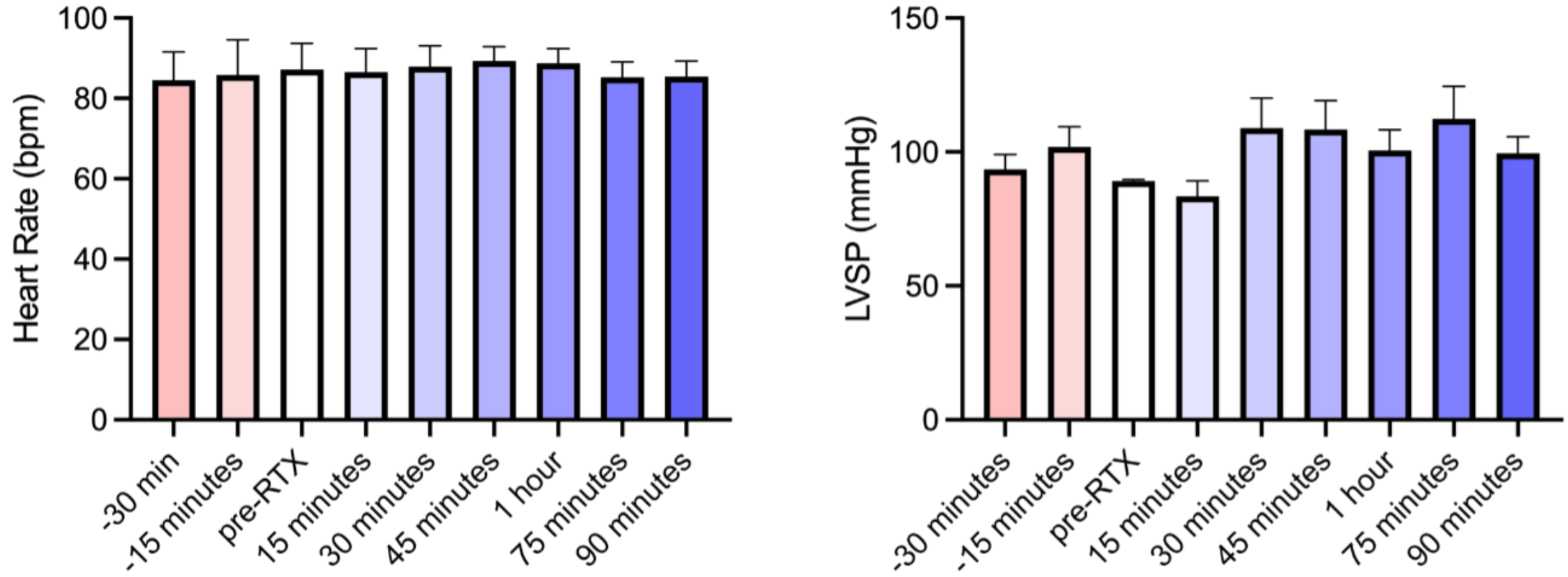

In the above figure, it can be observed that after one hour of RTX, no further significant changes in heart rate or LVSP. The variability in heart rate and blood pressure is consistent with physiological variability that can be observed over time in anesthetized animals and humans, even before RTX administration.

3, Line 116, "subsequent animals" should be defined. Are they normal animals or infarcted animals? It is a long and complicated sentence. Perhaps the authors can break it down and specify the procedures/groups of the n=11 and n=13. The words highlighted by parentheses can be a separate sentence.

We thank the reviewer for this great suggestion. With this statement, we merely aimed to highlight that we initiated the studies with the three healthy animals. The sentence has been rewritten to the following:

Page 5-6, Lines 125-127:

"Accordingly, for the subsequent chronic studies, myocardial infarction was induced 2-3 hours after C7-T1 epidural RTX (cRTX; n=11) or epidural saline (vehicle; n=13) administration."

4, The authors used "sympathetic nociceptive afferent signaling" in the title, but the text does not explicitly define that term. Are you talking about nerves that are both sympathetic and nociceptive? Nociceptive afferents are sensory neurons. Their cell bodies are located in the DRG and express CGRP. Sympathetic neurons are efferent neurons. They can interact, but they are separate systems. We must be cautious about using CGRP as a biomarker of sympathetic nociceptive neuronal ablation. There is also no direct evidence of sympathetic remodeling in either group in this model. Finally, does RTX kill sympathetic nerves?

We thank the reviewer for raising this point. By sympathetic, we mean afferent fibers that travel with sympathetic nerves (as opposed to the vagal nerves and trunk). In the literature, spinal afferents and sympathetic afferents are often used interchangeably, as spinal afferents initiate a sympathetic reflex. However, these afferent fibers are not the same as sympathetic post-ganglionic efferents. To clarify, we have changed "sympathetic" to "spinal" in the title of the study and throughout the manuscript.

We agree with the reviewer that CGRP is a marker for TRPV1 nociceptive afferent neurons. As the reviewer points out, activation of TRPV1 has been repeatedly shown to induce the release of CGRP[18, 94-99]. Therefore, CGRP has been used as a biomarker for these neurons. Despite testing multiple antibodies, we could not find an adequate TRPV1 antibody, including with the use of those published in a prior porcine study, that provided adequate signal over background in the pig tissue tested. To both clarify and acknowledge that CGRP (rather than TRPV1) was used as a biomarker, the following have been stated in the results and limitations:

Page 6, Lines 148-150:

"As most TRPV1-expressing nociceptive neurons co-express and release calcitonin gene-related peptide (CGRP)[45, 46], we used CGRP immunoreactivity (IR) as a marker for these nociceptive afferents."

Page 22, Lines 528-532:

"Lastly, in this study, CGRP was used as a surrogate for TRPV1-expressing nociceptive neurons/fibers, as direct TRPV1 immunolabeling was unreliable in our hands. However, activation of TRPV1 has been reliably shown to evoke the release of neurotransmitters such as CGRP[96-99]. Therefore, CGRP has been widely used as a marker for TRPV1-expressing afferent neurons"

Lastly, the reviewer raises the very interesting point of whether RTX affected sympathetic efferent fibers. As RTX is an ultrapotent analog of the TRPV1 receptor[9-13], and sympathetic cardiomotor efferent neurons do not express TRPV1[100, 101], the epidural administration of RTX is unlikely to have affected these neurons/fibers. To further confirm this, we performed experiments assessing CGRP and TH immunoreactivity in the ventricular myocardium (viable regions). This data showed that while innervation by CGRP immunoreactive fibers is reduced, TH innervation is unchanged in RTX-treated vs vehicle-treated animals. These new panels/data have been added to the new figure 2L-J.

Figure 2

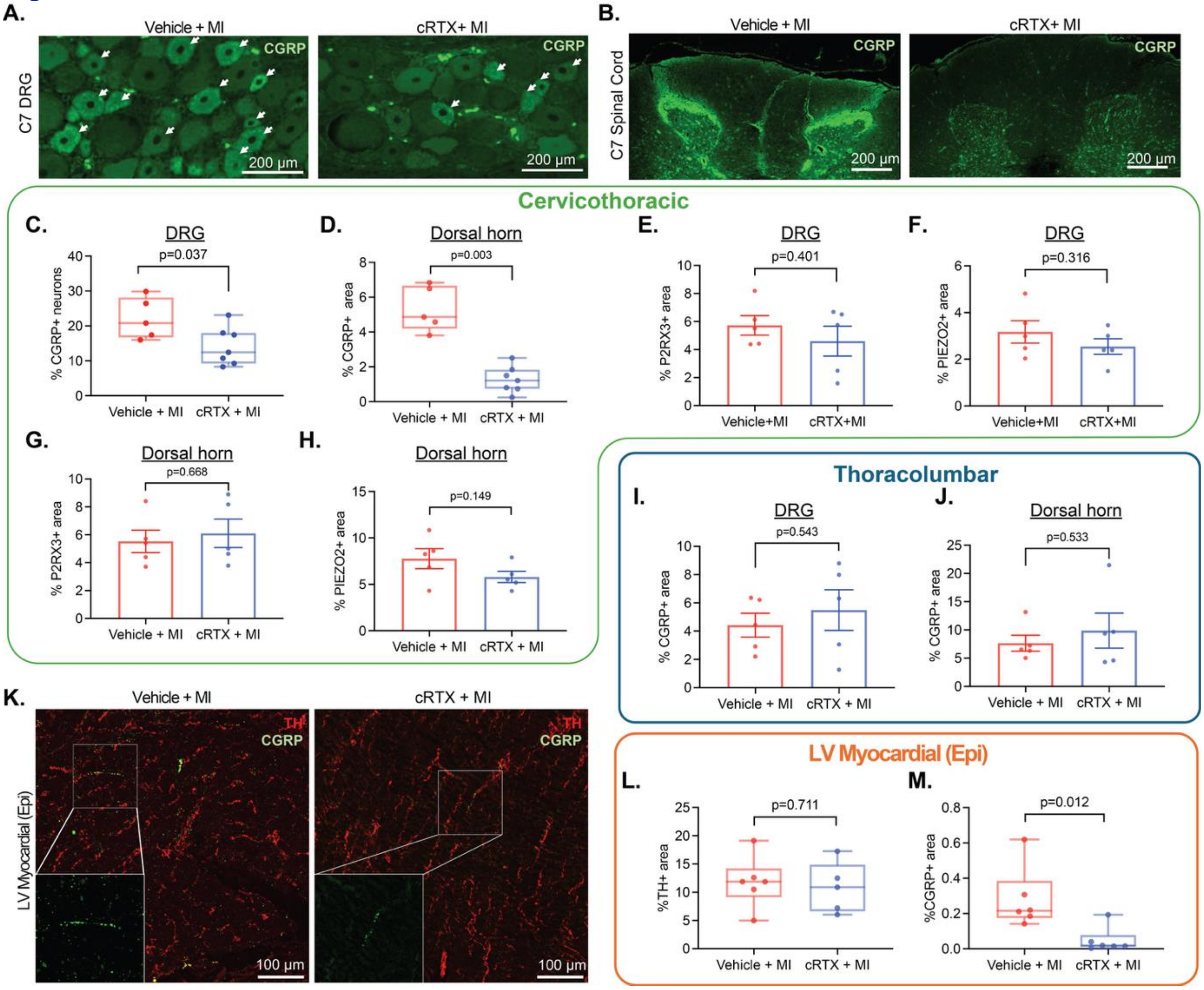

5, in Ref #87, which performed a very similar study, also gave RTX to pigs' thoracic dorsal root ganglion. All animals had a dosage of 0.5 ml (100 µg/mL). In this study, the authors used 0.6-1.2 µg/kg. Given the BW of those pigs the author gave (40.4±0.7 kg), the RTX dosage varied between 24 µg and 48 µg

in different animals. Could the author explain which animals received 24 and which received 48, and if the animals receiving the double dose experienced more antiarrhythmic effects?

We thank the reviewer for this insightful comment. We now clarify in the manuscript which animals received what dose of RTX. All animals in the group that received epidural RTX prior to MI received a dose of 1.2 µg/kg. For the animals that received RTX four-to-six weeks after MI, in 8 of 15 animals we also tried a lower dose of 0.6 µg/kg of RTX, while 7 of 15 received 1.2 µg/kg, to evaluate a limited dose response. Comparing the hemodynamic and electrophysiological profiles of the two doses, there were no significant differences between the two groups. Therefore, we initially reported the data for the manuscript combining all animals. However, we have since added the result of the two different doses to supplementary figure 8 and report hemodynamic and electrophysiological parameters at these two different doses.

Page 25, Lines 609-616:

"All cRTX animals (those receiving RTX prior to MI and studied four-to-six weeks later) received 1.2 µg/kg of RTX in 1 mL. In the chronic infarcted animals that received RTX four to six weeks after MI acutely, 7/15 received 1.2 1.2 µg/kg of RTX while 8/15 a lower dose of 0.6 µg/kg of RTX (also in 1 mL) to evaluate any differences in hemodynamic or electrophysiological responses. As no hemodynamic or electrophysiological differences were observed between the two groups (Supplementary Figure 9), data was pooled for analyses involving the aRTX animals."

Supplemental Figure 9

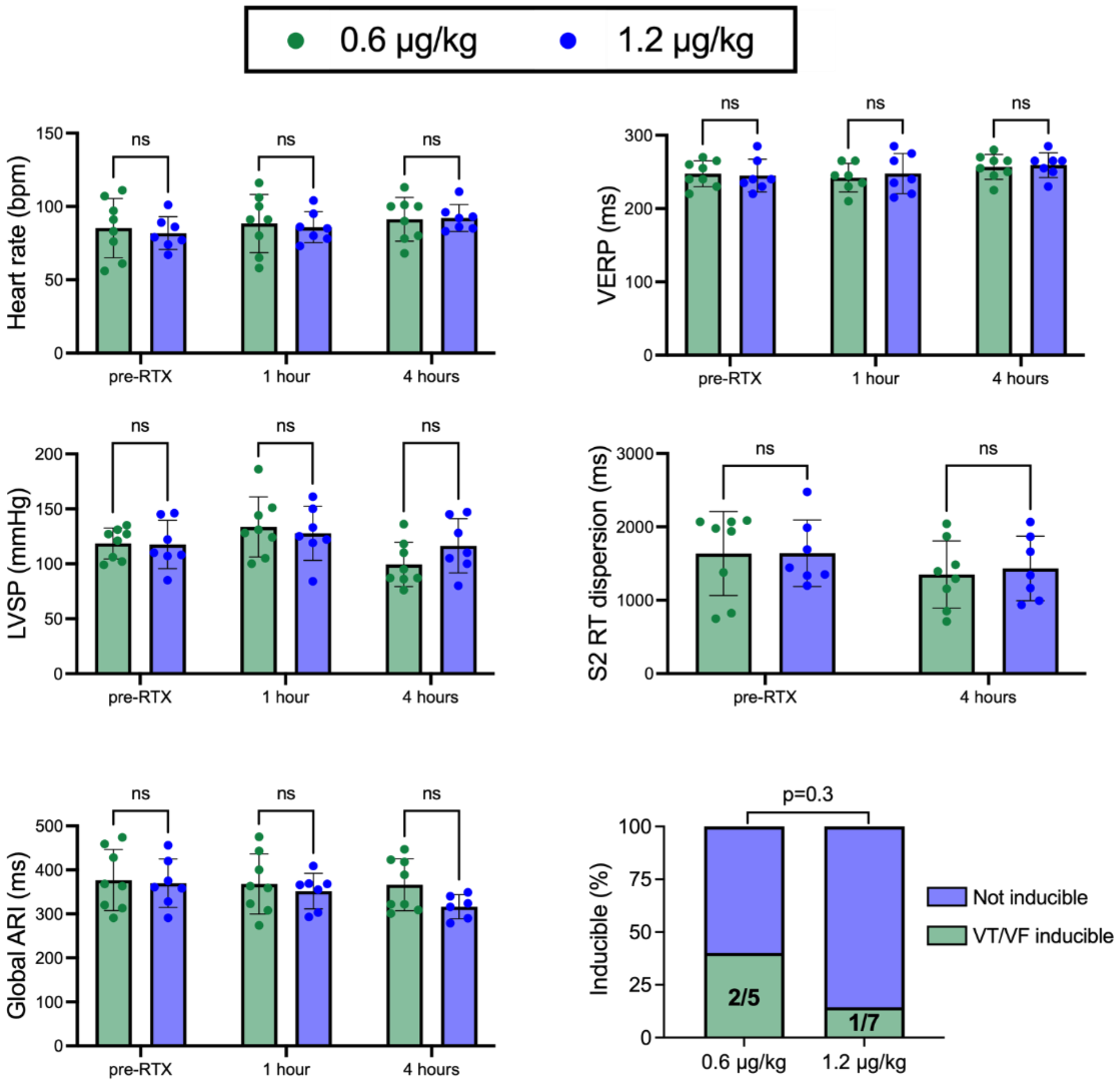

6, In lines 510-511, MI was confirmed by lack of distal LAD flow on coronary angiography coupled with ST-segment changes. How did the authors determine the extent of myocardial infarction? Did the authors define the cardiac enzyme levels? Had the infarction area been measured? Would it be possible for different infarction sizes to influence the autonomic remodeling?

We thank the reviewer for raising this important point and agree that infarct size is indeed an important factor in the assessment of the outcomes of the current study. As such, we have since added

Supplemental figure 7, in which we have defined the infarct size by gross pathological examination as well as by voltage mapping and found that the values were similar between cRTX vs. vehicle-treated infarcted animals.

In addition, we evaluated our intracardiac echocardiography (ICE) images for LV diastolic area, LV systolic area, and %change in LV areas as function (as measure of ejection fraction). These results showed no differences in RTX-treated (n=10) vs. vehicle-treated (n=10) animals. Finally, as noted in Figure 3, LVSP and inotropy, as measured by dP/dt_{max}, were not significantly different at baseline in RTX-treated vs. vehicle-treated animals. dP/dt_{max} has been previously shown to be an important parameter when assessing cardiac function after myocardial infarction[102]. Hence, it appears that differences in infarct size or cardiac function were unlikely to have resulted in the findings of this study. The following sentences, as well as supplemental figure 7, have since been added to the manuscript.

<u>Page 12, Lines 283-297:</u>

> *"Scar size and detailed electrophysiological measurements were evaluated in RTX (n=11) versus vehicle treated infarcted animals (n=13) to determine if epidural RTX prior to MI had altered these after chronic MI. Four-to-six weeks after MI, the size of ventricular scars were not different between those that had received RTX versus vehicle prior to MI (Supplemental Figure 7), as assessed by epicardial LV and RV voltage mapping and quantification of low voltage areas, defined as border zone and scar regions (percent low voltage area: 23.7 ± 2.3% in cRTX (n = 11) vs 22.3 ± 1.9% in vehicle (n=13) animals, p=0.634) and gross pathological examination and image quantification of dense scar regions of explanted hearts (scar size: 10.0 ± 1.3 % vs 10.7 ± 1.3% of anterior ventricular surface in cRTX (n = 11) vs vehicle (n=13) animals, respectively, p=0.690). In addition, analysis of echocardiographic data showed that LV end systolic and end-diastolic areas were not significantly different between the two groups, with a similar change in these dimensions (quantified as LV ejection fraction or % change in systole) of 40.0 ± 2.8% vs 36.0 ± 3.8% in cRTX (n = 5) vs vehicle-treated (n=5) animals, respectively, p=0.540)."*

Supplemental Figure 7

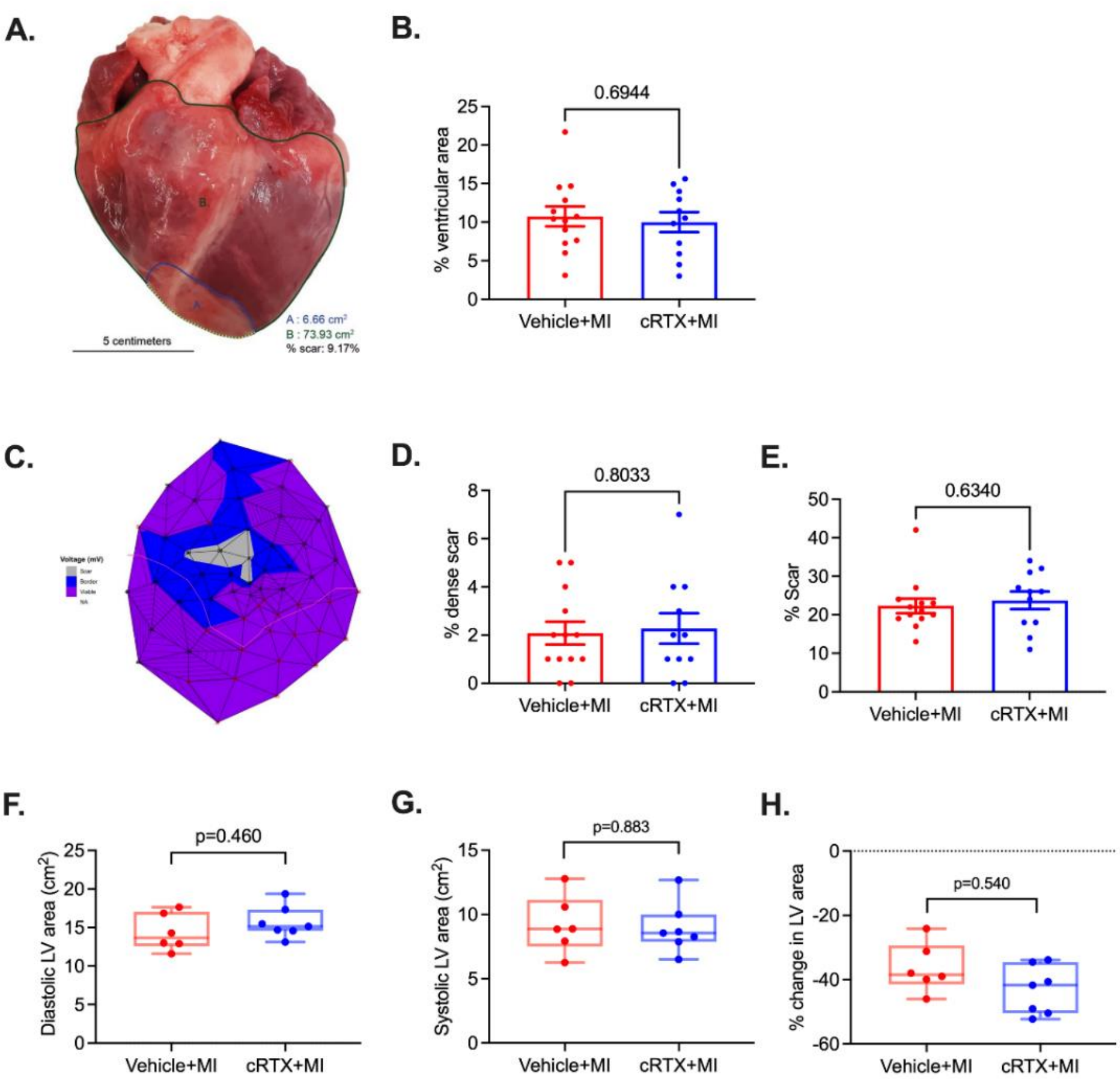

7, Supplemental Figure 3: In the upper panel, it would be better to label 5/18 and 8/19 in the red boxes than in the blue boxes.

We thank the reviewer for this suggestion. We have since moved the labels to the red boxes.

Supplemental Figure 3

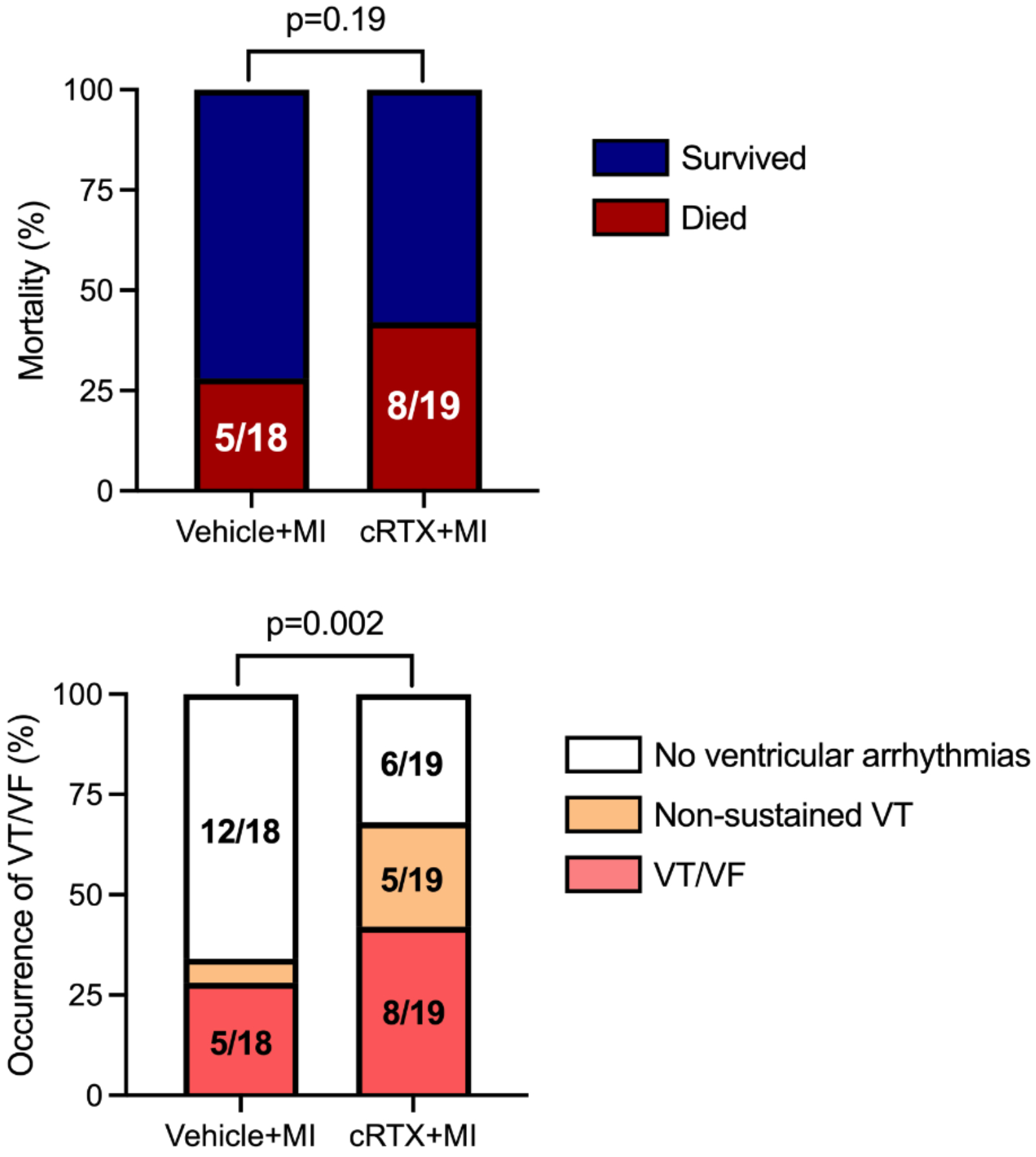

8, Discussion. The discussion is very long. It could be more focused on the mechanisms. The authors have performed a comprehensive study and documented multiple physiological, chemical, and histological changes associated with the RTX ablation. However, these observations do not necessarily indicate that they are responsible for the antiarrhythmic effects. Reference 87 showed that the same intervention is antiarrhythmic in ischemia/reperfusion-induced arrhythmia. While RTX induces changes in glial activation, inflammation, vagal dysfunction etc., are those effects needed to achieve arrhythmia control? I think the readers may appreciate some discussions on the causal relationships related to those observations.

We thank the reviewer for this comment and agree that the discussion needs to be more focused on the mechanistic aspects of the study. We have, therefore, largely rewritten the discussion to better highlight the mechanistic relationship between cardiac spinal afferent ablation and the autonomic, inflammatory, and electrophysiological changes observed in this study while still trying to address other reviewers' concerns.

REFERENCES

1. Hainsworth, R. Reflexes from the heart. *Physiol Rev* **71**, 617-658 (1991).
2. Armour, J.A. & Ardell, J.L. *Basic and clinical neurocardiology* (Oxford University Press, New York, 2004).
3. Recordati, G., Schwartz, P.J., Pagani, M., Malliani, A. & Brown, A.M. Activation of cardiac vagal receptors during myocardial ischemia. *Experientia* **27**, 1423-1424 (1971).
4. Robertson, D., Hollister, A.S., Forman, M.B. & Robertson, R.M. Reflexes unique to myocardial ischemia and infarction. *J Am Coll Cardiol* **5**, 99B-104B (1985).
5. Ustinova, E.E. & Schultz, H.D. Activation of cardiac vagal afferents in ischemia and reperfusion. Prostaglandins versus oxygen-derived free radicals. *Circ Res* **74**, 904-911 (1994).
6. Porszasz, J., Gyorgy, L. & Porszasz-Gibiszer, K. Cardiovascular and respiratory effects of capsaicin. *Acta Physiol Acad Sci Hung* **8**, 61-76 (1955).
7. Bencsik, P.*, et al.* Myocardial ischaemia reperfusion injury and cardioprotection in the presence of sensory neuropathy: Therapeutic options. *Br J Pharmacol* **177**, 5336-5356 (2020).
8. Szabados, T.*, et al.* Capsaicin-Sensitive Sensory Nerves and the TRPV1 Ion Channel in Cardiac Physiology and Pathologies. *Int J Mol Sci* **21**, 4472 (2020).
9. Szallasi, A. & Blumberg, P.M. Resiniferatoxin, a phorbol-related diterpene, acts as an ultrapotent analog of capsaicin, the irritant constituent in red pepper. *Neuroscience* **30**, 515-520 (1989).
10. Bates, B.D.*, et al.* Prolonged analgesic response of cornea to topical resiniferatoxin, a potent TRPV1 agonist. *Pain* **149**, 522-528 (2010).
11. Brown, D.C., Agnello, K. & Iadarola, M.J. Intrathecal resiniferatoxin in a dog model: efficacy in bone cancer pain. *Pain* **156**, 1018-1024 (2015).
12. Brown, D.C.*, et al.* Physiologic and antinociceptive effects of intrathecal resiniferatoxin in a canine bone cancer model. *Anesthesiology* **103**, 1052-1059 (2005).
13. Brown, J.D.*, et al.* CT-guided injection of a TRPV1 agonist around dorsal root ganglia decreases pain transmission in swine. *Sci Transl Med* **7**, 305ra145 (2015).
14. Karai, L.*, et al.* Deletion of vanilloid receptor 1-expressing primary afferent neurons for pain control. *J Clin Invest* **113**, 1344-1352 (2004).
15. Sapio, M.R.*, et al.* Pain control through selective chemo-axotomy of centrally projecting TRPV1+ sensory neurons. *J Clin Invest* **128**, 1657-1670 (2018).
16. Karai, L.J., Russell, J.T., Iadarola, M.J. & Olah, Z. Vanilloid receptor 1 regulates multiple calcium compartments and contributes to Ca2+-induced Ca2+ release in sensory neurons. *J Biol Chem* **279**, 16377-16387 (2004).
17. Olah, Z.*, et al.* Ligand-induced dynamic membrane changes and cell deletion conferred by vanilloid receptor 1. *J Biol Chem* **276**, 11021-11030 (2001).
18. Szallasi, A., Cortright, D.N., Blum, C.A. & Eid, S.R. The vanilloid receptor TRPV1: 10 years from channel cloning to antagonist proof-of-concept. *Nat Rev Drug Discov* **6**, 357-372 (2007).
19. Jones, S.*, et al.* Bradykinin excites rat sympathetic neurons by inhibition of M current through a mechanism involving B2 receptors and G alpha q/11. *Neuron* **14**, 399-405 (1995).
20. Mathivanan, S., Devesa, I., Changeux, J.P. & Ferrer-Montiel, A. Bradykinin Induces TRPV1 Exocytotic Recruitment in Peptidergic Nociceptors. *Front Pharmacol* **7**, 178 (2016).
21. Cesare, P., Dekker, L.V., Sardini, A., Parker, P.J. & McNaughton, P.A. Specific involvement of PKC-epsilon in sensitization of the neuronal response to painful heat. *Neuron* **23**, 617-624 (1999).
22. Chuang, H.H.*, et al.* Bradykinin and nerve growth factor release the capsaicin receptor from PtdIns(4,5)P2-mediated inhibition. *Nature* **411**, 957-962 (2001).
23. Yoshie, K.*, et al.* Cardiac TRPV1 afferent signaling promotes arrhythmogenic ventricular remodeling after myocardial infarction. *JCI Insight* **5** (2020).
24. Mannes, A.J.*, et al.* Treatment of Intractable Cancer Pain with Resiniferatoxin - An Interim Study. *NEJM Evid* **4**, EVIDoa2400423 (2025).
25. Szabo, T., Olah, Z., Iadarola, M.J. & Blumberg, P.M. Epidural resiniferatoxin induced prolonged regional analgesia to pain. *Brain Res* **840**, 92-98 (1999).
26. Unger, M.D.*, et al.* Unilateral Epidural Targeting of Resiniferatoxin Induces Bilateral Neurolysis of Spinal Nociceptive Afferents. *Pain Med* **20**, 897-906 (2019).
27. Foreman, R.D. Mechanisms of cardiac pain. *Annu Rev Physiol* **61**, 143-167 (1999).
28. Ide, R., Saiki, C., Makino, M. & Matsumoto, S. TRPV1 receptor expression in cardiac vagal afferent neurons of infant rats. *Neurosci Lett* **507**, 67-71 (2012).

29. Mohammed, M., Madden, C.J., Andresen, M.C. & Morrison, S.F. Activation of TRPV1 in nucleus tractus solitarius reduces brown adipose tissue thermogenesis, arterial pressure, and heart rate. *Am J Physiol Regul Integr Comp Physiol* **315**, R134-R143 (2018).
30. Zhai, K., Liskova, A., Kubatka, P. & Busselberg, D. Calcium Entry through TRPV1: A Potential Target for the Regulation of Proliferation and Apoptosis in Cancerous and Healthy Cells. *Int J Mol Sci* **21** (2020).
31. Bautista, D. & Julius, D. Fire in the hole: pore dilation of the capsaicin receptor TRPV1. *Nat Neurosci* **11**, 528-529 (2008).
32. Pingle, S.C., Matta, J.A. & Ahern, G.P. Capsaicin receptor: TRPV1 a promiscuous TRP channel. *Handb Exp Pharmacol*, 155-171 (2007).
33. Caterina, M.J.*, et al.* The capsaicin receptor: a heat-activated ion channel in the pain pathway. *Nature* **389**, 816-824 (1997).
34. Jordt, S.E., Tominaga, M. & Julius, D. Acid potentiation of the capsaicin receptor determined by a key extracellular site. *Proc Natl Acad Sci U S A* **97**, 8134-8139 (2000).
35. Bohlen, C.J.*, et al.* A bivalent tarantula toxin activates the capsaicin receptor, TRPV1, by targeting the outer pore domain. *Cell* **141**, 834-845 (2010).
36. Cuypers, E., Yanagihara, A., Karlsson, E. & Tytgat, J. Jellyfish and other cnidarian envenomations cause pain by affecting TRPV1 channels. *FEBS Lett* **580**, 5728-5732 (2006).
37. Siemens, J.*, et al.* Spider toxins activate the capsaicin receptor to produce inflammatory pain. *Nature* **444**, 208-212 (2006).
38. Stevens, R.M.*, et al.* Randomized, Double-Blind, Placebo-Controlled Trial of Intraarticular Trans-Capsaicin for Pain Associated With Osteoarthritis of the Knee. *Arthritis Rheumatol* **71**, 1524-1533 (2019).
39. Ostenfeld, T., Young, N., Eibl, C. & Ivanavicius, S. Evaluation of the safety and analgesic effect of the TRPV1 agonist RTX-GRT7039 – an open-label, single dose study in patients with chronic osteoarthritic knee joint pain (Study P02). *Osteoarthritis Cartilage* **32** (2024).
40. Ostenfeld, T., Young, N., Eibl, C. & Ivanavicius, S. Preliminary evaluation of the safety and analgesic effect of the Trpv1 agonist Rtx-Grt7039-an open-label, first-in-human study in patients with chronic pain due to knee osteoarthritis (study P01). *Osteoarthritis Cartilage* **32** (2024).
41. Leiman, D.*, et al.* Preliminary results from a phase 1b double-blind study to assess the safety, tolerability and efficacy of intra-articular administration of resiniferatoxin or placebo for the treatment of moderate to severe pain due to osteoarthritis of the knee. *Osteoarthritis Cartilage* **28** (2020).
42. Liu, L. & Simon, S.A. A rapid capsaicin-activated current in rat trigeminal ganglion neurons. *Proc Natl Acad Sci U S A* **91**, 738-741 (1994).
43. Grant, E.R., Dubin, A.E., Zhang, S.P., Zivin, R.A. & Zhong, Z. Simultaneous intracellular calcium and sodium flux imaging in human vanilloid receptor 1 (VR1)-transfected human embryonic kidney cells: a method to resolve ionic dependence of VR1-mediated cell death. *J Pharmacol Exp Ther* **300**, 9-17 (2002).
44. Caudle, R.M.*, et al.* Resiniferatoxin-induced loss of plasma membrane in vanilloid receptor expressing cells. *Neurotoxicology* **24**, 895-908 (2003).
45. Maggi, C.A. Tachykinins and calcitonin gene-related peptide (CGRP) as co-transmitters released from peripheral endings of sensory nerves. *Prog Neurobiol* **45**, 1-98 (1995).
46. Gibson, S.J.*, et al.* Calcitonin gene-related peptide immunoreactivity in the spinal cord of man and of eight other species. *J Neurosci* **4**, 3101-3111 (1984).
47. Xie, K., Cheng, X., Zhu, T. & Zhang, D. Single-cell transcriptomic profiling of dorsal root ganglion: an overview. *Front Neuroanat* **17**, 1162049 (2023).
48. Yu, H.*, et al.* Leveraging deep single-soma RNA sequencing to explore the neural basis of human somatosensation. *Nat Neurosci* **27**, 2326-2340 (2024).
49. Kummer, W., Fischer, A., Kurkowski, R. & Heym, C. The sensory and sympathetic innervation of guinea-pig lung and trachea as studied by retrograde neuronal tracing and double-labelling immunohistochemistry. *Neuroscience* **49**, 715-737 (1992).
50. Dinh, Q.T.*, et al.* Substance P expression in TRPV1 and trkA-positive dorsal root ganglion neurons innervating the mouse lung. *Respir Physiol Neurobiol* **144**, 15-24 (2004).
51. Nassenstein, C.*, et al.* Phenotypic distinctions between neural crest and placodal derived vagal C-fibres in mouse lungs. *J Physiol* **588**, 4769-4783 (2010).
52. McGovern, A.E.*, et al.* Anterograde neuronal circuit tracing using a genetically modified herpes simplex virus expressing EGFP. *J Neurosci Methods* **209**, 158-167 (2012).

53. Canning, B.J., Mori, N. & Mazzone, S.B. Vagal afferent nerves regulating the cough reflex. *Respir Physiol Neurobiol* **152**, 223-242 (2006).
54. Carr, M.J. & Undem, B.J. Bronchopulmonary afferent nerves. *Respirology* **8**, 291-301 (2003).
55. Trankner, D., Hahne, N., Sugino, K., Hoon, M.A. & Zuker, C. Population of sensory neurons essential for asthmatic hyperreactivity of inflamed airways. *Proc Natl Acad Sci U S A* **111**, 11515-11520 (2014).
56. Widdicombe, J. Airway receptors. *Respir Physiol* **125**, 3-15 (2001).
57. Chang, R.B., Strochlic, D.E., Williams, E.K., Umans, B.D. & Liberles, S.D. Vagal Sensory Neuron Subtypes that Differentially Control Breathing. *Cell* **161**, 622-633 (2015).
58. Dalsgaard, C.J. & Lundberg, J.M. Evidence for a spinal afferent innervation of the guinea pig lower respiratory tract as studied by the horseradish peroxidase technique. *Neurosci Lett* **45**, 117-122 (1984).
59. Kostreva, D.R., Hopp, F.A. & Kampine, J.P. Depressor responses to stimulation of sympathetic afferents in monkeys and dogs. *Am J Physiol* **240**, R23-28 (1981).
60. Kim, S.H., *et al.* Mapping of the Sensory Innervation of the Mouse Lung by Specific Vagal and Dorsal Root Ganglion Neuronal Subsets. *eNeuro* **9** (2022).
61. Weaver, L.C. Cardiopulmonary sympathetic afferent influences on renal nerve activity. *Am J Physiol* **233**, H592-599 (1977).
62. Wang, Y., Soukhova, G., Proctor, M., Walker, J. & Yu, J. Bradykinin causes hypotension by activating pulmonary sympathetic afferents in the rabbit. *J Appl Physiol (1985)* **95**, 233-240 (2003).
63. Soukhova-O'Hare, G.K., Zhang, J.W., Gozal, D. & Yu, J. Bradykinin B2 receptors mediate pulmonary sympathetic afferents induced reflexes in rabbits. *Life Sci* **78**, 1990-1997 (2006).
64. Hooper, J.S., *et al.* Characterization of cardiovascular reflexes evoked by airway stimulation with allylisothiocyanate, capsaicin, and ATP in Sprague-Dawley rats. *J Appl Physiol (1985)* **120**, 580-591 (2016).
65. Shanks, J., *et al.* Sympatho-excitatory response to pulmonary chemosensitive spinal afferent activation in anesthetized, vagotomized rats. *Physiol Rep* **6**, e13742 (2018).
66. Oh, E.J., Mazzone, S.B., Canning, B.J. & Weinreich, D. Reflex regulation of airway sympathetic nerves in guinea-pigs. *J Physiol* **573**, 549-564 (2006).
67. Berthoud, H.R. Anatomy and function of sensory hepatic nerves. *Anat Rec A Discov Mol Cell Evol Biol* **280**, 827-835 (2004).
68. Goehler, L.E. & Sternini, C. Calcitonin gene-related peptide innervation of the rat hepatobiliary system. *Peptides* **17**, 209-217 (1996).
69. Tiegs, G., Bang, R. & Neuhuber, W.L. Requirement of peptidergic sensory innervation for disease activity in murine models of immune hepatitis and protection by beta-adrenergic stimulation. *J Neuroimmunol* **96**, 131-143 (1999).
70. Magni, F. & Carobi, C. The afferent and preganglionic parasympathetic innervation of the rat liver, demonstrated by the retrograde transport of horseradish peroxidase. *J Auton Nerv Syst* **8**, 237-260 (1983).
71. Carobi, C. & Magni, F. The afferent innervation of the liver: a horseradish peroxidase study in the rat. *Neurosci Lett* **23**, 269-274 (1981).
72. Carobi, C. & Magni, F. Capsaicin-sensitive afferent vagal neurons innervating the rat liver. *Neurosci Lett* **62**, 261-265 (1985).
73. Tiniakos, D.G., Mathew, J., Kittas, C. & Burt, A.D. Ontogeny of human intrahepatic innervation. *Virchows Arch* **452**, 435-442 (2008).
74. Chen, C.C., *et al.* Human liver afferent and efferent nerves revealed by 3-D/Airyscan super-resolution imaging. *Am J Physiol Endocrinol Metab* **326**, E107-E123 (2024).
75. Shanks, J., de Morais, S.D.B., Gao, L., Zucker, I.H. & Wang, H.J. TRPV1 (Transient Receptor Potential Vanilloid 1) Cardiac Spinal Afferents Contribute to Hypertension in Spontaneous Hypertensive Rat. *Hypertension* **74**, 910-920 (2019).
76. Wu, M., *et al.* Innervation of nociceptor neurons in the spleen promotes germinal center responses and humoral immunity. *Cell* **187**, 2935-2951 e2919 (2024).
77. Pavlov, V.A. & Tracey, K.J. Neural regulation of immunity: molecular mechanisms and clinical translation. *Nat Neurosci* **20**, 156-166 (2017).
78. Pavlov, V.A. & Tracey, K.J. The vagus nerve and the inflammatory reflex--linking immunity and metabolism. *Nat Rev Endocrinol* **8**, 743-754 (2012).
79. Marsal, S., *et al.* Non-invasive vagus nerve stimulation for rheumatoid arthritis: a proof-of-concept study. *Lancet Rheumatol* **3**, e262-e269 (2021).

80. Sahn, B., Pascuma, K., Kohn, N., Tracey, K.J. & Markowitz, J.F. Transcutaneous auricular vagus nerve stimulation attenuates inflammatory bowel disease in children: a proof-of-concept clinical trial. *Bioelectron Med* **9**, 23 (2023).
81. Pikov, V. Vagus Nerve Stimulation and Sacral Nerve Stimulation for Inflammatory Bowel Disease: A Systematic Review. *J Transl Gastroenterol* **1**, 94-100 (2023).
82. Baker, M.C., Nagy, D., Tamang, S., Horvath-Puho, E. & Sorensen, H.T. Vagotomy and the incidence of rheumatoid arthritis and osteoarthritis: a Danish register-based study. *Arthritis Res Ther* **27**, 106 (2025).
83. Perrotta, S.*, et al.* A heart-brain-spleen axis controls cardiac remodeling to hypertensive stress. *Immunity* **58**, 648-665 e647 (2025).
84. Rosas-Ballina, M.*, et al.* Acetylcholine-synthesizing T cells relay neural signals in a vagus nerve circuit. *Science* **334**, 98-101 (2011).
85. Borovikova, L.V.*, et al.* Vagus nerve stimulation attenuates the systemic inflammatory response to endotoxin. *Nature* **405**, 458-462 (2000).
86. Huston, J.M. & Tracey, K.J. The pulse of inflammation: heart rate variability, the cholinergic anti-inflammatory pathway and implications for therapy. *J Intern Med* **269**, 45-53 (2011).
87. Campese, V.M. & Kogosov, E. Renal afferent denervation prevents hypertension in rats with chronic renal failure. *Hypertension* **25**, 878-882 (1995).
88. Donovan, M.K., Wyss, J.M. & Winternitz, S.R. Localization of renal sensory neurons using the fluorescent dye technique. *Brain Res* **259**, 119-122 (1983).
89. Weiss, M.L. & Chowdhury, S.I. The renal afferent pathways in the rat: a pseudorabies virus study. *Brain Res* **812**, 227-241 (1998).
90. Yamaguchi, T.*, et al.* Thoracic Dorsal Root Ganglion Application of Resiniferatoxin Reduces Myocardial Ischemia-Induced Ventricular Arrhythmias. *Biomedicines* **11** (2023).
91. van Weperen, V.Y.H., Ripplinger, C.M. & Vaseghi, M. Autonomic control of ventricular function in health and disease: current state of the art. *Clin Auton Res* **33**, 1-27 (2023).
92. Dusi, V., De Ferrari, G.M. & Schwartz, P.J. There are 100 ways by which the sympathetic nervous system can trigger life-threatening arrhythmias. *Eur Heart J* **41**, 2180-2182 (2020).
93. Gardner, R.T., Ripplinger, C.M., Myles, R.C. & Habecker, B.A. Molecular Mechanisms of Sympathetic Remodeling and Arrhythmias. *Circ Arrhythm Electrophysiol* **9**, e001359 (2016).
94. Kee, Z., Kodji, X. & Brain, S.D. The Role of Calcitonin Gene Related Peptide (CGRP) in Neurogenic Vasodilation and Its Cardioprotective Effects. *Front Physiol* **9**, 1249 (2018).
95. Szallasi, A. & Blumberg, P.M. Vanilloid (Capsaicin) receptors and mechanisms. *Pharmacol Rev* **51**, 159-212 (1999).
96. Kilo, S., Harding-Rose, C., Hargreaves, K.M. & Flores, C.M. Peripheral CGRP release as a marker for neurogenic inflammation: a model system for the study of neuropeptide secretion in rat paw skin. *Pain* **73**, 201-207 (1997).
97. Meng, J.*, et al.* Activation of TRPV1 mediates calcitonin gene-related peptide release, which excites trigeminal sensory neurons and is attenuated by a retargeted botulinum toxin with anti-nociceptive potential. *J Neurosci* **29**, 4981-4992 (2009).
98. Meng, J., Wang, J., Lawrence, G. & Dolly, J.O. Synaptobrevin I mediates exocytosis of CGRP from sensory neurons and inhibition by botulinum toxins reflects their anti-nociceptive potential. *J Cell Sci* **120**, 2864-2874 (2007).
99. Strecker, T., Messlinger, K., Weyand, M. & Reeh, P.W. Role of different proton-sensitive channels in releasing calcitonin gene-related peptide from isolated hearts of mutant mice. *Cardiovasc Res* **65**, 405-410 (2005).
100. Watase, T.*, et al.* Involvement of transient receptor potential vanilloid 1 channel expression in orofacial cutaneous hypersensitivity following tooth pulp inflammation. *J Oral Sci* **60**, 8-13 (2018).
101. Greffrath, W., Binzen, U., Schwarz, S.T., Saaler-Reinhardt, S. & Treede, R.D. Co-expression of heat sensitive vanilloid receptor subtypes in rat dorsal root ganglion neurons. *Neuroreport* **14**, 2251-2255 (2003).
102. Garamvolgyi, R.*, et al.* dP/dt(max): An underestimated prognostic factor in large animal infarction model. *Animal Model Exp Med* **8**, 171-178 (2025).

RESPONSE TO EDITORS AND REVIEWERS

Response to Reviewer 3
no more comments.

We are thankful for the reviewer's thorough assessment of our manuscript.

Response to Reviewer 4:

The authors have addressed the reviewers' concerns. However, as the reviewers pointed out, it should be emphasized that the findings are similar to Yamaguchi et al, albeit on different timescales.

We are thankful for the reviewer's suggestion to further highlight similarities between the current study and the study by Yamaguchi *et al.* As noted in the manuscript, the study by Yamaguchi *et al.* showed the acute anti-arrhythmic effects of 1) direct application of RTX on thoracic dorsal root ganglia in 2) a porcine model of acute ischemia.

In the current manuscript, we have highlighted similarities and differences in both the introduction and discussion (Lines 95 – 99, 480 – 483).

To further emphasize these prior findings, we have also since added the following sentence to the discussion:

Line 409-502:

"These findings are in line with prior work demonstrating the acute anti-arrhythmic effects of direct RTX application on thoracic DRGs in a porcine model of acute ischemia, and extend these observations from the acute ischemic setting to the chronic development and maintenance of anti-arrhythmic benefit and mitigated autonomic remodeling in the setting of chronic MI."

Response to Reviewer 5:

The revised manuscript is improved and addresses many of the prior concerns. The added histology, infarct-size/remodeling controls, and the acute post-MI RTX experiments strengthen the study and increase its potential translational relevance. Overall, the revised data support a role for cervicothoracic spinal nociceptive afferent signaling in post-MI autonomic/electrophysiological remodeling and arrhythmia susceptibility in this porcine model, although several interpretive and methodological issues remain.

We are grateful to the reviewer for his/her careful evaluation of our manuscript.

a) The direct link between cardiac spinal nociceptive afferents and post-MI cardiac performance is strengthened, and the specificity of RTX manipulation is supported by the additional controls. But the evidence remains incomplete without a direct comparison of $CGRP^+$ fibers between RTX and vehicle groups in the kidney, spleen, and lung.

As the reviewer correctly states, no direct comparison of CGRP+ fibers in the kidney, spleen, and lung has been made between RTX and vehicle groups, which could potentially limit the assessment of off-target effects of high thoracic epidural administration of RTX. Therefore, we agree with the reviewer that the current data does not prove that cervicothoracic administration of epidural RTX does not affect other organs, specifically those in the C7 to T4 dorsal root ganglia, which includes the lungs.

With regards to the kidneys and spleen, spleen receives innervation from the lower thoracic (T8-T13) dorsal root ganglia while the kidney receives innervation from the thoracolumbar dorsal root ganglia (T10-L2/L3), which are 4-5 vertebral spaces away from our site of injection. Prior evidence suggests that it is unlikely that the epidural application of RTX at the C7/T1 level would affect and have had off-target effects on the kidneys and spleen.

As demonstrated by Figure 2 panel I-J in our model, and in line with a prior study by Shanks et al. (2020)[1] epidural administration of RTX in rats, due to the fatty nature of the epidural space, only allows for minimal diffusion of the drug (at most, spread two segments cranial or caudal). Similarly, Szabo *et al.* (1999)[2] demonstrated that epidural administration of RTX at the L4 level in rats, affected the hind paws, but not the front paws, further highlighting the local effects of epidural RTX. In addition, Kim *et al.* (2012)[3] performed a study exploring the extent of diffusion of various compounds after epidural injection in rats. They showed that even compounds such as lidocaine or methylene blue only diffuse up to two segments. Therefore, it would be less likely that a C7-T1 administration would have diffused (especially in non-upright animals) to lower thoracic, or even lumbar, levels, and indeed our evaluation of T12-L1 DRGs does not show affects at this level.

We do acknowledge and agree with the reviewer that pulmonary TRPV1 innervation is likely to be affected by epidural RTX as the spinal afferent innervation originates from the T1-T6 segments[4-6]. However, the presence of spinal TRPV1 sensory afferents in the lung is somewhat limited, as demonstrated by Kim et al. (2022)[7], who showed that whereas vagal afferent fibers innervate almost all conducting airways and project into the alveolar regions, DRG (spinal) afferents only innervate large airways. Moreover, of these DRG/spinal afferents, only 2% express TRPV1[7]. In addition, very little is known about the physiological role of these TRPV1 spinal afferents in the lung, and studies exploring the physiological effects of their activation have demonstrated opposing effects on cardiovascular function[8-13]. Moreover, we did not observe any respiratory changes or discomfort in our animals during the 4-6 weeks post-operative period in the cRTX vs. vehicle-treated chronic MI animals.

Unfortunately, due to the changes in laboratory spaces and difficulties with infrastructure/facilities that occurred in the interval and the interval time passed since the initial submission of manuscript (over 1.5 years), tissues from lungs, spleen, or kidneys from RTX-treated animals are no longer available to us. Hence, we cannot fully exclude any off-target effects of the C7-T1 epidural administration of RTX. Given this and as we agree with the reviewer, we have revised the manuscript, including the title, to remove the word cardiac (as we cannot prove that the benefits seen were purely due to cardiac spinal afferents, and not lung, for example), revised the conclusions accordingly, and further highlighted the reviewer's point in the limitations.

<u>Title</u>

Spinal nociceptive denervation impedes chronic autonomic remodeling after myocardial infarction

<u>Line 375 – 377:</u>

"*The present study demonstrates that cervicothoracic spinal nociceptive afferent signaling is a critical factor in initiating and sustaining the chronic pathological autonomic remodeling after MI (Abstract Illustration).*"

<u>Line 565 – 567:</u>

"Cervicothoracic spinal/sympathetic nociceptive afferent signaling plays a significant role in the occurrence of the multitude of reported, pathological, autonomic changes after chronic MI."

Lastly, the following sentence was added to the manuscript

<u>Line 551 – 555:</u>

"Nevertheless, as we did not perform immunohistochemistry for CGRP on pulmonary, splenic, renal or hepatic tissue, potential off-target effects or benefit, which may have contributed to the results observed, cannot be completely ruled out."

b) There are still instances of overstatement throughout the manuscript. The authors use the term "prevent" in multiple places, including the title ("Cardiac spinal 1 nociceptive afferent denervation with epidural resiniferatoxin prevents chronic structural and functional autonomic remodeling after myocardial infarction") and a section heading ("RTX therapy prevents post-MI oxidative stress, inflammation, and glial activation"). As comparisons in the study are limited to vehicle-treated and RTX-treated MI groups, without inclusion of non-MI controls, it is difficult to assess the extent of improvement with RTX or whether the rescue is complete. The use of "prevent" is therefore potentially misleading, as it implies complete protection or recovery.

We thank the reviewer for this comment and agree that the term "preventing" can be misleading as no direct comparison with healthy animals were made. We have, therefore, made changes throughout the manuscript as below and removed the word prevent.

We have changed the title to:

Spinal nociceptive denervation impedes chronic autonomic remodeling after myocardial infarction

Similarly, we have changed the wording in the following sentences:

<u>Line 253</u>

"RTX therapy impedes post-MI oxidative stress, inflammation, and glial activation"

<u>Line 322-326</u>

"Having shown that administration of epidural RTX and ablation of cervicothoracic nociceptive neurons prior to MI (cRTX) impeded autonomic/electrophysiological remodeling and reduced VT/VF inducibility at 4-6 weeks post-MI, we next asked whether RTX could still be of therapeutic benefit after pathological remodeling has been established."

<u>Line 370-373</u>

"Local interruption of spinal TRPV1 afferent signaling after MI impeded the development of vagal dysfunction, suppressed neuroinflammation and glial activation, attenuated systemic inflammation and oxidative stress, and stabilized cardiac electrophysiological parameters, reducing VT/VF inducibility."

c) The authors did not characterize changes in the local immune milieu or myocardial inflammatory remodeling in the heart, which could contribute to the improved cardiac function in the RTX-treated group. This limitation should be explicitly acknowledged

We agree with the reviewer that changes in myocardial inflammatory remodeling might have participated in the results observed in this study. Therefore, we have added this limitation to the corresponding section in the Discussion:

<u>Line 539-543:</u>

"Finally, inflammatory-mediated cardiac remodeling was not characterized in this study. As such, the improved autonomic tone and electrical stability observed with RTX treatment may reflect a combination of direct neurally-mediated benefit and indirect anti-inflammatory benefit."

d) What is the expected unit for BRS? line 204 uses mmHg/ms, while Figure 3F states ms/mmHg.

We thank the reviewer for bringing this to our attention. The correct unit for BRS is ms/mmHg. This has since been corrected in the manuscript.

e) In Figure 1A, the pink cohort is labeled simply as "Vehicle," although this group are actually animals that received vehicle prior to MI. Please relabel this group as "Vehicle + MI").

Thank you for noting this. We have since relabeled the "Vehicle + MI" group in Figure 1A.

<u>Response to Reviewer 6:</u>

I co-reviewed this manuscript with one of the reviewers who provided the listed reports. This is part of the Nature Communications initiative to facilitate training in peer review and to provide appropriate recognition for Early Career Researchers who co-review manuscripts.

We thank the reviewer for his/her evaluation of our manuscript.

REFERENCES

1. Shanks, J., de Morais, S.D.B., Gao, L., Zucker, I.H. & Wang, H.J. TRPV1 (Transient Receptor Potential Vanilloid 1) Cardiac Spinal Afferents Contribute to Hypertension in Spontaneous Hypertensive Rat. *Hypertension* **74**, 910-920 (2019).

2. Szabo, T., Olah, Z., Iadarola, M.J. & Blumberg, P.M. Epidural resiniferatoxin induced prolonged regional analgesia to pain. *Brain Res* **840**, 92-98 (1999).

3. Kim, N.H., Lee, S.H. & Lee, S.J. Percutaneous transforaminal epidural injection method in an experimental rat: minimally invasive drug delivery method to spinal epidural space. *Ann Rehabil Med* **36**, 640-647 (2012).

4. Kummer, W., Fischer, A., Kurkowski, R. & Heym, C. The sensory and sympathetic innervation of guinea-pig lung and trachea as studied by retrograde neuronal tracing and double-labelling immunohistochemistry. *Neuroscience* **49**, 715-737 (1992).

5. Belvisi, M.G. Overview of the innervation of the lung. *Curr Opin Pharmacol* **2**, 211-215 (2002).

6. Springall, D.R., *et al.* Retrograde tracing shows that CGRP-immunoreactive nerves of rat trachea and lung originate from vagal and dorsal root ganglia. *J Auton Nerv Syst* **20**, 155-166 (1987).

7. Kim, S.H., *et al.* Mapping of the Sensory Innervation of the Mouse Lung by Specific Vagal and Dorsal Root Ganglion Neuronal Subsets. *eNeuro* **9** (2022).

8. Weaver, L.C. Cardiopulmonary sympathetic afferent influences on renal nerve activity. *Am J Physiol* **233**, H592-599 (1977).

9. Kostreva, D.R., Hopp, F.A. & Kampine, J.P. Depressor responses to stimulation of sympathetic afferents in monkeys and dogs. *Am J Physiol* **240**, R23-28 (1981).

10. Wang, Y., Soukhova, G., Proctor, M., Walker, J. & Yu, J. Bradykinin causes hypotension by activating pulmonary sympathetic afferents in the rabbit. *J Appl Physiol (1985)* **95**, 233-240 (2003).

11. Soukhova-O'Hare, G.K., Zhang, J.W., Gozal, D. & Yu, J. Bradykinin B2 receptors mediate pulmonary sympathetic afferents induced reflexes in rabbits. *Life Sci* **78**, 1990-1997 (2006).

12. Hooper, J.S., *et al.* Characterization of cardiovascular reflexes evoked by airway stimulation with allylisothiocyanate, capsaicin, and ATP in Sprague-Dawley rats. *J Appl Physiol (1985)* **120**, 580-591 (2016).

13. Shanks, J., *et al.* Sympatho-excitatory response to pulmonary chemosensitive spinal afferent activation in anesthetized, vagotomized rats. *Physiol Rep* **6**, e13742 (2018).
